# Prevalence of Germline Sequence Variations Among Patients With Pancreatic Cancer in China

**DOI:** 10.1001/jamanetworkopen.2021.48721

**Published:** 2022-02-16

**Authors:** Lingdi Yin, Jishu Wei, Zipeng Lu, Shimeng Huang, Hao Gao, Jianmin Chen, Feng Guo, Min Tu, Bin Xiao, Chunhua Xi, Kai Zhang, Qiang Li, Junli Wu, Wentao Gao, Kuirong Jiang, Jun Yu, Yi Miao

**Affiliations:** 1 Pancreas Center, The First Affiliated Hospital of Nanjing Medical University, Nanjing, China; 2 Pancreas Institute of Nanjing Medical University, Nanjing, China; 3 Department of Surgery, Johns Hopkins University School of Medicine, Baltimore, Maryland; 4 The Pancreatic Cancer Precision Medicine Center of Excellence, Johns Hopkins University School of Medicine, Baltimore, Maryland; 5 The Sidney Kimmel Comprehensive Cancer Center, Johns Hopkins University School of Medicine, Baltimore, Maryland

## Abstract

**Importance:**

A higher incidence of pancreatic cancer has been reported in the Chinese population compared with the White population, but genetic differences are unknown to date. Large-sample germline testing for both familial and sporadic pancreatic cancers has been conducted predominantly in White populations, whereas similar studies in Chinese populations are limited.

**Objective:**

To assess the prevalence of germline sequence variations in patients with pancreatic diseases in China.

**Design, Setting, and Participants:**

This genetic association study was a case series that included genetic data from patients with pancreatic ductal adenocarcinoma (PDAC) or non-PDAC pancreatic diseases seen at The First Affiliated Hospital of Nanjing Medical University in Nanjing, China, between January 2006 and December 2017 (Nanjing cohort). Comparator group data were obtained for a US cohort from Johns Hopkins Hospital (JHH), a population from East Asia from the Exome Aggregation Consortium (ExAC) database, and the larger population from China from the ChinaMAP database. Data were updated and analyzed in July 2021.

**Main Outcomes and Measures:**

Next-generation sequencing technology was used to examine the prevalence of deleterious variations in 59 genes of the included Chinese patients with DNA extracted from peripheral blood samples. The Fisher exact test was used to assess differences among the frequencies of germline variations in the study patients vs the comparator groups.

**Results:**

A total of 1009 patients with PDAC (627 [62.1%] male; mean [SD] age, 62.8 [10.2] years) and 885 with non-PDAC diseases (477 [53.9%] male; mean [SD] age, 52.0 [15.9] years) from the Nanjing cohort were included for genetic analysis; all were Han Chinese individuals. Pathogenic variations were detected in 63 patients with PDAC (6.2%; 95% CI, 4.7%-7.7%). Variations in *BRCA2* (odds ratio [OR], 3.2; 95% CI, 1.4-7.7; *P* = .008) and *PALB2* (OR, 5.2; 95% CI, 1.6-17.0; *P* = .007) were significantly associated with pancreatic risk in the Nanjing cohort. Pathogenic variants of genes associated with homologous recombination DNA damage repair, including *ATM*, *BRCA1*/2, *PALB2*, *BRIP1*, *FANCA*, *FANCC*, *RAD51D*, and *XRCC2*, were found in 34 patients with PDAC (3.4%). No Ashkenazi Jewish–specific *BRCA2* variation (p.Ser1982fs) was detected. The odds ratio of a *SPINK1* variation in patients with PDAC was 3.2 (95% CI, 1.8-5.7; *P* < .001) in the Nanjing cohort compared with the ExAC cohort. Variations in the pancreatic secretory enzyme genes *CPA1* and *CPB1* were not detected in the Nanjing cohort.

**Conclusions and Relevance:**

In this genetic association study, sporadic pancreatic cancer was associated with pathogenic germline variations in a cohort from China. These findings provide insights into the genetic background of pancreatic cancer in the Han Chinese population with PDAC.

## Key Points

**Question:**

What is the prevalence of germline sequence variations among patients with pancreatic ductal adenocarcinoma (PDAC) in China?

**Findings:**

In this genetic association study of 1009 Chinese patients with PDAC and 885 with non-PDAC diseases, pathogenic sequence variations were detected in 6.2% of patients with PDAC; *SPINK1* and *CFTR* variations were associated with higher risk of PDAC. Variations in the pancreatic secretory enzyme genes *CPA1* and *CPB1* were not detected in the study cohort.

**Meaning:**

In this study, sporadic pancreatic cancer in a cohort from China was associated with pathogenic germline variations.

## Teaser

This genetic association study uses next-generation sequencing technology to assess the prevalence of deleterious sequence variations in patients with pancreatic ductal adenocarcinoma in China.

## Introduction

Pancreatic cancer is a deadly disease with poor prognoses. There are approximately 95 000 new cases of pancreatic ductal adenocarcinoma (PDAC) per year in China, making it the sixth leading cause of cancer-related death in China.^1^ Despite improvements in surgical and systemic treatment, the 5-year survival rate remains only 9%.^1^ In its early stages, pancreatic cancer is largely asymptomatic, and approximately 80% of patients are not diagnosed until the cancer has reached an advanced stage that limits therapeutic options.^2^ Therefore, the early detection of pancreatic cancer is essential to improvement in its prognosis.

Pathogenic germline sequence variations in patients with pancreatic cancer mainly occur in patients with familial pancreatic cancer; however, only approximately 10% of patients with pancreatic cancer have an associated family history.^3^ Carriers of variations may have access to more targeted drugs for cancer-associated genes.^4^ Previous studies^5,6^ showed that *BRCA* carriers from the US, mainly White individuals with the *BRCA1*/*2* variation with sporadic PDAC, had worse survival after pancreatectomy than did their counterparts with wild-type *BRCA*. These *BRCA1/2* carriers also had markedly improved survival if treated with platinum-based chemotherapy.^5,6^ In addition, the Pancreas Cancer Olaparib Ongoing trial^7^ showed that patients with metastatic cancer who had germline *BRCA1/2* variations tended to have improved progression-free survival when treated with maintenance olaparib after first-line platinum-based chemotherapy.

A study^8^ in 2019 showed that native Hawaiian (relative risk [RR], 1.60; 95% CI, 1.30-1.98), Japanese American (RR, 1.33; 95% CI, 1.15-1.54), and African American (RR, 1.20; 95% CI, 1.01-1.42) individuals had a higher risk of developing PDAC compared with non-Hispanic White individuals in the US. However, the reports of germline variants for PDAC were mostly from the non-Hispanic White population. The prevalence of germline variations in the Chinese population and racial and ethnic disparities of germline variations between Chinese patients and the US population are unknown. We conducted a study using targeted next-generation sequencing technology to assess the prevalence of deleterious variations in Chinese patients and explored whether there were differences in sequence variations associated with pancreatic cancer between the Nanjing cohort and cohorts from the US, East Asia, and a larger sample from China.

## Methods

### Patients and Specimens

This genetic association study included patients seen at the Pancreas Center of The First Affiliated Hospital of Nanjing Medical University in Nanjing, China, between January 2006 and December 2017. All peripheral blood samples for DNA extraction were collected and stored in the center’s Pancreas Biobank after written informed consent was obtained from participants.^9^ Samples were collected preoperatively in the operation room or at the clinic visit. All the available samples in the Pancreas Biobank during the study period were included. All patients in the Nanjing cohort were from the Han Chinese population. Personal and family history was obtained from medical records. This study was approved by the institutional review board at The First Affiliated Hospital of Nanjing Medical University and followed the Strengthening the Reporting of Observational Studies in Epidemiology (STROBE) and Strengthening the Reporting of Genetic Association Studies (STREGA) reporting guidelines.

### DNA Isolation

According to the manufacturer’s instructions, genomic DNA was extracted from the peripheral blood samples obtained from all patients using the QIAamp DNA Mini Kit (QIAGEN). The DNA samples were quantified using Qubit assay kits and a Qubit fluorometer (ThermoFisher Scientific).

### Targeted DNA Sequencing

Fifty-nine genes were sequenced using a 59-gene AmpliSeq custom panel (ThermoFisher Scientific). Next-generation sequencing was performed using the Ion GeneStudio S5 System (ThermoFisher Scientific) according to the manufacturer’s protocol. These genes were either known pancreatic cancer susceptibility genes, known cancer susceptibility genes, or candidate pancreatic cancer susceptibility genes. Sixteen of these genes (*ARID1A*, *ATM*, *BRCA1*/2, *PALB2*, *BRIP1*, *ERCC4*, *FANCA*, *FANCC*, *FANCG*, *FANCL*, *FANCJ*, *RAD51C*, *RAD51D*, *RECQL4*, *XRCC2*, and *XRCC3*) are associated with the homologous recombination DNA damage repair (HR-DDR) pathway. Pancreatitis-associated genes were also included. Amplicon coverage is shown in eTable 1 in the Supplement. Next-generation sequencing details are described in the eAppendix in the Supplement. Deleterious variations were compared with those in the ChinaMAP database,^10^ which included whole-genome sequencing data for 10 588 individuals from the population of China.^11^

### Sanger Sequencing

We downloaded the target sequence from the University of California, Santa Cruz, Genome Browser,^12^ inputted the target sequence into Primer Premier 5 software (PREMIER Biosoft), and selected the appropriate and synthesized primers for the Sanger sequencing. All deleterious variations were confirmed and validated by the ABI3730 sequencer (ThermoFisher Scientific). The Sanger sequencing primer list is shown in eTable 2 in the Supplement.

### Statistical Analysis

Analyses were conducted using SPSS, version 21.0 (IBM). The 95% CIs for the percentages of samples with a germline variation were calculated with the modified Wald method. Because of the small number of patients with germline variations, we used the Fisher exact test to assess the difference between the frequencies of germline variations in the study patients and in individuals without cancer in the population from East Asia in the Exome Aggregation Consortium (ExAC)^13^ database, US patients with sporadic PDAC from Johns Hopkins Hospital (JHH),^5^ and individuals without pancreatic cancer from the population of China from the ChinaMAP database. Odds ratios (ORs), SEs, and 95% CIs were calculated according to Altman.^14^ In the JHH cohort,^5^ patients with a pathologic diagnosis of PDAC were sequenced with a germline panel via the same platform used in this study. A cohort with pancreatic cancer from The Cancer Genome Atlas (TCGA) with sequence variation data was also used as comparison group. Two-tailed *P* < .05 was considered to be statistically significant. Bonferroni correction was used when multiple testing was performed. Data were updated and analyzed in July 2021.

## Results

### Patient Characteristics

In the Nanjing cohort, 1009 patients were diagnosed with PDAC (627 [62.1%] male; mean [SD] age, 62.8 [10.2] years) and 885 patients were diagnosed with non-PDAC diseases (477 [53.9%] male; mean [SD] age, 52.0 [15.9] years), including pancreatic neuroendocrine tumors (119 [13.4%]), gastrointestinal stromal tumors (11 [1.2%]), acute (355 [37.8%]) or chronic pancreatitis (98 [11.1%]), intraductal papillary mucinous neoplasms (IPMNs) (70 [7.9%]), mucinous cystic neoplasms (44 [5.0%]), serous cystic neoplasms (32 [3.6%]), solid pseudopapillary tumors (56 [6.3%]), ampullary carcinoma (31 [3.5%]), cholangiocarcinoma (35 [3.9%]), and duodenal adenocarcinoma (34 [3.8%]) (Table 1). Only 9 of the patients with PDAC (0.9%) had at least 1 first-degree relative diagnosed with pancreatic cancer, and 1000 patients (99.1%) had sporadic pancreatic cancer. Thirty-two patients (3.2%) had a history of other tumors, such as breast, prostate, or colon cancer.

**Table 1.** Characteristics of Patients in the Nanjing and JHH Cohorts^a^

Characteristic	Nanjing cohort			JHH cohort	
	Patients with PDAC (n = 1009)	Patients with non-PDAC diseases (n = 885)	Patients who underwent pancreatectomy (n = 939)	Patients with PDAC (n = 854)	Patients with non-PDAC diseases (n = 339)
Age, y					
Mean (SD)	62.8 (10.2)	52.0 (15.9)	NA	65.0 (10.9)	60.1 (14.1)
Median (range)	65 (16-82)	65 (10-85)	NA	NA	NA
Sex					
Female	382 (37.9)	408 (46.1)	NA	399 (46.7)	158 (46.6)
Male	627 (62.1)	477 (53.9)	NA	455 (53.3)	181 (53.4)
Disease					
PDAC	690 (68.4)	NA	NA	854 (100)	NA
Acute pancreatitis	NA	355 (37.8)	0	NA	NA
Chronic pancreatitis	NA	98 (11.1)	65 (6.9)	NA	NA
Ampullary carcinoma	NA	31 (3.5)	29 (3.1)	NA	NA
Cholangiocarcinoma	NA	35 (3.9)	26 (2.8)	NA	47 (13.9)
Duodenal adenocarcinoma	NA	34 (3.8)	32 (3.4)	NA	54 (15.9)
Pancreatic neuroendocrine tumor	NA	119 (13.4)	112 (11.9)	NA	NA
Gastrointestinal stromal tumor	NA	11 (1.2)	9 (1.0)	NA	NA
Intraductal papillary mucinous neoplasm	NA	70 (7.9)	67 (7.1)	NA	NA
Mucinous cystic neoplasm	NA	44 (5.0)	43 (4.6)	NA	NA
Serous cystic neoplasm	NA	32 (3.6)	28 (3.0)	NA	NA
Solid pseudopapillary tumor	NA	56 (6.3)	56 (6.0)	NA	4 (1.2)
History of other cancers	32 (3.2)	14 (1.6)	NA	NA	NA
First-degree relative with pancreatic cancer	9 (0.9)	0	NA	77 (9.0)	NA

Abbreviations: JHH, Johns Hopkins Hospital; NA, not available; PDAC, pancreatic ductal adenocarcinoma.

^a^ Data are presented as the number (percentage) of individuals unless otherwise indicated.

### Deleterious Sequence Variations in Chinese Patients With PDAC

In the Nanjing cohort, deleterious variations were detected in 63 patients with PDAC (6.2%; 95% CI, 4.7%-7.7%) (Figure, A). Deleterious variations associated with cancer according to the ClinVar database of the National Center for Biotechnology Information^15^ were detected in 35 patients (3.5%; 95% CI, 2.3%-4.6%) (Figure, A, and Table 2). Variations of pancreatic cancer susceptibility genes were detected in 24 patients (2.4%; 95% CI, 1.4%-3.3%). These variants included 9 *BRCA2* variations (0.9%). No Ashkenazi Jewish–specific *BRCA2* variations (p.Ser1982fs) were detected. *BRCA1* variations were detected in 3 patients (0.3%); the total number of *BRCA* variation carriers was 12 (1.2%). *ATM* variations were detected in 5 patients (0.5%). *PALB2* variations were detected in 6 patients (0.6%). The prevalence of variations in HR-DDR pathway–related genes (*ATM*, *BRCA1*/2, *PALB2*, *BRIP1*, *FANCA*, *FANCC*, *RAD51D*, and *XRCC2*) was 3.4% (34 patients) (Table 3). Another gene with a variation, *TP53*, was detected in 1 patient with PDAC (0.1%). In addition, variations in the pancreatitis-related genes *CFTR* (8 cases [0.8%]) and *SPINK1* (21 cases [2.1%]) were frequent in patients with PDAC (Table 2). All *SPINK1* variations were c.194 + 2T>C. The recurrent variations in the *CFTR* gene included p.Arg74Trp (2 cases [0.2%]) and p.Gly970Asp (3 cases [0.3%]). Most of these variations were absent or rare in the 10 588 individuals in the ChinaMap cohort (6864 [64.8%] male; mean age, 54.2 years); for example, only 114 patients [1.1%] in the ChinaMap cohort had a *SPINK1* c.194 + 2T>C variation (eTable 3 in the Supplement). Numerous variants of uncertain significance were also identified in the ChinaMap cohort (eTable 4 in the Supplement).

**Figure.**
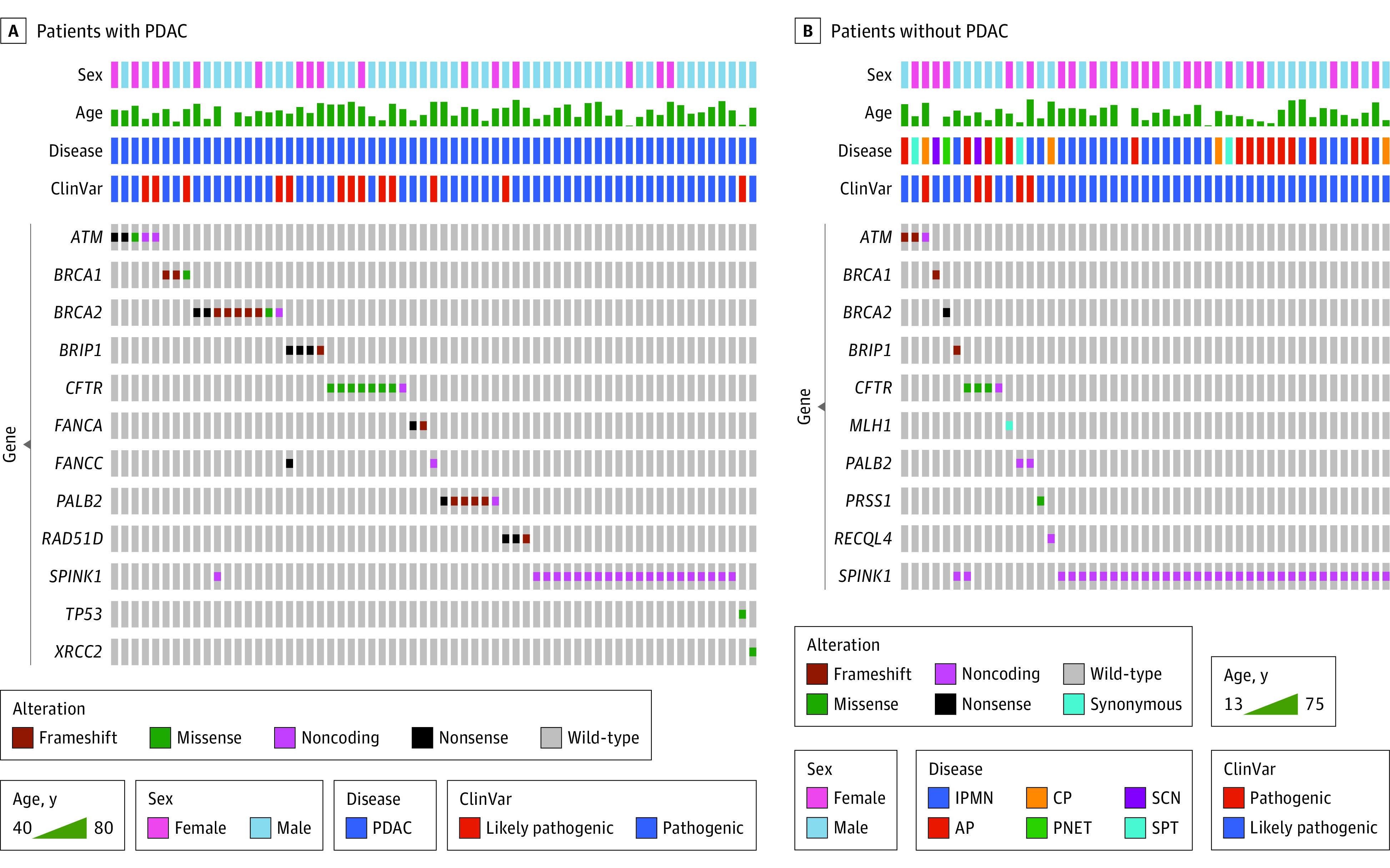
Distribution of Pathogenic Germline Variants Across Genes in Patients With Pancreatic Ductal Adenocarcinoma (PDAC) and With Non-PDAC Diseases ClinVar indicates the pathogenic classification according to the ClinVar database.^15^ AP, acute pancreatitis; CP, chronic pancreatitis; IPMN, intraductal papillary mucinous neoplasm; PNET, pancreatic neuroendocrine tumor; SCN, serous cystic neoplasm; and SPT, solid pseudopapillary tumor.

**Table 2.** Cancer-Associated Germline Sequence Variations in Patients With PDAC in the Nanjing Cohort

Case ID	Chromosome position	Sex	Age, y	Gene	Amino acid change	Nucleotide change	Function	ClinVar^a^	Personal history of other disease	Family history of cancer
P1720	chr11:108106561	Female	71	*ATM*	p.Glu166Gln	c.496G>C	Missense	Pathogenic	Negative	Negative
P0251	chr11:108153468	Female	65	*ATM*	p.Tyr1203Ter	c.3609delT	Nonsense	Pathogenic	Negative	Negative
P1659	chr11:108170440	Male	51	*ATM*	Splice	c.5006-1G>A	Noncoding	Likely pathogenic	Negative	Negative
P2296	chr11:108178646	Male	64	*ATM*	p.Cys1899Ter	c.5697C>A	Nonsense	Pathogenic	Negative	Negative
P2281	chr11:108186639	Female	60	*ATM*	Splice	c.6095 + 1G>A	Noncoding	Likely pathogenic	Negative	Negative
P0537	chr17:41197809	Female	66	*BRCA1*	p.Ile1845fs	c.5533_5540delATTGGGCA	Frameshift	Pathogenic	Breast cancer, lymphoma	Negative
P2289	chr17:41219625	Male	66	*BRCA1*	p.Asp1713Asn	c.5137G>A	Missense	Likely pathogenic	Negative	Negative
P1565	chr17:41245390	Male	47	*BRCA1*	p.Glu720fs	c.2157_2158insA	Frameshift	Pathogenic	Negative	Negative
P0907	chr13:32900280	Male	70	*BRCA2*	p.Lys157fs	c.470_474delAGTCA	Frameshift	Pathogenic	Negative	Negative
P1473	chr13:32906915	Male	55	*BRCA2*	p.Lys437fs	c.1310_1313delAAGA	Frameshift	Pathogenic	Negative	Negative
P2349	chr13:32907029	Male	51	*BRCA2*	p.Gln472Ter	c.1414C>T	Nonsense	Pathogenic	Negative	Negative
P0418	chr13:32907371	Female	74	*BRCA2*	p.Lys586Ter	c.1756A>T	Nonsense	Pathogenic	Lung cancer	Negative
P1449	chr13:32911228	Male	61	*BRCA2*	p.Thr915fs	c.2743_2747delACTTG	Frameshift	Pathogenic	Negative	Negative
P0796	chr13:32912337	Male	40	*BRCA2*	p.Val1283fs	c.3847_3848delGT	Frameshift	Pathogenic	Negative	Negative
P1579	chr13:32914102	Female	63	*BRCA2*	p.Lys1872fs	c.5616_5620delAGTAA	Frameshift	Pathogenic	Ovarian cancer	Negative
P0703	chr13:32921033	Male	61	*BRCA2*	p.Arg2336His	c.7007G>A	Missense	Pathogenic	Negative	Negative
P2247	chr13:32944538	Male	64	*BRCA2*	Splice	c.8332-1G>T	Noncoding	Likely pathogenic	Negative	Negative
P2372	chr16:23614914	Male	48	*PALB2*	p.Leu1143fs	c.3426_3427insA	Frameshift	Pathogenic	Negative	Negative
P0375	chr16:23619181	Female	67	*PALB2*	Splice	c.3350 + 4A>G	Noncoding	Pathogenic	Negative	Negative
P1670	chr16:23635403	Male	66	*PALB2*	p.Gln921fs	c.2760_2761insA	Frameshift	Pathogenic	Negative	Negative
P1649	chr16:23641218	Male	77	*PALB2*	p.Arg753Ter	c.2257C>T	Nonsense	Pathogenic	Negative	Negative
P0757	chr16:23641306	Female	69	*PALB2*	p.Met723fs	c.2167_2168delAT	Frameshift	Pathogenic	Negative	Negative
P0732	chr16:23646660	Male	56	*PALB2*	p.Leu403fs	c.1206delT	Frameshift	Pathogenic	Negative	Negative
P0214	chr17:7577545	Male	42	*TP53*	p.Met246Val	c.736A>G	Missense	Likely pathogenic	Negative	Negative
P0493	chr17:33428225	Male	68	*RAD51D*	p.Arg320Ter	c.958C>T	Nonsense	Likely pathogenic	Negative	Father with pancreatic cancer
P0774	chr17:33430317	Female	80	*RAD51D*	p.Arg252Ter	c.754C>T	Nonsense	Pathogenic	Colon polyps	Negative
P1831	chr17:33434458	Male	68	*RAD51D*	p.Lys111fs	c.331_332insTA	Frameshift	Pathogenic	Negative	Negative
P0417	chr17:59857633	Female	75	*BRIP1*	p.Asn643fs	c.1923delT	Frameshift	Pathogenic	Negative	Negative
P0437	chr17:59858254	Male	59	*BRIP1*	p.Arg581Ter	c.1741C>T	Nonsense	Pathogenic	Negative	Negative
P0581	chr17:59858254	Female	69	*BRIP1*	p.Arg581Ter	c.1741C>T	Nonsense	Pathogenic	Negative	Negative
P2568	chr17:59878688	Female	60	*BRIP1*	p.Arg356Ter	c.1066C>T	Nonsense	Pathogenic	Negative	Negative
P0519	chr16:89833603	Male	57	*FANCA*	p.Ser849fs	c.2546delC	Frameshift	Pathogenic	Negative	Negative
P1545	chr16:89866028	Male	49	*FANCA*	p.Gln271Ter	c.811C>T	Nonsense	Pathogenic	Negative	Negative
P0763	chr9:98002929	Male	77	*FANCC*	Splice	c.345 + 2GT>T	Noncoding	Likely pathogenic	Esophageal cancer	Negative
P0437	chr9:98002937	Male	59	*FANCC*	p.Trp113Ter	c.339G>A	Nonsense	Likely pathogenic	Negative	Negative
P1410	chr7:152346380	Male	68	*XRCC2*	p.Arg64Ter	c.190C>T	Missense	Pathogenic	Negative	Negative
P0201	chr5:147207583	Male	51	*SPINK1*	Splice	c.194 + 2T>C	Noncoding	Pathogenic	Negative	Negative
P0212	chr5:147207583	Male	57	*SPINK1*	Splice	c.194 + 2T>C	Noncoding	Pathogenic	Negative	Negative
P0322	chr5:147207583	Male	68	*SPINK1*	Splice	c.194 + 2T>C	Noncoding	Pathogenic	Negative	Negative
P0328	chr5:147207583	Male	73	*SPINK1*	Splice	c.194 + 2T>C	Noncoding	Pathogenic	Negative	Negative
P0357	chr5:147207583	Male	54	*SPINK1*	Splice	c.194 + 2T>C	Noncoding	Pathogenic	Chronic pancreatitis	Negative
P0572	chr5:147207583	Male	75	*SPINK1*	Splice	c.194 + 2T>C	Noncoding	Pathogenic	Negative	Negative
P0727	chr5:147207583	Male	77	*SPINK1*	Splice	c.194 + 2T>C	Noncoding	Pathogenic	Negative	Negative
P0754	chr5:147207583	Male	56	*SPINK1*	Splice	c.194 + 2T>C	Noncoding	Pathogenic	Negative	Negative
P0907	chr5:147207583	Male	70	*SPINK1*	Splice	c.194 + 2T>C	Noncoding	Pathogenic	Negative	Negative
P1048	chr5:147207583	Male	65	*SPINK1*	Splice	c.194 + 2T>C	Noncoding	Pathogenic	Negative	Negative
P1435	chr5:147207583	Female	41	*SPINK1*	Splice	c.194 + 2T>C	Noncoding	Pathogenic	Negative	Negative
P1439	chr5:147207583	Male	54	*SPINK1*	Splice	c.194 + 2T>C	Noncoding	Pathogenic	Negative	Negative
P1501	chr5:147207583	Male	65	*SPINK1*	Splice	c.194 + 2T>C	Noncoding	Pathogenic	Negative	Father with gastric cancer
P1513	chr5:147207583	Female	57	*SPINK1*	Splice	c.194 + 2T>C	Noncoding	Pathogenic	Negative	Negative
P1536	chr5:147207583	Female	77	*SPINK1*	Splice	c.194 + 2T>C	Noncoding	Pathogenic	Negative	Negative
P1555	chr5:147207583	Male	72	*SPINK1*	Splice	c.194 + 2T>C	Noncoding	Pathogenic	Negative	Negative
P1610	chr5:147207583	Male	59	*SPINK1*	Splice	c.194 + 2T>C	Noncoding	Pathogenic	Negative	Negative
P1732	chr5:147207583	Male	61	*SPINK1*	Splice	c.194 + 2T>C	Noncoding	Pathogenic	Negative	Negative
P2209	chr5:147207583	Male	69	*SPINK1*	Splice	c.194 + 2T>C	Noncoding	Pathogenic	Negative	Negative
P2519	chr5:147207583	Male	78	*SPINK1*	Splice	c.194 + 2T>C	Noncoding	Pathogenic	Negative	Negative
P2556	chr5:147207583	Male	64	*SPINK1*	Splice	c.194 + 2T>C	Noncoding	Pathogenic	Chronic pancreatitis	Father with lung cancer
P1689	chr7:117149143	Female	70	*CFTR*	p.Arg74Trp	c.220C>T	Missense	Likely pathogenic	Negative	Negative
P2293	chr7:117149143	Male	49	*CFTR*	p.Arg74Trp	c.220C>T	Missense	Likely pathogenic	Negative	Negative
P1714	chr7:117232086	Male	55	*CFTR*	p.Gly622Asp	c.1865G>A	Missense	Pathogenic	Negative	Negative
P1674	chr7:117246728	Male	73	*CFTR*	p.Gly970Asp	c.2909G>A	Missense	Likely pathogenic	Negative	Negative
P1677	chr7:117246728	Male	77	*CFTR*	p.Gly970Asp	c.2909G>A	Missense	Likely pathogenic	Negative	Negative
P2370	chr7:117246728	Male	74	*CFTR*	p.Gly970Asp	c.2909G>A	Missense	Likely pathogenic	Lymphosarcoma	Negative
P0533	chr7:117251704	Male	73	*CFTR*	p.Arg1070Gln	c.3209G>A	Missense	Pathogenic	Negative	Negative
P1681	chr7:117254665	Male	66	*CFTR*	Splice	c.3368-2A>G	Noncoding	Pathogenic	Negative	Negative

Abbreviation: PDAC, pancreatic ductal adenocarcinomas.

^a^ Pathogenic classification according to the ClinVar database.^15^

**Table 3.** Prevalence of Sequence Variations Among Patients With PDAC in the Nanjing Cohort and Individuals Without Cancer in the ExAC Cohort^a^

Gene	Nanjing cohort (n = 1009)		ExAC population (n = 4327)		Nanjing cohort vs ExAC population, OR (95% CI)	*P* value
	Individuals with variation, No. (%)	Individuals sequenced, No.	Individuals with variation, No. (%)	Individuals sequenced, No., mean^b^		
Hereditary cancer genes	35 (3.5)^c^	1009	94 (9.3)	4154	1.6 (1.0-2.3)	.03
Pancreatic cancer susceptibility genes	24 (2.4)	1009	40 (1.0)	4154	2.5 (1.5-4.2)	<.001^d^
HR-DDR genes	34 (3.4)^c^	1009	79 (1.7)	4154	1.8 (1.2-2.7)	<.001
*ATM*	5 (0.5)	1009	16 (0.4)	4313	1.3 (0.5-3.7)	.57
*BRCA1*	3 (0.3)	1009	6 (0.1)	4317	2.1 (0.5-8.1)	.28
*BRCA2*	9 (0.9)	1009	12 (0.3)	4305	3.2 (1.4-7.7)	.008
*BRIP1*	4 (0.4)	1009	18 (0.4)	4198	0.7 (0.2-2.4)	.56
*FANCA*	2 (0.2)	1009	10 (0.2)	4119	0.8 (0.2-3.7)	.79
*FANCC*	2 (0.2)	1009	1 (0.02)	4321	8.6 (0.8-94.6)	.08
*PALB2*	6 (0.6)	1009	5 (0.1)	4321	5.2 (1.6-17.0)	.007
*RAD51D*	3 (0.3)	1009	11 (0.3)	4321	1.2 (0.3-4.2)	.81
*XRCC2*	1 (0.1)	1009	0 (0)	4288	12.8 (0.5-313.2)	.19
*SPINK1*	21 (2.1)	1009	26 (0.7)	3958	3.2 (1.8-5.7)	<.001^d^
*CFTR*	8 (0.8)	1009	6 (0.1)	4266	5.7 (2.0-16.4)	.001^d^
*TP53*	1 (0.1)	1009	1 (0.03)	3122	3.1 (0.2-49.5)	.42

Abbreviations: ExAC, Exome Aggregation Consortium; HR-DDR, homologous recombination DNA damage repair; OR, odds ratio; PDAC, pancreatic ductal adenocarcinoma.

^a^ All patients in the Nanjing cohort were from the Han Chinese population, and patients in the ExAC cohort were from East Asia.

^b^ The number of individuals sequenced varied for different loci.

^c^ One patient with 2 variants was included as 1 observation.

^d^ Remained significant at *P* < .003 if Bonferroni correction was used.

The cohort from TGCA included 150 patients with PDAC. In the TCGA cohort, the most common genes with variations were *BRCA2, ATM*, and *CHEK2* (eFigure in the Supplement). There was no overlap for *BRCA2* germline variants among the JHH, TGCA, and Nanjing cohorts (eFigure in the Supplement). These variations were also investigated in the healthy individuals from the ChinaMap database; most were not detected (eTable 3 in the Supplement). Advanced disease stage, tumor location, younger age, and sex were not associated with germline variations (eTable 5 in the Supplement).

Of note, only 13 of the 59 genes (22.0%) were detected with deleterious variations in the 1009 patients in the Nanjing cohort. Variation-free genes in the panel included known pancreatic cancer susceptibility genes, such as *CDKN2A*, *MLH1*, *MSH2*, *PRSS1*, and *STK11*; candidate pancreatic cancer susceptibility genes, such as *FANCG*, *FANCL*, *RECQL4*, *XRCC3*, *ERCC4*, *TERT*, *BRIP1*, *BAP1*, *BUB1*, *BUB3*, and *RNF43*; and most of the pancreatitis-associated genes except *SPINK1* and *CFTR*.

### Prevalence of Deleterious Sequence Variations in Patients With PDAC and non-PDAC in the Nanjing Cohort and the JHH Cohort and Patients Without Cancer in the ExAC Cohort

The JHH cohort included 854 patients with PDAC (455 [53.3%] male; mean (SD) age, 65.0 [10.9] years) and 339 patients with non-PDAC diseases (181 [53.4%] male; mean [SD] age, 60.1 [14.1] years); the ExAC cohort included 4327 patients without cancer from East Asia (demographic data not available). The odds of germline deleterious variations involving *BRCA2*, *PALB2*, *SPINK1*, and *CFTR* were significantly greater in patients with pancreatic cancer in the Nanjing cohort than in those without pancreatic cancer from the ExAC cohort (control) (*BRCA2*: OR, 3.2 [95% CI, 1.4-7.7; *P* = .008]; *PALB2*: OR, 5.2 [95% CI, 1.6-17.0; *P* = .007]; *SPINK1*: OR, 3.2 [95% CI, 1.8-5.7; *P* < .001]; and *CFTR:* 5.7 [95% CI, 2.0-16.4; *P* = .001]) (Table 3); the odds of *SPINK1* and *CFTR* stayed significant after Bonferroni correction. Prevalence of variations in pancreatic cancer susceptibility genes, hereditary cancer genes, and HR-DDR pathway–related genes in the Nanjing cohort was significantly higher than that in the ExAC cohort (Table 3). Comparing the pathogenic germline variations found in this study with those in the JHH cohort, we found no significant difference in the prevalence of germline pathogenic variations in most of the genes between the 2 cohorts (eTable 6 in the Supplement). The germline variation rate of *PALB2* was more prevalent in the Nanjing cohort compared with the JHH cohort (6 of 1009 [0.6%] vs 2 of 854 [0.2%]), but the difference was not significant. Of note, sequence variations in the pancreatic secretory enzyme genes *CPA1* and *CPB1* were not detected in the Nanjing cohort.

### Deleterious Sequence Variations in Patients With Non-PDAC Diseases in the Nanjing Cohort

Among patients with non-PDAC diseases in the Nanjing cohort (Table 4, Figure, B, and eTable 7 in the Supplement), 20 of 98 patients with chronic pancreatitis (20.4%) were found to have pathogenic germline variations, including 18 patients (18.4%) with a recurrent variation of *SPINK1* c.194 + 2T>C, 1 patient (1.0%) with both *SPINK1* c.194 + 2T>C and *BRIP1* p.Leu43fs variations, and 1 patient (1.0%) with a *PRSS1* p.Arg122His variation. Four patients (4.1%), 3 (3.1%) with a *SPINK1* c.194 + 2T>C variation and 1 (1.0%) with a *PRSS1* p.Arg122His variation, experienced multiple recurrent pancreatitis episodes after surgical resection for chronic pancreatitis. Germline pathogenic variations were detected in 15 of 355 patients with acute pancreatitis (4.2%), including a recurrent variation of *SPINK1* c.194 + 2T>C in 11 patients (3.1%), *CFTR* variations (p.Arg74Trp and p.Gly970Asp) in 2 patients (0.6%), and cancer susceptibility gene variations (*MLH1* p.Ser577 = and *ATM* p.Phe2558fs) in 2 patients (0.6%). The patient with an *MLH1* p.Ser577 = variation had a history of colorectal cancer, and the patient with an *ATM* p.Phe2558fs variation had a history of gastric cancer. None of these patients had a family history of pancreatitis. In addition, 2 of 70 patients with IPMN (2.8%) had cancer-related variations, and both were splice-site variations (*RECQL4* c.2755 + 1G>A and *ATM* c.3576 + 1G>A). Of note, the patient with IPMN who had an *ATM* variation (c.3576 + 1G>A) was found to have an invasive carcinoma 9 years after initial surgical resection. A cancer-associated variation (*BRCA1* p.Lys1290fs) was detected in 1 of 119 patients with pancreatic neuroendocrine tumors (0.8%), and this patient was diagnosed at the age of 13 years. Variations were detected in 2 patients (6.3%) with serous cystic neoplasms (*BRCA2* p.Tyr1655Ter and *CFTR* c.580-1G>T). Cancer-associated germline variations, namely *ATM* p.Lys468fs and *PALB2* c.2835-1G>C, were detected in 2 of 56 patients with solid pseudopapillary tumors (3.6%). The ChinaMAP database showed that most variations in patients with non-PDAC diseases were not detected in healthy Chinese individuals.

**Table 4.** Germline Sequence Variations in Patients With Non-PDAC Diseases in the Nanjing Cohort

Case ID	Sex	Age, y	Disease	Gene	Chromosome position	Amino acid change	Nucleotide change	Function	ClinVar^a^	History of other disease	Family history of cancer
P1864	Female	67	IPMN^b^	*ATM*	chr11:108151896	Splice	c.3576 + 1G>A	Noncoding	Likely pathogenic	Negative	Negative
P1056	Male	70	IPMN	*RECQL4*	chr8:145738229	Splice	c.2755 + 1G>A	Noncoding	Pathogenic	Rectal cancer	Father with liver cancer
P1067	Male	48	IPMN	*SPINK1*	chr5:147207583	Splice	c.194 + 2T>C	Noncoding	Pathogenic	Negative	Negative
P2558	Male	27	IPMN	*SPINK1*	chr5:147207583	Splice	c.194 + 2T>C	Noncoding	Pathogenic	Negative	Negative
P0871	Male	64	AP	*ATM*	chr11:108202643	p.Phe2558fs	c.7671_7674delGTTT	Frameshift	Pathogenic	Gastric cancer	Negative
P1313	Male	27	AP	*CFTR*	chr7:117149143	p.Arg74Trp	c.220C>T	Missense	Likely pathogenic	Negative	Negative
P1277	Male	37	AP	*CFTR*	chr7:117246728	p.Gly970Asp	c.2909G>A	Missense	Likely pathogenic	Negative	Negative
P1290	Female	42	AP	*MLH1*	chr3:37083822	p.Ser577 =	c.1731G>A	Synonymous	Pathogenic	Colorectal cancer	Negative
P0862	Female	55	AP	*SPINK1*	chr5:147207583	Splice	c.194 + 2T>C	Noncoding	Pathogenic	Negative	Negative
P1122	Male	36	AP	*SPINK1*	chr5:147207583	Splice	c.194 + 2T>C	Noncoding	Pathogenic	Negative	Negative
P1221	Female	29	AP	*SPINK1*	chr5:147207583	Splice	c.194 + 2T>C	Noncoding	Pathogenic	Negative	Negative
P1249	Female	24	AP	*SPINK1*	chr5:147207583	Splice	c.194 + 2T>C	Noncoding	Pathogenic	Negative	Negative
P1265	Male	20	AP	*SPINK1*	chr5:147207583	Splice	c.194 + 2T>C	Noncoding	Pathogenic	Negative	Negative
P1271	Male	51	AP	*SPINK1*	chr5:147207583	Splice	c.194 + 2T>C	Noncoding	Pathogenic	Negative	Negative
P1277	Male	37	AP	*SPINK1*	chr5:147207583	Splice	c.194 + 2T>C	Noncoding	Pathogenic	Negative	Negative
P1280	Male	73	AP	*SPINK1*	chr5:147207583	Splice	c.194 + 2T>C	Noncoding	Pathogenic	Negative	Negative
P1783	Male	34	AP	*SPINK1*	chr5:147207583	Splice	c.194 + 2T>C	Noncoding	Pathogenic	Negative	Negative
P2494	Female	30	AP	*SPINK1*	chr5:147207583	Splice	c.194 + 2T>C	Noncoding	Pathogenic	Negative	Negative
P2509	Male	44	AP	*SPINK1*	chr5:147207583	Splice	c.194 + 2T>C	Noncoding	Pathogenic	Negative	Negative
P2453	Male	49	CP	*BRIP1*	chr17:59937230	p.Leu43fs	c.128_131delTGTT	Frameshift	Pathogenic	Negative	Negative
P0811	Male	31	CP	*PRSS1*	chr7:142459789	p.Arg122His	c.365G>A	Missense	Pathogenic	Negative	Negative
P0802	Female	55	CP	*SPINK1*	chr5:147207583	Splice	c.194 + 2T>C	Noncoding	Pathogenic	Negative	Negative
P0808	Male	53	CP	*SPINK1*	chr5:147207583	Splice	c.194 + 2T>C	Noncoding	Pathogenic	Negative	Negative
P0810	Female	38	CP	*SPINK1*	chr5:147207583	Splice	c.194 + 2T>C	Noncoding	Pathogenic	Negative	Negative
P0814	Male	56	CP	*SPINK1*	chr5:147207583	Splice	c.194 + 2T>C	Noncoding	Pathogenic	Negative	Negative
P0815	Female	62	CP	*SPINK1*	chr5:147207583	Splice	c.194 + 2T>C	Noncoding	Pathogenic	Negative	Negative
P0829	Male	13	CP	*SPINK1*	chr5:147207583	Splice	c.194 + 2T>C	Noncoding	Pathogenic	Negative	Negative
P1014	Female	27	CP	*SPINK1*	chr5:147207583	Splice	c.194 + 2T>C	Noncoding	Pathogenic	Negative	Negative
P1018	Female	44	CP	*SPINK1*	chr5:147207583	Splice	c.194 + 2T>C	Noncoding	Pathogenic	Negative	Negative
P1021	Male	52	CP	*SPINK1*	chr5:147207583	Splice	c.194 + 2T>C	Noncoding	Pathogenic	Negative	Negative
P1029	Male	37	CP	*SPINK1*	chr5:147207583	Splice	c.194 + 2T>C	Noncoding	Pathogenic	Negative	Negative
P1036	Female	41	CP	*SPINK1*	chr5:147207583	Splice	c.194 + 2T>C	Noncoding	Pathogenic	Negative	Negative
P1045	Female	62	CP	*SPINK1*	chr5:147207583	Splice	c.194 + 2T>C	Noncoding	Pathogenic	Negative	Negative
P1046	Female	15	CP	*SPINK1*	chr5:147207583	Splice	c.194 + 2T>C	Noncoding	Pathogenic	Negative	Negative
P1632	Male	75	CP	*SPINK1*	chr5:147207583	Splice	c.194 + 2T>C	Noncoding	Pathogenic	Negative	Negative
P1806	Male	55	CP	*SPINK1*	chr5:147207583	Splice	c.194 + 2T>C	Noncoding	Pathogenic	Negative	Negative
P1811	Female	52	CP	*SPINK1*	chr5:147207583	Splice	c.194 + 2T>C	Noncoding	Pathogenic	Negative	Negative
P1993	Male	39	CP	*SPINK1*	chr5:147207583	Splice	c.194 + 2T>C	Noncoding	Pathogenic	Negative	Negative
P2453	Male	49	CP	*SPINK1*	chr5:147207583	Splice	c.194 + 2T>C	Noncoding	Pathogenic	Negative	Negative
P2512	Female	68	CP	*SPINK1*	chr5:147207583	Splice	c.194 + 2T>C	Noncoding	Pathogenic	Negative	Negative
P2545	Female	75	CP	*PALB2*	chr16:23625413	Splice	c.3114-1G>A	Noncoding	Likely pathogenic	Negative	Negative
P0626	Female	54	CP	*SPINK1*	chr5:147207583	Splice	c.194 + 2T>C	Noncoding	Pathogenic	Negative	Negative
P2445	Female	13	PNET	*BRCA1*	chr17:41243677	p.Lys1290fs	c.3869_3870delAA	Frameshift	Pathogenic	Negative	Negative
P1124	Male	45	PNET	*CFTR*	chr7:117246728	p.Gly970Asp	c.2909G>A	Missense	Likely pathogenic	Negative	Negative
P1862	Female	35	SCN	*BRCA2*	chr13:32913456	p.Tyr1655Ter	c.4965delC	Nonsense	Pathogenic	Negative	Negative
P2592	Male	53	SCN	*CFTR*	chr7:117175301	Splice	c.580-1G>T	Noncoding	Pathogenic	Negative	Negative
P0980	Female	36	SPT	*ATM*	chr11:108121593	p.Lys468fs	c.1402_1403delAA	Frameshift	Pathogenic	Negative	Negative
P1924	Male	22	SPT	*PALB2*	chr16:23634452	Splice	c.2835-1G>C	Noncoding	Likely pathogenic	Negative	Negative
P1119	Female	39	SPT	*SPINK1*	chr5:147207583	Splice	c.194 + 2T>C	Noncoding	Pathogenic	Negative	Negative

Abbreviations: AP, acute pancreatitis; CP, chronic pancreatitis; IPMN, intraductal papillary mucinous neoplasm; PDAC, pancreatic ductal adenocarcinomas; PNET, pancreatic neuroendocrine tumor; SCN, serous cystic neoplasm; SPT, solid pseudopapillary tumor.

^a^ Pathogenic classification according to the ClinVar database.^15^

^b^ This patient was diagnosed with IPMN, underwent resection, and developed an invasive cancer during surveillance.

## Discussion

To our knowledge, this study is the first to provide a large sample of data on germline variations in a Chinese population with sporadic pancreatic cancer. Germline deleterious variations in cancer susceptibility genes, known pancreatic cancer susceptibility genes, and HR-DDR pathway–related genes were found in the study cohort. Rates of deleterious variations in patients with PDAC were significantly higher than those in patients without pancreatic cancer from the ExAC and ChinaMAP databases. In addition to pancreatic cancer, this study also reported germline variations in patients with noncancer pancreatic diseases such as pancreatic neuroendocrine tumors, pancreatitis, and pancreatic cystic tumors; to our knowledge, this is the first study in which most of these data have been reported.

Among the HR-DDR genes, the genes that most frequently have variations include *ATM*, *BRCA1/2*, and *PALB2*.^6^ Germline variations in the *BRCA1/2* genes may cause double-strand DNA damage repair deficiency and are associated with risk for pancreatic cancer.^16^ The Pancreas Cancer Olaparib Ongoing trial^7^ detected *BRCA1/2* variations in 2167 patients with metastatic pancreatic cancer. The prevalence of variations was higher among Black individuals (10.7%) than among White individuals (6.1%), Asian individuals (5.0%), and individuals of other racial and ethnic groups (1.6%), indicating racial and ethnic differences in the variations associated with pancreatic cancer. Studies by Chang et al^17^ and Grant et al^18^ showed that no significant *BRCA2* susceptibility was found in the population of China after comparing the variations of pancreatic cancer susceptibility genes with those obtained from a large sample study in Europe using a whole-exome association analysis. Studies^19,20,21^ from Japan and South Korea have shown the importance of genetic testing for pancreatic cancer, but the sample sizes were small (<100 patients). The present study provides data on germline variations of *BRCA1/2* and HR-DDR genes in a large sample of Han Chinese patients with pancreatic cancer. Of interest, no Ashkenazi Jewish–specific *BRCA2* variation (p.Ser1982fs) was found in this cohort, which suggests that the *BRCA* variation is associated with race and ethnicity, similar to the findings of another study.^22^ In the JHH cohort,^5^ *BRCA2* and *ATM* variations were associated with higher pancreatic cancer risk in the US population, whereas *BRCA2* and *PALB2* variations were significantly associated with pancreatic cancer risk in the Nanjing cohort. *SPINK1* and *CFTR* variations were also associated with higher risk of PDAC among the Chinese population, whereas variations of these 2 genes were not associated with pancreatic cancer in patients in a study from Germany.^23^ Another study^24^ from JHH showed that variations in the pancreatic secretory enzyme genes *CPA1* and *CPB1* were associated with pancreatic cancer, whereas no variations of these 2 genes were detected in the Nanjing cohort. Of importance, variations in the Nanjing cohort and JHH cohort in this study were detected with the same sequencing platform and strategy, which enhanced the credibility of the findings.

A retrospective study^6^ showed that patients with pancreatic cancer who had germline variations in *BRCA1/2* were more sensitive to platinum-based chemotherapy. A follow-up study of that cohort showed that a deleterious variant in DNA-damage-repair genes was associated with improved survival after resection and adjuvant chemotherapy for pancreatic ductal adenocarcinoma.^25^ Olaparib is a poly(adenosine diphosphate ribose) polymerase inhibitor that interferes with DNA repair in tumor cells and is more effective in patients with *BRCA* variations with homologous recombination repair deficiency.^26^ The Pancreas Cancer Olaparib Ongoing trial^7^ showed that olaparib substantially improved disease-free survival among patients with *BRCA* variations who had PDAC that did not progress after platinum-based first-line chemotherapy. Our study may provide a foundation for the treatment of Chinese patients who have germline variations in the HR-DDR pathway and may be used as a reference for further clinical research and clinical trials of personalized medicine for patients with pancreatic cancer. Our findings suggest that patients with sporadic pancreatic cancer in whom germline variations are detected should undergo familial surveillance and screening. In this study, patients who had a germline variation had a higher incidence of sporadic pancreatic cancer. In addition, genes that were not detected with variations were also of importance when considering cost-effectiveness during the panel design. Large-panel germline testing for sporadic PDACs in Chinese patients should be reconsidered.

Our study also showed deleterious germline variants in patients with noncancer pancreatic diseases. Variations were detected in pancreatic cystic tumors, including IPMNs (*ATM*, *RECQL4*, and *SPINK1*), serous cystic neoplasms (*BRCA2* and *CFTR*), and solid pseudopapillary tumors (*ATM*, *PALB2*, and *SPINK1*). Skaro et al^27^ reported that deleterious germline variations of hereditary cancer genes (eg, *ATM*, *BRCA2*, *PALB2*) were found in patients with IPMN associated with concurrent invasive cancer. In the Nanjing cohort, 1 patient with IPMN with an *ATM* splice-site variation (c.3576 + 1G>A) developed an invasive IPMN during long-term follow-up, suggesting that careful lifelong surveillance is needed for all resected IPMN diseases. The outcomes of germline variants for serous cystic neoplasms and solid pseudopapillary tumors was unclear in the present study. Additional large-sample studies of pancreatic cystic tumors are needed to investigate the prevalence of germline variations and their associations with patient outcomes.

Genetic factors are among the main factors associated with chronic pancreatitis.^28^ Hereditary pancreatitis is often accompanied by germline variations in the *PRSS*, *SPINK1*, and *CFTR* genes.^29^ There is a lack of relevant data among the Chinese population. A sequencing study from China^30^ explored the germline variation of patients with idiopathic chronic pancreatitis, showing that the *SPINK1* c.194 + 2T>C variation was present in 56.2% of adolescent and 42.0% of adult patients with idiopathic chronic pancreatitis. Patients with variations are more likely to develop recurrent pancreatitis,^31^ as did some of the patients in the present study. Of patients diagnosed with acute pancreatitis, 3.1% also carried the *SPINK1* c.194 + 2T>C variation, which suggests that in some patients, a new diagnosis of acute pancreatitis might need to be distinguished from idiopathic chronic pancreatitis. An association of the *SPINK1* c.194 + 2T>C variation with chronic pancreatitis has been widely discussed.^32^ However, an association between a *SPINK1* variation and pancreatic cancer has been less frequently reported, especially in the Chinese population. In the Nanjing cohort, 21 patients with PDAC (2.1%) had a *SPINK1* c.194 + 2T>C variation, which was significantly more frequent than in the ChinaMap population (114 patients [1.1%]). Of note, only 2 of the 21 patients (9.5%) had a history of chronic pancreatitis, suggesting that a *SPINK1* variation might be directly associated with pancreatic cancer and not necessarily associated with chronic pancreatitis.^33^

### Limitations

This study has limitations. First, owing to the study’s retrospective nature, clinical information collection was based on medical records. Second, public databases such as ExAC and ChinaMAP were used as controls for this study instead of sequencing the Chinese population with the same sequencing platform and methods. Third, because this study included patients from a surgical center, patients with PDAC included in this study were mostly initialized for radical resection, which does not represent the whole group of patients with PDAC disease, including those with unresectable PDAC. Fourth, although this is the first study, to our knowledge, to report germline variations in Chinese patients with noncancer pancreatic diseases, the sample sizes of these diseases were small, which requires further exploration of their prevalence and clinical significance in a better designed study with a larger patient cohort.

## Conclusions

This is, to our knowledge, the first large-sample study of Chinese patients with pancreatic cancer using next-generation sequencing technology to detect germline variations. We found that sporadic pancreatic cancer in a population from China was associated with pathogenic germline variations. There were small differences in germline variants of PDAC. This is also the first study, to our knowledge, to report genetic data on noncancer pancreatic diseases. Germline variation detection might be useful for the early detection of pancreatic cancer in patients with high-risk disease, such as IPMN, during surveillance and also might provide supportive information for precision medicine in pancreatic cancer to optimize chemotherapeutic treatments and improve outcomes.

## Notes

Supplement. **eAppendix.** Supplementary Materials and Methods **eFigure.** Comparison of Pathogenic Germline Variants Between Nanjing Cohort and TCGA Cohort **eTable 1.** Amplicons Coverage of Panel **eTable 2.** Primers for Sanger Sequencing **eTable 3.** Prevalence of Pathogenic Germline Mutations in PDAC and Chinese Population **eTable 4.** Variants of Uncertain Significance and Novel Variants Detected **eTable 5.** Clinicopathologic Characteristics of Patients With Germline Mutation and Wild-Type Cases **eTable 6.** Comparison of Nanjing Cohort and Johns Hopkins Cohort on the Pathogenic Germline Mutations of PDAC Patients **eTable 7.** Prevalence of Pathogenic Germline Mutations in Non-PDAC and Chinese Population
