# Supplementary material for: Prevalence of Germline Sequence Variations Among Patients With Pancreatic Cancer in China
Source: JAMA Netw Open. 2022 Feb 16;5(2):e2148721. doi: 10.1001/jamanetworkopen.2021.48721 (PMC8851306; doi:10.1001/jamanetworkopen.2021.48721)
Supplement: Supplement. — eAppendix. Supplementary Materials and Methods eFigure. Comparison of Pathogenic Germline Variants Between Nanjing Cohort and TCGA Cohort eTable 1. Amplicons Coverage of Panel eTable 2. Primers for Sanger Sequencing eTable 3. Prevalence of Pathogenic Germline Mutations in PDAC and Chinese Population eTable 4. Variants of Uncertain Significance and Novel Variants Detected eTable 5. Clinicopathologic Characteristics of Patients With Germline Mutation and Wild-Type Cases eTable 6. Comparison of Nanjing Cohort and Johns Hopkins Cohort on the Pathogenic Germline Mutations of PDAC Patients eTable 7. Prevalence of Pathogenic Germline Mutations in Non-PDAC and Chinese Population [file jamanetwopen-e2148721-s001.pdf]

Supplementary Online Content

Yin L, Wei J, Lu Z, et al. Prevalence of germline sequence variations among patients with pancreatic cancer in China. *JAMA Netw Open*. 2022;5(2):e2148721. doi:10.1001/jamanetworkopen.2021.48721

eAppendix. Supplementary Materials and Methods
eFigure. Comparison of Pathogenic Germline Variants Between Nanjing Cohort and TCGA Cohort
eTable 1. Amplicons Coverage of Panel
eTable 2. Primers for Sanger Sequencing
eTable 3. Prevalence of Pathogenic Germline Mutations in PDAC and Chinese Population
eTable 4. Variants of Uncertain Significance and Novel Variants Detected
eTable 5. Clinicopathologic Characteristics of Patients With Germline Mutation and Wild-Type Cases
eTable 6. Comparison of Nanjing Cohort and Johns Hopkins Cohort on the Pathogenic Germline Mutations of PDAC Patients
eTable 7. Prevalence of Pathogenic Germline Mutations in Non-PDAC and Chinese Population

This supplementary material has been provided by the authors to give readers additional information about their work.

© 2022 Yin L et al. *JAMA Network Open*.

eAppendix.

Supplementary Materials and Methods

Panel detail

Known pancreatic cancer susceptibility genes include *BRCA1/2, ATM, PALB2, CDKN2A, MLH1, MSH2, PRSS1, STK11, and TP53*. Known cancer susceptibility genes include *MSH6, PMS2, CDH1, RAD51C, RAD51D, BUB1B, and FANCJ*. Candidate pancreatic cancer susceptibility genes include *FANCA, FANCC, FANCG, FANCL, ARID1A, RECQL4, XRCC2, XRCC3, ERCC4, TERT, BRIP1, BAP1, BUB1, BUB3, and RNF43*. Pancreatitis associated genes include *AMY2A, CEL, CELA2A, CELA3A, CELA3B, CFTR, CLPS, CPA1, CPA2, CPB1, CTRB1, CTRB2, CTRC, ERBB2, GRP, HNF1B, HNF4G, IDO2, NOC2L, PLA2G1B, PNLIP, PNLIPRP2, REG1A, SPINK1, TNS3, TRYP2 and WEE1.*

Targeted DNA Sequencing and data analysis

Twenty ng of DNA (10 ng per primer pool) were used for the AmpliSeq polymerase chain reaction. After the reagent FuPa's digestion, IonCode Barcode Adapters ligation and library clean up (Agencourt AMPure XP Reagent, Beckman Coulter, Brea, CA), libraries were eluted into low Tris-EDTA and quantified (Ion Quantitation Kit, Life-Tech). Emulsion polymerase chain reaction and Ion Sphere Particles (ISPs) enrichment were conducted by Ion Chef. Enriched Ion Sphere Particles were loaded into Ion 530™ Chips for sequencing (Ion GeneStudio S5, Life-Tech). The post-sequencing BAM files were launched in Ion Reporter (version 5.6, Life-Tech) software for variant calling. Alignments were visually verified using Integrative Genomics Viewer (version 2.3, Broad Institute, Cambridge, MA) and NextGENe Viewer (version 2.4, Softgenetics, State College, PA). The functional significance of variants was determined by interrogating the Exome Aggregation Consortium (ExAC) database, ClinVar (updated July 2020) and PubMed. All deleterious mutations were defined with pathogenic or likely pathogenic significances. Germline variants of TCGA pancreatic cancer generated by Huang et al. were

© 2022 Yin L et al. *JAMA Network Open*.

downloaded from the Genomic Data Commons (GDC, https://gdc.cancer.gov/about-data/publications/PanCanAtlas-Germline-AWG) of the National Cancer Institute (NCI)

© 2022 Yin L et al. *JAMA Network Open*.

Figure legends

eFigure. A. Comparison of Pathogenic Germline Variants between Nanjing Cohort and TCGA cohort. B. Loci of *BRCA2* variants and domains in proteins are shown by lollipop structures for 3 cohorts. Protein domains are also shown in different colors. The x axis reflects the number of amino acid residues.

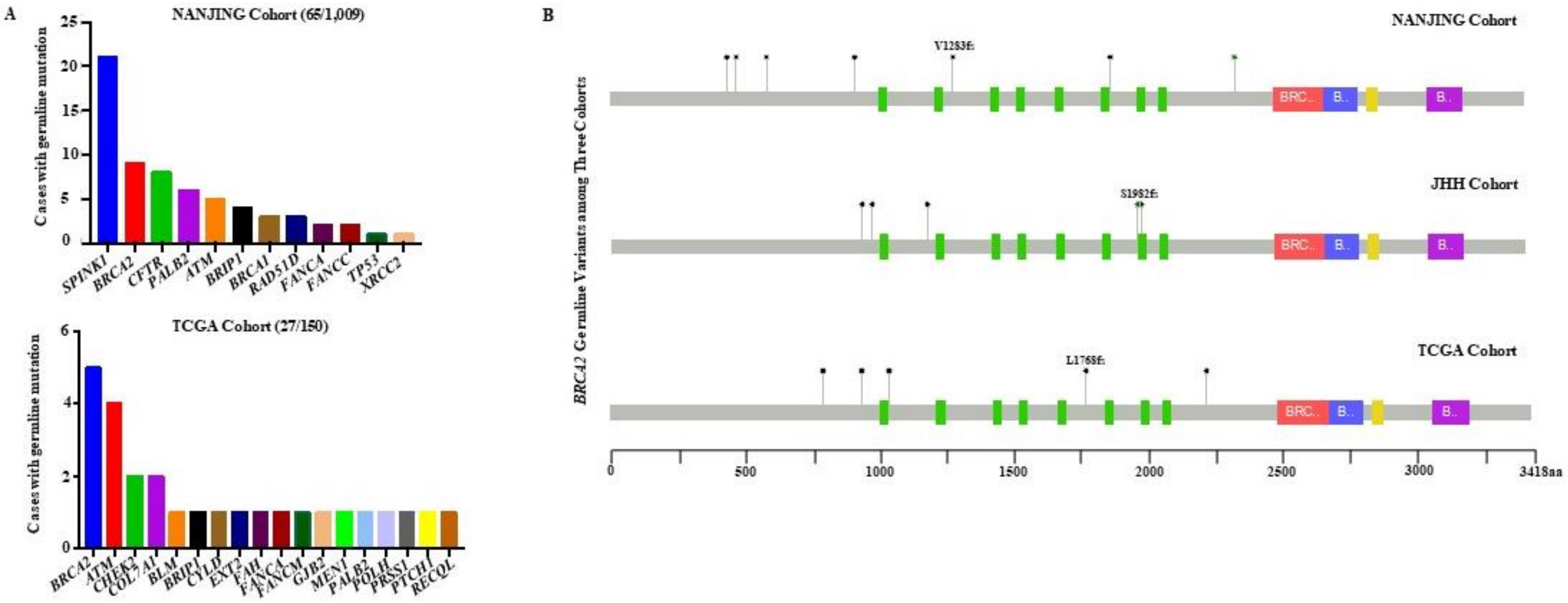

© 2022 Yin L et al. *JAMA Network Open*.

Supplementary Tables S1-S7

eTable 1. Amplicons coverage of panel.

Target_ID	Gene Symbol	Chromosome	Chr_Start	Chr_End	Number of Amplicons
961179	*AMY2A*	chr1	104160057	104160235	2
961181	*AMY2A*	chr1	104160570	104160727	1
961186	*AMY2A*	chr1	104161527	104161735	1
961182	*AMY2A*	chr1	104162170	104162411	2
961178	*AMY2A*	chr1	104163167	104163311	1
961180	*AMY2A*	chr1	104164193	104164326	2
961187	*AMY2A*	chr1	104164410	104164520	1
961184	*AMY2A*	chr1	104166482	104166611	1
961185	*AMY2A*	chr1	104166712	104166848	1
961183	*AMY2A*	chr1	104168173	104168373	1
438331	*ARID1A*	chr1	27022889	27024036	4
436487	*ARID1A*	chr1	27056136	27056359	1
436490	*ARID1A*	chr1	27057637	27058100	2
436486	*ARID1A*	chr1	27059161	27059288	1
436485	*ARID1A*	chr1	27087341	27087592	1
436474	*ARID1A*	chr1	27087869	27087969	1
436481	*ARID1A*	chr1	27088637	27088815	1
436482	*ARID1A*	chr1	27089458	27089781	2
436476	*ARID1A*	chr1	27092706	27092862	2
436484	*ARID1A*	chr1	27092942	27093062	1
436475	*ARID1A*	chr1	27094275	27094495	1
436480	*ARID1A*	chr1	27097604	27097822	1
436471	*ARID1A*	chr1	27098985	27099128	1
436473	*ARID1A*	chr1	27099297	27099483	1
436479	*ARID1A*	chr1	27099831	27099992	2
436489	*ARID1A*	chr1	27100065	27100213	2
436470	*ARID1A*	chr1	27100287	27100394	2
436483	*ARID1A*	chr1	27101465	27101716	2
436477	*ARID1A*	chr1	27100814	27101716	4
436472	*ARID1A*	chr1	27102062	27102203	1
438332	*ARID1A*	chr1	27105508	27107252	7
423445	*ATM*	chr11	108098346	108098428	2
423411	*ATM*	chr11	108098497	108098620	2
423413	*ATM*	chr11	108099899	108100055	1
423396	*ATM*	chr11	108106391	108106566	1
423429	*ATM*	chr11	108114674	108114850	1

© 2022 Yin L et al. *JAMA Network Open*.

423399	*ATM*	chr11	108115509	108115758	1
423446	*ATM*	chr11	108117685	108117859	1
423412	*ATM*	chr11	108119654	108119834	1
423389	*ATM*	chr11	108121422	108121804	2
423419	*ATM*	chr11	108122558	108122763	1
423425	*ATM*	chr11	108123538	108123644	1
423422	*ATM*	chr11	108124535	108124771	1
423394	*ATM*	chr11	108126936	108127072	2
423442	*ATM*	chr11	108128202	108128338	1
423431	*ATM*	chr11	108129707	108129807	1
423410	*ATM*	chr11	108137892	108138074	1
423385	*ATM*	chr11	108139131	108139341	1
423417	*ATM*	chr11	108141785	108141878	1
423407	*ATM*	chr11	108141972	108142138	1
423409	*ATM*	chr11	108143253	108143339	1
423401	*ATM*	chr11	108143443	108143584	2
423435	*ATM*	chr11	108150212	108150340	1
423423	*ATM*	chr11	108151716	108151900	1
423430	*ATM*	chr11	108153431	108153611	1
423406	*ATM*	chr11	108154948	108155205	2
423405	*ATM*	chr11	108158321	108158447	1
423424	*ATM*	chr11	108159698	108159835	1
423415	*ATM*	chr11	108160323	108160533	1
423438	*ATM*	chr11	108163340	108163525	2
423443	*ATM*	chr11	108164034	108164209	2
423398	*ATM*	chr11	108165648	108165791	1
423426	*ATM*	chr11	108168008	108168114	1
423420	*ATM*	chr11	108170435	108170617	1
423444	*ATM*	chr11	108172369	108172521	1
423439	*ATM*	chr11	108173574	108173761	1
423397	*ATM*	chr11	108175396	108175584	1
423402	*ATM*	chr11	108178618	108178716	1
423434	*ATM*	chr11	108180881	108181047	1
423428	*ATM*	chr11	108183132	108183230	1
423387	*ATM*	chr11	108186544	108186643	2
423391	*ATM*	chr11	108186732	108186845	1
423418	*ATM*	chr11	108188094	108188253	1
423392	*ATM*	chr11	108190675	108190790	1
423403	*ATM*	chr11	108192022	108192152	1
423393	*ATM*	chr11	108196031	108196276	1
423433	*ATM*	chr11	108196779	108196957	2

© 2022 Yin L et al. *JAMA Network Open*.

423395	*ATM*	chr11	108198366	108198490	1
423436	*ATM*	chr11	108199742	108199970	2
423427	*ATM*	chr11	108200935	108201153	1
423437	*ATM*	chr11	108202165	108202289	2
423432	*ATM*	chr11	108202600	108202769	1
423404	*ATM*	chr11	108203483	108203632	1
423388	*ATM*	chr11	108204607	108204700	1
423414	*ATM*	chr11	108205690	108205841	1
423440	*ATM*	chr11	108206566	108206693	1
423416	*ATM*	chr11	108213943	108214103	1
423400	*ATM*	chr11	108216464	108216640	1
423390	*ATM*	chr11	108218000	108218097	1
423386	*ATM*	chr11	108224487	108224612	1
423408	*ATM*	chr11	108225532	108225606	1
423441	*ATM*	chr11	108235803	108235950	1
423421	*ATM*	chr11	108236046	108236240	1
438559	*BAP1*	chr3	52436298	52436442	1
438561	*BAP1*	chr3	52436612	52436695	2
438555	*BAP1*	chr3	52436789	52436892	1
438552	*BAP1*	chr3	52437148	52437319	1
438549	*BAP1*	chr3	52437426	52437915	3
438557	*BAP1*	chr3	52438463	52438607	1
438563	*BAP1*	chr3	52439120	52439315	1
438556	*BAP1*	chr3	52439775	52439933	1
438551	*BAP1*	chr3	52440263	52440397	1
438550	*BAP1*	chr3	52440839	52440928	1
438562	*BAP1*	chr3	52441184	52441337	2
438564	*BAP1*	chr3	52441409	52441481	2
438554	*BAP1*	chr3	52441968	52442098	1
438558	*BAP1*	chr3	52442484	52442627	1
438553	*BAP1*	chr3	52443564	52443629	2
438560	*BAP1*	chr3	52443724	52443764	2
438565	*BAP1*	chr3	52443852	52443899	1
422979	*BRCA1*	chr17	41197795	41197824	1
422959	*BRCA1*	chr17	41197689	41197824	1
422972	*BRCA1*	chr17	41199654	41199725	1
422962	*BRCA1*	chr17	41201132	41201216	1
422960	*BRCA1*	chr17	41203074	41203139	1
422958	*BRCA1*	chr17	41209063	41209157	1
422974	*BRCA1*	chr17	41215344	41215395	1
422977	*BRCA1*	chr17	41215885	41215973	1

© 2022 Yin L et al. *JAMA Network Open*.

422965	*BRCA1*	chr17	41219619	41219717	1
422957	*BRCA1*	chr17	41222939	41223260	2
422971	*BRCA1*	chr17	41226342	41226543	1
422969	*BRCA1*	chr17	41228499	41228633	1
422964	*BRCA1*	chr17	41228499	41228636	1
422956	*BRCA1*	chr17	41231345	41231421	1
422970	*BRCA1*	chr17	41234415	41234597	1
422973	*BRCA1*	chr17	41242955	41243054	1
422975	*BRCA1*	chr17	41243446	41246882	12
422961	*BRCA1*	chr17	41246755	41246882	1
422968	*BRCA1*	chr17	41247857	41247944	1
422976	*BRCA1*	chr17	41249255	41249311	1
422978	*BRCA1*	chr17	41251786	41251902	1
422966	*BRCA1*	chr17	41256133	41256283	2
422967	*BRCA1*	chr17	41256879	41256978	1
422954	*BRCA1*	chr17	41258467	41258548	1
422955	*BRCA1*	chr17	41258467	41258555	1
422963	*BRCA1*	chr17	41267737	41267801	1
422953	*BRCA1*	chr17	41276028	41276118	1
423353	*BRCA2*	chr13	32890592	32890669	1
423332	*BRCA2*	chr13	32893208	32893467	1
423344	*BRCA2*	chr13	32899207	32899326	1
423339	*BRCA2*	chr13	32900232	32900292	2
423352	*BRCA2*	chr13	32900373	32900424	2
423337	*BRCA2*	chr13	32900630	32900755	1
423342	*BRCA2*	chr13	32903574	32903634	1
423351	*BRCA2*	chr13	32905050	32905172	1
423331	*BRCA2*	chr13	32906403	32907529	6
423347	*BRCA2*	chr13	32910396	32915338	19
423336	*BRCA2*	chr13	32918689	32918795	2
423345	*BRCA2*	chr13	32920958	32921038	1
423348	*BRCA2*	chr13	32928992	32929430	2
423330	*BRCA2*	chr13	32930559	32930751	1
423354	*BRCA2*	chr13	32931873	32932071	2
423349	*BRCA2*	chr13	32936654	32936835	1
423335	*BRCA2*	chr13	32937310	32937675	2
423338	*BRCA2*	chr13	32944533	32944699	2
423350	*BRCA2*	chr13	32945087	32945242	2
423340	*BRCA2*	chr13	32950801	32950933	1
423346	*BRCA2*	chr13	32953448	32953657	2
423334	*BRCA2*	chr13	32953881	32954055	2

© 2022 Yin L et al. *JAMA Network Open*.

423341	*BRCA2*	chr13	32954138	32954287	1
298058	*BRCA2*	chr13	32968820	32969075	2
423343	*BRCA2*	chr13	32971029	32971186	1
423333	*BRCA2*	chr13	32972293	32972912	3
433507	*BRIP1*	chr17	59760651	59761506	3
433505	*BRIP1*	chr17	59763191	59763531	2
433501	*BRIP1*	chr17	59770785	59770878	1
433506	*BRIP1*	chr17	59793306	59793429	2
433490	*BRIP1*	chr17	59820368	59820500	1
433502	*BRIP1*	chr17	59821787	59821957	1
433497	*BRIP1*	chr17	59853756	59853928	1
433492	*BRIP1*	chr17	59857616	59857767	1
433508	*BRIP1*	chr17	59858195	59858371	1
433493	*BRIP1*	chr17	59861625	59861790	1
433496	*BRIP1*	chr17	59870952	59871095	1
433491	*BRIP1*	chr17	59876455	59876665	1
433504	*BRIP1*	chr17	59878608	59878840	1
433503	*BRIP1*	chr17	59885822	59886123	2
433499	*BRIP1*	chr17	59924456	59924586	1
433498	*BRIP1*	chr17	59926484	59926622	1
433494	*BRIP1*	chr17	59934413	59934597	1
433495	*BRIP1*	chr17	59937151	59937273	1
433500	*BRIP1*	chr17	59938802	59938905	1
884102	*BUB1*	chr2	111395535	111395741	1
869658	*BUB1*	chr2	111397313	111397430	1
1319006	*BUB1*	chr2	111398605	111398774	1
869665	*BUB1*	chr2	111398605	111398787	1
869657	*BUB1*	chr2	111398878	111399046	1
869670	*BUB1*	chr2	111399213	111399385	1
869654	*BUB1*	chr2	111399690	111399816	2
869675	*BUB1*	chr2	111406805	111406959	1
869666	*BUB1*	chr2	111408117	111408366	1
869671	*BUB1*	chr2	111411007	111411105	1
869663	*BUB1*	chr2	111413310	111413498	1
869661	*BUB1*	chr2	111414607	111414699	1
869667	*BUB1*	chr2	111415117	111415227	1
869662	*BUB1*	chr2	111415981	111416102	2
869669	*BUB1*	chr2	111416185	111416324	2
869660	*BUB1*	chr2	111417549	111417618	1
869656	*BUB1*	chr2	111419153	111419423	1
869676	*BUB1*	chr2	111423834	111423996	1

© 2022 Yin L et al. *JAMA Network Open*.

869673	*BUB1*	chr2	111425092	111425287	2
869659	*BUB1*	chr2	111425368	111425431	1
869655	*BUB1*	chr2	111427024	111427135	1
869668	*BUB1*	chr2	111428096	111428150	1
869664	*BUB1*	chr2	111430232	111430439	1
869678	*BUB1*	chr2	111431657	111431806	1
869674	*BUB1*	chr2	111431877	111431947	2
884101	*BUB1*	chr2	111435541	111435577	1
437307	*BUB1B*	chr15	40453416	40453461	1
437328	*BUB1B*	chr15	40457248	40457402	1
437317	*BUB1B*	chr15	40462257	40462327	1
437308	*BUB1B*	chr15	40462732	40462887	1
437322	*BUB1B*	chr15	40468672	40468879	1
437325	*BUB1B*	chr15	40475909	40476089	1
437310	*BUB1B*	chr15	40477360	40477585	1
437320	*BUB1B*	chr15	40477746	40477848	1
437312	*BUB1B*	chr15	40488740	40488980	1
437321	*BUB1B*	chr15	40491810	40491933	1
437318	*BUB1B*	chr15	40492439	40492565	1
437324	*BUB1B*	chr15	40493126	40493186	1
437326	*BUB1B*	chr15	40494600	40494671	2
437316	*BUB1B*	chr15	40494784	40494900	1
437319	*BUB1B*	chr15	40498379	40498664	2
437323	*BUB1B*	chr15	40500832	40500976	1
437309	*BUB1B*	chr15	40501830	40501981	1
437327	*BUB1B*	chr15	40502305	40502416	1
437313	*BUB1B*	chr15	40504694	40504854	1
437315	*BUB1B*	chr15	40505527	40505680	1
437314	*BUB1B*	chr15	40509691	40509873	1
437311	*BUB1B*	chr15	40510651	40510768	1
437306	*BUB1B*	chr15	40512759	40512965	1
884107	*BUB3*	chr10	124914428	124914633	2
884110	*BUB3*	chr10	124915168	124915248	1
884106	*BUB3*	chr10	124917239	124917401	1
884105	*BUB3*	chr10	124919917	124920086	1
884108	*BUB3*	chr10	124921746	124921934	1
884103	*BUB3*	chr10	124922122	124922349	1
884109	*BUB3*	chr10	124923330	124923356	1
884104	*BUB3*	chr10	124924557	124924577	1
433427	*CDH1*	chr16	68771313	68771371	1
433430	*CDH1*	chr16	68772194	68772319	1

© 2022 Yin L et al. *JAMA Network Open*.

433425	*CDH1*	chr16	68835567	68835801	1
433418	*CDH1*	chr16	68842321	68842475	1
433423	*CDH1*	chr16	68842590	68842756	1
433432	*CDH1*	chr16	68844094	68844249	1
433429	*CDH1*	chr16	68845581	68845767	1
433428	*CDH1*	chr16	68846032	68846171	1
433422	*CDH1*	chr16	68847210	68847403	1
433417	*CDH1*	chr16	68849412	68849667	1
2493448	*CDH1*	chr16	68849640	68849667	1
433426	*CDH1*	chr16	68853177	68853333	1
433431	*CDH1*	chr16	68855898	68856133	1
433424	*CDH1*	chr16	68857296	68857534	1
2493447	*CDH1*	chr16	68857325	68857534	1
433421	*CDH1*	chr16	68862071	68862212	1
433420	*CDH1*	chr16	68863551	68863705	1
433419	*CDH1*	chr16	68867187	68867407	1
433711	*CDKN2A*	chr9	21968222	21968246	1
433708	*CDKN2A*	chr9	21968718	21968775	1
433714	*CDKN2A*	chr9	21970895	21971212	1
433713	*CDKN2A*	chr9	21970996	21971212	1
433712	*CDKN2A*	chr9	21974470	21974831	2
433709	*CDKN2A*	chr9	21974671	21974831	2
433710	*CDKN2A*	chr9	21994132	21994335	1
472413	*CEL*	chr9	135937375	135937460	1
472290	*CEL*	chr9	135939785	135939946	2
472292	*CEL*	chr9	135940021	135940154	1
472287	*CEL*	chr9	135940421	135940629	2
472286	*CEL*	chr9	135941911	135942052	1
472295	*CEL*	chr9	135942219	135942337	1
472293	*CEL*	chr9	135942469	135942597	1
472288	*CEL*	chr9	135944053	135944250	2
472296	*CEL*	chr9	135944437	135944651	1
472291	*CEL*	chr9	135945842	135946050	2
472412	*CEL*	chr9	135946368	135947156	2
2059527	*CELA2A*	chr1	15783243	15783293	1
2059526	*CELA2A*	chr1	15783575	15783674	1
2059522	*CELA2A*	chr1	15788050	15788158	1
2059524	*CELA2A*	chr1	15789222	15789361	1
2059525	*CELA2A*	chr1	15789875	15790022	1
2059523	*CELA2A*	chr1	15792488	15792644	1
2059521	*CELA2A*	chr1	15793875	15794038	2

© 2022 Yin L et al. *JAMA Network Open*.

2059520	*CELA2A*	chr1	15798479	15798507	1
2059501	*CELA3A*	chr1	22328162	22328215	1
2059508	*CELA3A*	chr1	22329490	22329586	1
2059507	*CELA3A*	chr1	22331934	22332042	2
2059506	*CELA3A*	chr1	22332149	22332294	2
2059505	*CELA3A*	chr1	22333365	22333512	1
2059502	*CELA3A*	chr1	22333860	22334013	2
2059503	*CELA3A*	chr1	22336192	22336355	1
2059504	*CELA3A*	chr1	22338937	22338965	1
2059535	*CELA3B*	chr1	22303527	22303580	1
2059528	*CELA3B*	chr1	22304856	22304952	1
2059531	*CELA3B*	chr1	22307311	22307419	2
2059529	*CELA3B*	chr1	22307525	22307670	2
2059530	*CELA3B*	chr1	22310181	22310328	1
2059534	*CELA3B*	chr1	22310676	22310829	2
2059532	*CELA3B*	chr1	22313018	22313181	1
2059533	*CELA3B*	chr1	22315749	22315777	1
426194	*CFTR*	chr7	117120143	117120206	1
426191	*CFTR*	chr7	117144301	117144422	1
426188	*CFTR*	chr7	117149082	117149201	1
426201	*CFTR*	chr7	117170947	117171173	2
426204	*CFTR*	chr7	117174324	117174424	1
426207	*CFTR*	chr7	117175296	117175470	1
426202	*CFTR*	chr7	117176596	117176732	2
426210	*CFTR*	chr7	117180148	117180405	2
426200	*CFTR*	chr7	117182064	117182167	1
426206	*CFTR*	chr7	117188689	117188882	1
426198	*CFTR*	chr7	117199512	117199714	1
426193	*CFTR*	chr7	117227787	117227892	1
426196	*CFTR*	chr7	117230401	117230498	1
426205	*CFTR*	chr7	117231982	117232716	3
426190	*CFTR*	chr7	117234978	117235117	2
426197	*CFTR*	chr7	117242874	117242922	1
426203	*CFTR*	chr7	117243580	117243841	1
426209	*CFTR*	chr7	117246722	117246812	1
426214	*CFTR*	chr7	117250567	117250728	1
426189	*CFTR*	chr7	117251629	117251867	2
426199	*CFTR*	chr7	117254661	117254772	1
426192	*CFTR*	chr7	117267570	117267829	1
426211	*CFTR*	chr7	117282486	117282652	1
426208	*CFTR*	chr7	117292890	117292990	1

© 2022 Yin L et al. *JAMA Network Open*.

426212	*CFTR*	chr7	117304736	117304919	1
426195	*CFTR*	chr7	117305507	117305623	1
426213	*CFTR*	chr7	117306956	117307167	1
543639	*CLPS*	chr6	35762917	35763059	1
543637	*CLPS*	chr6	35763502	35763635	1
543638	*CLPS*	chr6	35764580	35764632	1
543636	*CLPS*	chr6	35764976	35765070	1
1387507	*CPA1*	chr7	130020356	130020431	1
1387503	*CPA1*	chr7	130020933	130021025	2
1387502	*CPA1*	chr7	130021465	130021709	2
1387505	*CPA1*	chr7	130021943	130022055	1
1387504	*CPA1*	chr7	130023226	130023338	1
1387501	*CPA1*	chr7	130023519	130023640	1
1387498	*CPA1*	chr7	130024371	130024472	1
1387499	*CPA1*	chr7	130024981	130025191	1
1387506	*CPA1*	chr7	130025674	130025769	1
1387500	*CPA1*	chr7	130027659	130027857	1
1480947	*CPA2*	chr7	129906716	129906797	1
1480951	*CPA2*	chr7	129908763	129908855	1
1480946	*CPA2*	chr7	129909503	129909648	1
1480948	*CPA2*	chr7	129910527	129910633	1
1480945	*CPA2*	chr7	129912910	129913022	1
1480953	*CPA2*	chr7	129914983	129915092	1
1480950	*CPA2*	chr7	129916462	129916583	1
1480949	*CPA2*	chr7	129917660	129917761	1
1480952	*CPA2*	chr7	129919297	129919507	1
1480944	*CPA2*	chr7	129921938	129922033	1
1480943	*CPA2*	chr7	129929394	129929592	1
2059511	*CPB1*	chr3	148545605	148545686	2
2059518	*CPB1*	chr3	148545783	148545869	2
2059510	*CPB1*	chr3	148552279	148552414	1
2059512	*CPB1*	chr3	148558467	148558577	2
2059513	*CPB1*	chr3	148558655	148558767	2
2059517	*CPB1*	chr3	148559604	148559716	1
2059509	*CPB1*	chr3	148562259	148562380	2
2059519	*CPB1*	chr3	148562458	148562559	2
2059516	*CPB1*	chr3	148563205	148563418	1
2059514	*CPB1*	chr3	148575238	148575333	1
2059515	*CPB1*	chr3	148577596	148577794	1
961336	*CTRB1*	chr16	75252900	75252962	1
961338	*CTRB1*	chr16	75256660	75256774	2

© 2022 Yin L et al. *JAMA Network Open*.

961337	*CTRB1*	chr16	75256861	75256951	1
961339	*CTRB1*	chr16	75257033	75257122	2
961341	*CTRB1*	chr16	75257340	75257531	2
961342	*CTRB1*	chr16	75257947	75258091	1
961340	*CTRB1*	chr16	75258597	75258769	1
1237203	*CTRB2*	chr16	75238053	75238225	1
1237208	*CTRB2*	chr16	75238665	75238809	2
1237206	*CTRB2*	chr16	75239225	75239416	2
1237202	*CTRB2*	chr16	75239634	75239723	2
1237205	*CTRB2*	chr16	75239805	75239895	1
1237204	*CTRB2*	chr16	75239982	75240096	2
1237207	*CTRB2*	chr16	75240982	75241044	1
774602	*CTRC*	chr1	15764955	15765005	1
774603	*CTRC*	chr1	15766790	15766892	1
774601	*CTRC*	chr1	15766983	15767091	2
774600	*CTRC*	chr1	15768937	15769073	1
774599	*CTRC*	chr1	15769908	15770055	1
774604	*CTRC*	chr1	15771095	15771251	1
774598	*CTRC*	chr1	15772086	15772249	1
774605	*CTRC*	chr1	15773064	15773089	1
1141491	*ERBB2*	chr17	37855807	37855845	1
438716	*ERBB2*	chr17	37856486	37856569	1
438721	*ERBB2*	chr17	37863254	37863399	1
438723	*ERBB2*	chr17	37863237	37863399	1
438702	*ERBB2*	chr17	37864568	37864792	1
438713	*ERBB2*	chr17	37865565	37865710	1
438710	*ERBB2*	chr17	37866060	37866139	1
438711	*ERBB2*	chr17	37866333	37866459	1
438706	*ERBB2*	chr17	37866587	37866739	1
438705	*ERBB2*	chr17	37868175	37868305	1
438727	*ERBB2*	chr17	37868569	37868706	1
438714	*ERBB2*	chr17	37871533	37871617	2
438704	*ERBB2*	chr17	37871693	37871794	2
438700	*ERBB2*	chr17	37871987	37872197	1
438724	*ERBB2*	chr17	37872548	37872691	2
438709	*ERBB2*	chr17	37872762	37872863	2
438720	*ERBB2*	chr17	37873567	37873738	1
1141493	*ERBB2*	chr17	37873567	37873742	1
438718	*ERBB2*	chr17	37876034	37876092	1
438708	*ERBB2*	chr17	37879566	37879715	2
438703	*ERBB2*	chr17	37879785	37879918	2

© 2022 Yin L et al. *JAMA Network Open*.

438701	*ERBB2*	chr17	37880159	37880268	1
438715	*ERBB2*	chr17	37880973	37881169	2
438712	*ERBB2*	chr17	37881296	37881462	1
438722	*ERBB2*	chr17	37881574	37881660	1
438717	*ERBB2*	chr17	37881954	37882111	1
438726	*ERBB2*	chr17	37882809	37882917	1
438707	*ERBB2*	chr17	37883062	37883261	1
438719	*ERBB2*	chr17	37883542	37883805	2
1141492	*ERBB2*	chr17	37883936	37883955	2
438725	*ERBB2*	chr17	37883936	37884302	3
438023	*ERCC4*	chr16	14014017	14014234	2
438025	*ERCC4*	chr16	14015882	14016073	1
438032	*ERCC4*	chr16	14020412	14020618	1
438028	*ERCC4*	chr16	14021879	14022097	2
438029	*ERCC4*	chr16	14024561	14024752	2
438024	*ERCC4*	chr16	14026008	14026147	1
438027	*ERCC4*	chr16	14028043	14028164	1
438022	*ERCC4*	chr16	14028997	14029605	2
438031	*ERCC4*	chr16	14031617	14031720	1
438030	*ERCC4*	chr16	14038574	14038697	1
438026	*ERCC4*	chr16	14041465	14042209	3
426637	*FANCA*	chr16	89805003	89805121	1
1163050	*FANCA*	chr16	89805100	89805121	1
426655	*FANCA*	chr16	89805284	89805387	1
426638	*FANCA*	chr16	89805535	89805702	1
1163049	*FANCA*	chr16	89805531	89805702	1
426639	*FANCA*	chr16	89805880	89805966	1
426646	*FANCA*	chr16	89806396	89806512	1
426668	*FANCA*	chr16	89807206	89807279	1
426652	*FANCA*	chr16	89809202	89809351	1
426635	*FANCA*	chr16	89811361	89811484	1
426626	*FANCA*	chr16	89812986	89813101	1
426629	*FANCA*	chr16	89813233	89813303	1
426643	*FANCA*	chr16	89815061	89815180	1
426628	*FANCA*	chr16	89816132	89816315	2
426669	*FANCA*	chr16	89818540	89818635	1
426632	*FANCA*	chr16	89824979	89825118	1
426647	*FANCA*	chr16	89828351	89828435	1
426636	*FANCA*	chr16	89831292	89831479	2
426642	*FANCA*	chr16	89833543	89833650	1
426645	*FANCA*	chr16	89836239	89836437	1

© 2022 Yin L et al. *JAMA Network Open*.

426648	*FANCA*	chr16	89836568	89836672	1
426656	*FANCA*	chr16	89836966	89837047	1
426664	*FANCA*	chr16	89838080	89838227	1
426633	*FANCA*	chr16	89839673	89839797	1
426653	*FANCA*	chr16	89842144	89842228	1
426666	*FANCA*	chr16	89845203	89845263	2
426667	*FANCA*	chr16	89845345	89845416	2
426650	*FANCA*	chr16	89846271	89846370	1
426649	*FANCA*	chr16	89849261	89849331	2
426658	*FANCA*	chr16	89849409	89849515	2
426663	*FANCA*	chr16	89851256	89851377	1
426654	*FANCA*	chr16	89857805	89857949	1
426630	*FANCA*	chr16	89858329	89858481	2
426661	*FANCA*	chr16	89858873	89858960	1
426634	*FANCA*	chr16	89862308	89862431	1
426627	*FANCA*	chr16	89865481	89865492	1
426662	*FANCA*	chr16	89865568	89865645	1
426660	*FANCA*	chr16	89866007	89866051	1
426641	*FANCA*	chr16	89869661	89869754	1
426640	*FANCA*	chr16	89871682	89871805	1
426657	*FANCA*	chr16	89874696	89874780	1
426644	*FANCA*	chr16	89877109	89877215	1
426665	*FANCA*	chr16	89877331	89877484	1
426659	*FANCA*	chr16	89880922	89881026	1
426631	*FANCA*	chr16	89882279	89882399	1
426651	*FANCA*	chr16	89882939	89883028	0
431076	*FANCC*	chr9	97863983	97864137	1
431083	*FANCC*	chr9	97869342	97869556	1
431089	*FANCC*	chr9	97873472	97873632	1
431078	*FANCC*	chr9	97873739	97873924	2
431075	*FANCC*	chr9	97876905	97876997	1
431080	*FANCC*	chr9	97879591	97879677	1
431077	*FANCC*	chr9	97887362	97887472	1
431085	*FANCC*	chr9	97888805	97888868	1
431079	*FANCC*	chr9	97897622	97897789	1
431086	*FANCC*	chr9	97912199	97912374	1
431088	*FANCC*	chr9	97933355	97933430	1
431082	*FANCC*	chr9	97934313	97934434	1
431087	*FANCC*	chr9	98002925	98003030	1
431084	*FANCC*	chr9	98009708	98009803	1
431081	*FANCC*	chr9	98011403	98011578	1

© 2022 Yin L et al. *JAMA Network Open*.

430455	*FANCG*	chr9	35074099	35074218	1
430450	*FANCG*	chr9	35074362	35074496	1
430451	*FANCG*	chr9	35074918	35075084	1
430449	*FANCG*	chr9	35075270	35075327	2
430454	*FANCG*	chr9	35075456	35075756	3
430453	*FANCG*	chr9	35075953	35076030	1
430461	*FANCG*	chr9	35076423	35076585	1
430459	*FANCG*	chr9	35076715	35076872	1
430462	*FANCG*	chr9	35076962	35077103	1
430458	*FANCG*	chr9	35077255	35077401	1
430457	*FANCG*	chr9	35078132	35078345	1
430452	*FANCG*	chr9	35078596	35078738	1
430456	*FANCG*	chr9	35079142	35079243	1
430460	*FANCG*	chr9	35079432	35079526	1
437900	*FANCL*	chr2	58386894	58386940	1
437903	*FANCL*	chr2	58387237	58387319	1
437891	*FANCL*	chr2	58388651	58388778	1
437901	*FANCL*	chr2	58389995	58390087	2
437904	*FANCL*	chr2	58390158	58390214	2
437893	*FANCL*	chr2	58390563	58390657	1
437895	*FANCL*	chr2	58392853	58393014	1
437894	*FANCL*	chr2	58425708	58425802	1
437896	*FANCL*	chr2	58425723	58425802	1
437902	*FANCL*	chr2	58431259	58431366	1
437890	*FANCL*	chr2	58449071	58449182	1
437897	*FANCL*	chr2	58453857	58453924	1
437899	*FANCL*	chr2	58456943	58457014	1
437898	*FANCL*	chr2	58459183	58459252	1
437892	*FANCL*	chr2	58468347	58468453	1
691778	*GRP*	chr18	56887492	56887641	1
691779	*GRP*	chr18	56892718	56892952	1
691783	*GRP*	chr18	56892718	56892971	1
691782	*GRP*	chr18	56897630	56897694	1
691780	*GRP*	chr18	56897651	56897705	1
691781	*GRP*	chr18	56897630	56897705	1
2493419	*HNF1B*	chr17	36047277	36047400	1
439200	*HNF1B*	chr17	36047369	36047400	1
439191	*HNF1B*	chr17	36059076	36059205	1
439199	*HNF1B*	chr17	36060982	36061187	1
439193	*HNF1B*	chr17	36064918	36065061	1
439197	*HNF1B*	chr17	36070505	36070676	1

© 2022 Yin L et al. *JAMA Network Open*.

439194	*HNF1B*	chr17	36091580	36091826	1
439196	*HNF1B*	chr17	36093544	36093741	1
439198	*HNF1B*	chr17	36093544	36093819	1
439195	*HNF1B*	chr17	36099425	36099635	1
439192	*HNF1B*	chr17	36104526	36104880	2
607347	*HNF4G*	chr8	76452222	76452320	1
607353	*HNF4G*	chr8	76456040	76456219	1
607352	*HNF4G*	chr8	76459816	76459921	2
607351	*HNF4G*	chr8	76463617	76463734	1
607345	*HNF4G*	chr8	76465271	76465437	1
607346	*HNF4G*	chr8	76468211	76468309	1
607348	*HNF4G*	chr8	76470747	76470910	2
607354	*HNF4G*	chr8	76471030	76471277	2
607349	*HNF4G*	chr8	76472573	76472706	1
607350	*HNF4G*	chr8	76476204	76476336	1
742194	*IDO2*	chr8	39792710	39792742	1
742190	*IDO2*	chr8	39806662	39806788	1
742195	*IDO2*	chr8	39821121	39821227	1
742191	*IDO2*	chr8	39836580	39836710	1
742198	*IDO2*	chr8	39840165	39840294	1
742193	*IDO2*	chr8	39843021	39843046	1
742192	*IDO2*	chr8	39845384	39845494	1
742197	*IDO2*	chr8	39847234	39847362	1
742189	*IDO2*	chr8	39862840	39862902	1
742199	*IDO2*	chr8	39871078	39871237	1
742196	*IDO2*	chr8	39872760	39873126	2
427108	*MLH1*	chr3	37035033	37035159	1
252790	*MLH1*	chr3	37038104	37038205	1
427110	*MLH1*	chr3	37042522	37042544	1
427112	*MLH1*	chr3	37042440	37042549	1
427106	*MLH1*	chr3	37045886	37045970	1
427098	*MLH1*	chr3	37048476	37048559	1
427101	*MLH1*	chr3	37050299	37050401	1
427095	*MLH1*	chr3	37053305	37053358	1
427094	*MLH1*	chr3	37053496	37053595	1
427093	*MLH1*	chr3	37055917	37056040	1
427099	*MLH1*	chr3	37055963	37056040	1
427103	*MLH1*	chr3	37058991	37059095	1
427104	*MLH1*	chr3	37061795	37061959	1
427107	*MLH1*	chr3	37067122	37067503	2
427105	*MLH1*	chr3	37070269	37070428	1

© 2022 Yin L et al. *JAMA Network Open*.

427109	*MLH1*	chr3	37081671	37081790	1
427096	*MLH1*	chr3	37083753	37083827	1
427100	*MLH1*	chr3	37089004	37089179	1
427102	*MLH1*	chr3	37090002	37090105	1
427097	*MLH1*	chr3	37090389	37090513	1
427111	*MLH1*	chr3	37091971	37092149	1
1146596	*MSH2*	chr2	47630523	47630546	1
426840	*MSH2*	chr2	47630325	47630546	2
426849	*MSH2*	chr2	47635534	47635699	1
426850	*MSH2*	chr2	47637227	47637516	1
426851	*MSH2*	chr2	47639547	47639704	2
426844	*MSH2*	chr2	47641402	47641562	1
426852	*MSH2*	chr2	47643429	47643573	1
426843	*MSH2*	chr2	47656875	47657085	2
426847	*MSH2*	chr2	47672681	47672801	1
426845	*MSH2*	chr2	47690164	47690298	2
426846	*MSH2*	chr2	47693791	47693952	2
426837	*MSH2*	chr2	47698098	47698206	1
426842	*MSH2*	chr2	47702158	47702414	2
426838	*MSH2*	chr2	47703500	47703715	1
426848	*MSH2*	chr2	47705405	47705663	1
426841	*MSH2*	chr2	47707829	47708015	1
426839	*MSH2*	chr2	47709912	47710093	1
1146597	*MSH6*	chr2	48010367	48010614	2
439820	*MSH6*	chr2	48010367	48010637	2
439811	*MSH6*	chr2	48018060	48018267	1
439815	*MSH6*	chr2	48023027	48023207	1
1146598	*MSH6*	chr2	48026023	48028299	9
439819	*MSH6*	chr2	48025744	48028299	10
439817	*MSH6*	chr2	48030553	48030829	2
439814	*MSH6*	chr2	48032043	48032171	1
439813	*MSH6*	chr2	48032751	48032851	2
439816	*MSH6*	chr2	48033337	48033502	1
439812	*MSH6*	chr2	48033585	48033795	1
439818	*MSH6*	chr2	48033912	48034004	1
2803135	*NOC2L*	chr1	880068	880185	1
2803132	*NOC2L*	chr1	880431	880531	1
2803140	*NOC2L*	chr1	880892	881038	2
2803139	*NOC2L*	chr1	881547	881671	1
2803131	*NOC2L*	chr1	881776	881930	2
2803133	*NOC2L*	chr1	883505	883617	1

© 2022 Yin L et al. *JAMA Network Open*.

2803137	*NOC2L*	chr1	883864	883988	1
2803136	*NOC2L*	chr1	886501	886623	1
2803129	*NOC2L*	chr1	887374	887524	1
2803144	*NOC2L*	chr1	887786	887985	2
2803146	*NOC2L*	chr1	888549	888673	1
2803130	*NOC2L*	chr1	889156	889277	1
2803142	*NOC2L*	chr1	889378	889467	1
2803128	*NOC2L*	chr1	891297	891398	2
2803134	*NOC2L*	chr1	891469	891600	1
2803141	*NOC2L*	chr1	892268	892410	2
2803145	*NOC2L*	chr1	892473	892658	1
2803143	*NOC2L*	chr1	894303	894466	2
2803138	*NOC2L*	chr1	894589	894625	1
423382	*PALB2*	chr16	23614774	23614995	1
423376	*PALB2*	chr16	23619179	23619338	1
423375	*PALB2*	chr16	23625319	23625417	1
423374	*PALB2*	chr16	23632677	23632804	1
423380	*PALB2*	chr16	23634284	23634456	1
423381	*PALB2*	chr16	23635324	23635420	1
423384	*PALB2*	chr16	23637551	23637723	1
423378	*PALB2*	chr16	23640519	23640601	1
423377	*PALB2*	chr16	23640955	23641795	3
423383	*PALB2*	chr16	23646177	23647660	6
423379	*PALB2*	chr16	23649165	23649278	1
423373	*PALB2*	chr16	23649385	23649455	1
423372	*PALB2*	chr16	23652425	23652483	1
2059500	*PLA2G1B*	chr12	120759990	120760125	1
2059498	*PLA2G1B*	chr12	120762731	120762869	1
2059497	*PLA2G1B*	chr12	120763658	120763828	1
2059499	*PLA2G1B*	chr12	120765517	120765561	1
440444	*PMS2*	chr7	6013024	6013178	1
440434	*PMS2*	chr7	6017213	6017393	1
440448	*PMS2*	chr7	6018221	6018332	1
440441	*PMS2*	chr7	6022449	6022627	1
440442	*PMS2*	chr7	6026384	6027256	3
440439	*PMS2*	chr7	6029425	6029591	1
440435	*PMS2*	chr7	6031598	6031693	1
440445	*PMS2*	chr7	6035159	6035269	1
440436	*PMS2*	chr7	6036951	6037059	1
440446	*PMS2*	chr7	6038733	6038911	1
440440	*PMS2*	chr7	6042078	6042272	2

© 2022 Yin L et al. *JAMA Network Open*.

440447	*PMS2*	chr7	6043315	6043428	1
440443	*PMS2*	chr7	6043597	6043694	1
440437	*PMS2*	chr7	6045517	6045667	2
440438	*PMS2*	chr7	6048622	6048655	1
1863828	*PNLIP*	chr10	118305599	118305655	1
1863827	*PNLIP*	chr10	118306800	118306965	1
1863835	*PNLIP*	chr10	118307866	118307999	1
1863834	*PNLIP*	chr10	118310604	118310749	1
1863824	*PNLIP*	chr10	118313233	118313355	1
1863833	*PNLIP*	chr10	118314684	118314814	1
1863829	*PNLIP*	chr10	118314894	118315024	2
1863826	*PNLIP*	chr10	118315506	118315635	1
1863831	*PNLIP*	chr10	118318660	118318800	1
1863832	*PNLIP*	chr10	118319922	118320041	1
1863830	*PNLIP*	chr10	118320978	118321153	1
1863825	*PNLIP*	chr10	118327241	118327315	1
2059536	*PNLIPRP2*	chr10	118380482	118380495	1
1491410	*PNLIPRP2*	chr10	118380812	118380871	1
1491405	*PNLIPRP2*	chr10	118383451	118383615	1
1491415	*PNLIPRP2*	chr10	118385451	118385587	1
1491407	*PNLIPRP2*	chr10	118386369	118386514	1
1491409	*PNLIPRP2*	chr10	118387268	118387390	1
1491411	*PNLIPRP2*	chr10	118389448	118389578	1
1491413	*PNLIPRP2*	chr10	118390743	118390873	1
1491412	*PNLIPRP2*	chr10	118394346	118394475	1
1491403	*PNLIPRP2*	chr10	118396290	118396430	1
1491404	*PNLIPRP2*	chr10	118397874	118397996	1
1491406	*PNLIPRP2*	chr10	118401620	118401798	1
2059537	*PNLIPRP2*	chr10	118404539	118404613	1
472301	*PRSS1*	chr7	142457330	142457380	1
472300	*PRSS1*	chr7	142458400	142458570	2
472297	*PRSS1*	chr7	142459619	142459883	2
472298	*PRSS1*	chr7	142460276	142460423	1
472299	*PRSS1*	chr7	142460713	142460876	1
529716	*RAD51B*	chr14	68290255	68290349	1
529712	*RAD51B*	chr14	68292175	68292299	1
529708	*RAD51B*	chr14	68301791	68301918	1
529715	*RAD51B*	chr14	68331714	68331861	1
529710	*RAD51B*	chr14	68352580	68352710	1
529711	*RAD51B*	chr14	68353732	68353926	2
529714	*RAD51B*	chr14	68758595	68758702	1

© 2022 Yin L et al. *JAMA Network Open*.

529718	*RAD51B*	chr14	68878135	68878249	1
529717	*RAD51B*	chr14	68934883	68934972	1
529709	*RAD51B*	chr14	68944359	68944386	1
529707	*RAD51B*	chr14	68963835	68963862	1
529713	*RAD51B*	chr14	69061196	69061325	1
433487	*RAD51C*	chr17	56769999	56770154	2
433482	*RAD51C*	chr17	56772286	56772555	2
433483	*RAD51C*	chr17	56772286	56772559	2
433485	*RAD51C*	chr17	56774048	56774225	1
433486	*RAD51C*	chr17	56780551	56780695	1
433489	*RAD51C*	chr17	56787214	56787356	1
433484	*RAD51C*	chr17	56798101	56798178	1
433481	*RAD51C*	chr17	56801395	56801466	1
433480	*RAD51C*	chr17	56809839	56809910	1
433488	*RAD51C*	chr17	56811473	56811588	1
439584	*RAD51D*	chr17	33427966	33428060	1
439588	*RAD51D*	chr17	33428214	33428389	1
439586	*RAD51D*	chr17	33430267	33430348	1
439587	*RAD51D*	chr17	33430467	33430568	1
439580	*RAD51D*	chr17	33433399	33433505	1
439583	*RAD51D*	chr17	33434001	33434146	1
439579	*RAD51D*	chr17	33434379	33434471	1
439582	*RAD51D*	chr17	33443872	33444061	1
439581	*RAD51D*	chr17	33445514	33445643	1
439585	*RAD51D*	chr17	33446124	33446196	1
439578	*RAD51D*	chr17	33446545	33446637	2
525756	*RECQL4*	chr8	145736808	145736943	2
525752	*RECQL4*	chr8	145737058	145737177	1
525748	*RECQL4*	chr8	145737288	145737455	1
525746	*RECQL4*	chr8	145737521	145737712	3
525758	*RECQL4*	chr8	145737769	145737949	1
525765	*RECQL4*	chr8	145738019	145738159	1
525753	*RECQL4*	chr8	145738224	145738526	2
1181003	*RECQL4*	chr8	145738595	145738772	1
1181004	*RECQL4*	chr8	145738763	145738869	2
525763	*RECQL4*	chr8	145738949	145739101	2
525761	*RECQL4*	chr8	145739306	145739496	2
525750	*RECQL4*	chr8	145739567	145739751	2
525745	*RECQL4*	chr8	145739820	145739914	2
525760	*RECQL4*	chr8	145740314	145740461	2
525754	*RECQL4*	chr8	145740528	145740631	2

© 2022 Yin L et al. *JAMA Network Open*.

525749	*RECQL4*	chr8	145740704	145740846	1
525755	*RECQL4*	chr8	145741142	145741279	2
525751	*RECQL4*	chr8	145741366	145742153	4
525747	*RECQL4*	chr8	145742428	145742579	1
525762	*RECQL4*	chr8	145742792	145742897	2
525759	*RECQL4*	chr8	145742980	145743024	2
525757	*RECQL4*	chr8	145743079	145743173	1
1060947	*REG1A*	chr2	79347982	79348056	1
1060948	*REG1A*	chr2	79348682	79348811	1
1060951	*REG1A*	chr2	79349108	79349256	1
1060950	*REG1A*	chr2	79349961	79350083	1
1060949	*REG1A*	chr2	79350268	79350346	1
443179	*RNF43*	chr17	56432298	56432352	1
436409	*RNF43*	chr17	56434823	56436189	6
436408	*RNF43*	chr17	56437504	56437617	1
436406	*RNF43*	chr17	56438138	56438310	1
436402	*RNF43*	chr17	56439899	56440014	1
436404	*RNF43*	chr17	56440630	56440772	2
2503706	*RNF43*	chr17	56440881	56440960	1
436401	*RNF43*	chr17	56440881	56440966	1
436410	*RNF43*	chr17	56448266	56448399	1
443178	*RNF43*	chr17	56492681	56492943	1
472305	*SPINK1*	chr5	147204218	147204274	1
472302	*SPINK1*	chr5	147207579	147207696	1
472304	*SPINK1*	chr5	147209156	147209198	1
472303	*SPINK1*	chr5	147211080	147211145	1
435833	*STK11*	chr19	1206907	1207207	2
433376	*STK11*	chr19	1218410	1218504	1
433377	*STK11*	chr19	1219317	1219417	1
433378	*STK11*	chr19	1220366	1220509	2
433370	*STK11*	chr19	1220574	1220721	2
433371	*STK11*	chr19	1221206	1221344	1
433375	*STK11*	chr19	1221942	1222010	2
433372	*STK11*	chr19	1222978	1223176	2
435834	*STK11*	chr19	1226447	1226651	2
434161	*TERT*	chr5	1253837	1253951	1
434171	*TERT*	chr5	1254477	1254625	1
434168	*TERT*	chr5	1255396	1255531	1
434165	*TERT*	chr5	1258707	1258779	1
434170	*TERT*	chr5	1260583	1260720	1
434173	*TERT*	chr5	1264513	1264712	2

© 2022 Yin L et al. *JAMA Network Open*.

434172	*TERT*	chr5	1266573	1266655	2
434159	*TERT*	chr5	1268629	1268753	1
434163	*TERT*	chr5	1271228	1271324	1
434167	*TERT*	chr5	1272294	1272400	1
434166	*TERT*	chr5	1278750	1278916	1
434160	*TERT*	chr5	1279400	1279590	1
434169	*TERT*	chr5	1280267	1280458	2
434164	*TERT*	chr5	1282538	1282744	1
434162	*TERT*	chr5	1293422	1294786	6
434158	*TERT*	chr5	1294880	1295109	0
2299745	*TNS3*	chr7	47317668	47317823	1
2299737	*TNS3*	chr7	47319756	47319793	1
2299729	*TNS3*	chr7	47319878	47319957	1
2299728	*TNS3*	chr7	47323289	47323468	1
2299739	*TNS3*	chr7	47331547	47331635	1
2299736	*TNS3*	chr7	47332420	47332508	1
2299723	*TNS3*	chr7	47333325	47333431	1
2299735	*TNS3*	chr7	47336674	47336816	1
2299726	*TNS3*	chr7	47341778	47341875	1
2299740	*TNS3*	chr7	47342542	47343187	3
2299731	*TNS3*	chr7	47344424	47344606	1
2299727	*TNS3*	chr7	47384347	47384441	1
2299744	*TNS3*	chr7	47384516	47384641	2
2299742	*TNS3*	chr7	47385779	47385959	1
2299738	*TNS3*	chr7	47407956	47409223	5
2299732	*TNS3*	chr7	47436391	47436506	1
2299748	*TNS3*	chr7	47439984	47440060	1
2299734	*TNS3*	chr7	47440376	47440516	1
2299725	*TNS3*	chr7	47451319	47451405	1
2299730	*TNS3*	chr7	47453529	47453600	1
2299743	*TNS3*	chr7	47454686	47454809	1
2299741	*TNS3*	chr7	47463693	47463787	1
2299733	*TNS3*	chr7	47467904	47467979	1
2299724	*TNS3*	chr7	47474874	47475007	1
2299747	*TNS3*	chr7	47476855	47476916	1
2299746	*TNS3*	chr7	47479079	47479239	1
423072	*TP53*	chr17	7572921	7573013	1
423062	*TP53*	chr17	7573921	7574038	1
423065	*TP53*	chr17	7576531	7576589	2
423074	*TP53*	chr17	7576619	7576662	2
423064	*TP53*	chr17	7576847	7576931	1

© 2022 Yin L et al. *JAMA Network Open*.

423068	*TP53*	chr17	7577013	7577160	2
423069	*TP53*	chr17	7577493	7577613	1
423063	*TP53*	chr17	7578171	7578294	2
1137157	*TP53*	chr17	7578365	7578457	2
423071	*TP53*	chr17	7578365	7578538	2
423066	*TP53*	chr17	7578365	7578559	2
1137158	*TP53*	chr17	7579306	7579574	2
423067	*TP53*	chr17	7579306	7579595	2
423073	*TP53*	chr17	7579694	7579726	1
423070	*TP53*	chr17	7579833	7579917	2
1126651	*TRYP2*	chr5	54320145	54320219	1
1126650	*TRYP2*	chr5	54320482	54320640	1
1126652	*TRYP2*	chr5	54326256	54326417	1
1126649	*TRYP2*	chr5	54327186	54327466	1
1126653	*TRYP2*	chr5	54329587	54329759	1
677370	*WEE1*	chr11	9595475	9596061	3
677371	*WEE1*	chr11	9597429	9597645	2
677369	*WEE1*	chr11	9597495	9597645	2
677373	*WEE1*	chr11	9597771	9597845	1
677376	*WEE1*	chr11	9598028	9598211	1
677368	*WEE1*	chr11	9598683	9598815	1
677375	*WEE1*	chr11	9603073	9603230	1
677377	*WEE1*	chr11	9606799	9606905	2
677366	*WEE1*	chr11	9606983	9607079	2
677372	*WEE1*	chr11	9607990	9608171	2
677367	*WEE1*	chr11	9608252	9608408	1
677374	*WEE1*	chr11	9609990	9610154	1
437093	*XRCC2*	chr7	152345721	152346453	3
437094	*XRCC2*	chr7	152357780	152357872	1
437092	*XRCC2*	chr7	152373120	152373169	1
518936	*XRCC3*	chr14	104165129	104165359	2
517931	*XRCC3*	chr14	104165464	104165521	1
517932	*XRCC3*	chr14	104165695	104165918	1
517926	*XRCC3*	chr14	104169504	104169669	1
517925	*XRCC3*	chr14	104173334	104173557	1
517924	*XRCC3*	chr14	104174853	104175001	1
518935	*XRCC3*	chr14	104177364	104177429	1

© 2022 Yin L et al. *JAMA Network Open*.

eTable 2. Primers for Sanger Sequencing.		
**Primer ID**	**Sequences**	**Target length**
P101-chr15-40494810-F	GCATTTACTCCTAGAGTATG	524
P101-chr15-40494810-R	GCTATCCTAGAGGATATAAAG	
P102-chr5-147207583-F	ACACAATCATTGCAACCAC	526
P102-chr5-147207583-R	ACACACAGTATCATTCTCCC	
P103-chr2-48033916-F	GCTTGTCCTAAAAGCTATG	380
P103-chr2-48033916-R	TACCACCTTTGTCAGAAGTC	
P104-chr2-48033498-F	ATTTGATGGGACGGCAATAGC	373
P104-chr2-48033498-R	TTGAATAACTTCCTCTGGG	
P105-chr13-32944538-F	GCATTAAGAACTTGTAGCAG	475
P105-chr13-32944538-R	GAGACCGAAACTCCATCTC	
P106-chr7-117120191-F	AATGACATCACAGCAGGTC	405
P106-chr7-117120191-R	TCAGTTGCAAGTAGATGTGGC	
P107-chr2-47639648-F	CATCATATCAGTGTCTTGCAC	423
P107-chr2-47639648-R	GATACACAGTTTAGGTTTTGAG	
P108-chr17-7577545-F	GAAGCCACAGGTTAAGAGGTC	337
P108-chr17-7577545-R	CATCTTGGGCCTGTGTTATC	
P109-chr2-47641557-F	AACTGGATCCAGTGGTATAG	449
P109-chr2-47641557-R	ATTGTGCCACTGCACTCTG	
P110-chr2-47641559-F	CTGGATCCAGTGGTATAG	350
P110-chr2-47641559-R	GCCATTTAAAGCTAGTTATC	
P111-chr2-47643505-F	ATGTACAGTTGAACATACGG	445
P111-chr2-47643505-R	CTCTATTACTATGTACTCTG	
P112-chr3-37048546-F	CTCATTAGAGCAAGTTACTCAG	471
P112-chr3-37048546-R	TGCTTCATTTGACAGATGAG	
P113-chr3-37053348-F	TCCATGAAGTTTCTGCTGG	477
P113-chr3-37053348-R	GACATACCGACTAACAGC	
P114-chr13-32913522-F	GAGACCATTGAGATCACAGC	583
P114-chr13-32913522-R	CTGGCTCAATACCAGAATC	
P115-chr17-41245586-F	CTTGGAAGGCTAGGATTGAC	567
P115-chr17-41245586-R	GGAGCAGAATGGTCAAGTGATG	
P116-chr11-108172425-F	CTGAATGACTAGTGAAAGTCC	477
P116-chr11-108172425-R	CACATTCCCTGGATTTATG	
P117-chr7-117267766-F	GTGAAATTGTCTGCCATTC	494
P117-chr7-117267766-R	ATAGGTTCAGGACTCTGC	
P118-chr13-32914069-F	AGATGCAAATGCATACCCAC	537
P118-chr13-32914069-R	GTATTTGCAGATGAGACTGAC	
P119-chr17-41245693-F	GAAAGTATCGCTGTCATGTC	485
P119-chr17-41245693-R	CTAACCAAACGGAGCAGAATG	
P120-chr13-32907465-F	TCAGGTCATATGACTGATCC	533

© 2022 Yin L et al. *JAMA Network Open*.

P120-chr13-32907465-R	CTACCATGTTTGAGTGACC	
P121-chr13-32954022-F	ACTACTAATGCCCACAAAGAG	629
P121-chr13-32954022-R	CAGATCACTAGTTAGCTAGC	
P122-chr3-37067242-F	ATACAGACTTTGCTACCAGG	470
P122-chr3-37067242-R	GAAGATGCAAGTGATTCATG	
P123-chr11-108186639-F	CATTTAGAGTTGGGAGTTAC	446
P123-chr11-108186639-R	CAGCACTACACTAGTGATGGC	
P124-chr3-37067157-F	TCGGGCAGAATTGCTTCTATAAC	465
P124-chr3-37067157-R	CTCTGATTTTTGGCAGCCAC	
P125-chr16-23619181-F	ACAGGCATGATCCACCATGTG	585
P125-chr16-23619181-R	CAAGCCAGTGGTTAAATCCTG	
P126-chr17-41203122-F	CATTTCAGCAATCTGAGGAAC	498
P126-chr17-41203122-R	CAAGTTAAAGCACCTGCAGAG	
P127-chr13-32907371-F	CTTGAATCTCATACAGACTGC	620
P127-chr13-32907371-R	TCATGTATACAGATGATGCC	
P128-chr2-47643481-F	CAATAGAGTCAGACCCTGTCTC	445
P128-chr2-47643481-R	ATCATGTGGGTAACTGCAGG	
P129-chr9-98002937-F	CTCAACATTAGGTCTTCATCC	541
P129-chr9-98002937-R	GAGAGCAAAGATAGAGTGAG	
P130-chr2-48030691-F	GTGCCTGGCTAACTATAGTCG	372
P130-chr2-48030691-R	CCTATTAAGTCACTGGCTG	
P131-chr17-41245927-F	CTGCTGCTTATAGGTTCAG	478
P131-chr17-41245927-R	TACTGGCCAGTGATCCTCATGAG	
P132-chr16-89866028-F	ATTCCAGTACCAGGACTCTG	447
P132-chr16-89866028-R	GGTTCACGCCATTCTCCTG	
P133-chr13-32912195-F	GACCACTTCTGAGGAATGCAG	536
P133-chr13-32912195-R	GAGAATTTCTACTGGCAGCAG	
P134-chr17-41215382-F	AGGTATGAGCCACAGTGCAG	392
P134-chr17-41215382-R	TCAACTCTGTCTCCAGAAG	
P135-chr17-33428225-F	CAATGTCTACCATCTCCTGG	506
P135-chr17-33428225-R	GTTCCAGACCTGCCATTAGG	
P136-chr9-97897639-F	GCATGCTCCAAGGATGCAG	474
P136-chr9-97897639-R	TAGCCACTGCTGTGACCATG	
P137-chr5-147204265-F	CAACAATAAGGCCAGTCAGGC	438
P137-chr5-147204265-R	GAAGGCAGAGGCATCAGGAG	
P138-chr7-117251704-F	ATTTCACAGGCAGGAGTCC	318
P138-chr7-117251704-R	GATAACCTATAGAATGCAGC	
P139-chr17-41197809-F	CTGCAGTCAGTAGTGGCTG	248
P139-chr17-41197809-R	GCTTCTACCTCATTAATCC	
P140-chr17-41246618-F	CCAAGGAACATCTTCAGTATCTC	379
P140-chr17-41246618-R	GAACACCACTGAGAAGCGTG	

© 2022 Yin L et al. *JAMA Network Open*.

P141-chr13-32907029-F	GTGAATGGTCTCAACTAACC	479
P141-chr13-32907029-R	GGAGTCCTCCTTCTGTGAG	
P142-chr17-41219625-F	CTCACGAGTAGCTGGGACTAC	432
P142-chr17-41219625-R	GAACGTGCAGGATTGCTAC	
P143-chr17-41244218-F	GGCTAGGACTCCTGCTAAG	513
P143-chr17-41244218-R	GTACAGTGAGCACAATTAG	
P144-chr7-117246728-F	GAATGCGTCTACTGTGATCC	401
P144-chr7-117246728-R	AATGTGGGATTGCCTCAGG	
P145-chr16-23614914-F	CAAGATCAGTGGTGCTACC	389
P145-chr16-23614914-R	GTATGCTATCAGGTTCCTGG	
P146-chr7-117149143-F	CAGAGCATTGAATTCTGCC	526
P146-chr7-117149143-R	GCTGAGCCCATTGAGATTG	
P147-chr2-47641545-F	ACTGGATCCAGTGGTATAG	451
P147-chr2-47641545-R	GAGATTGTGCCACTGCACTCTG	
P148-chr13-32907420-F	CAGGTCATATGACTGATCC	349
P148-chr13-32907420-R	CCTGAATCAGCATTTGCAAATG	
P149-chr13-32921033-F	GCATCTGTTACATTCACTG	446
P149-chr13-32921033-R	CTCAACCTTAGTACTTCATCC	
P150-chr16-23646660-F	CCTAATTTCACTTTGGTCAG	320
P150-chr16-23646660-R	CCAGTGACACTCTTGATG	
P151-chr13-32929248-F	CAACTAAGGAACGTCAAGAG	393
P151-chr13-32929248-R	CAGCTACTGCTTGATTGGAG	
P152-chr16-23641306-F	AGTCACAGTCACAGGTAGG	391
P152-chr16-23641306-R	GACCTTATTGTTCTACCAG	
P153-chr9-98002929-F	GTCTTCATCCTAAAGACTGG	504
P153-chr9-98002929-R	TATTCCCATCAGCAGAAAGAG	
P154-chr17-33430317-F	TATGGTGCAAAGCTGTAGCTG	442
P154-chr17-33430317-R	AGGTGACTGGTTCTTCAGG	
P155-chr13-32912337-F	CGGAAGTTTGCTGGCCTGTTG	425
P155-chr13-32912337-R	GAATTTCTACTGGCAGCAG	
P156-chr17-41246508-F	CATCACTTCTGGAAAACCACTC	376
P156-chr17-41246508-R	GTTTCAAACTTGCATGTGGAG	
P157-chr7-117175335-F	GGAAAACCGATTCTATGTG	474
P157-chr7-117175335-R	GACACTGAAGATCACTGTTC	
P158-chr7-117232266-F	CCAAAATCTACAGCCAGAC	419
P158-chr7-117232266-R	TCAGGTTCAGGACAGACTG	
P159-chr11-108216476-F	CCAGACTGTTAGCTTCTTG	443
P159-chr11-108216476-R	GATGGGTTGGTTACTAACCAC	
P160-chr17-41215387-F	GCATTGTTAAGGAAAGTGG	385
P160-chr17-41215387-R	TAATAGTCGGCAGGAATCC	
P161-chr13-32913422-F	GACCATTGAGATCACAGCTG	508

© 2022 Yin L et al. *JAMA Network Open*.

P161-chr13-32913422-R	GGAATAGCTGTTAGACATGC	
P162-chr17-41245390-F	CGAGATACTTTCCTGAGTGCC	390
P162-chr17-41245390-R	TACAACCAAATGCCAGTCAGG	
P163-chr13-32900280-F	GATTACAGGCGTGAACCACTG	583
P163-chr13-32900280-R	TATGAGGCAGAATGCTAGG	
P164-chr16-68835596-F	AATCAGAGCACAAGGAAGTC	371
P164-chr16-68835596-R	GGTGGAAAACTTTCTGTAGG	
P165-chr13-32914102-F	GAGGAACTTGTGACTAGCTC	529
P165-chr13-32914102-R	GGACAGATTTTCCACTTGCTGTGC	
P166-chr13-32914790-F	GTACAGCAAGTGGAAAGCAAG	507
P166-chr13-32914790-R	CTGCTTCTGTTTCAAAGTAG	
P167-chr11-108175459-F	TTACTCAAACTATTGGGTGG	446
P167-chr11-108175459-R	CTGAGGGAATACTATACATC	
P168-chr13-32914522-F	CATAAGTCAGTCTCATCTGC	384
P168-chr13-32914522-R	GTGAAGACTATGCTCAGTTCTG	
P169-chr13-32913422-F	CAGAGAGGCCTGTAAAGACC	349
P169-chr13-32913422-R	TGAAGTCTGACTCACAGAAG	
P170-chr17-33434458-F	GGCTATGCATCTACCACCC	369
P170-chr17-33434458-R	CCTCTCTGGAGAGCTGATCT	
P171-chr16-23641218-F	GTGAATGACTCAATGGGTGG	432
P171-chr16-23641218-R	GGACCTTATTGTTCTACCAG	
P172-chr13-32911073-F	GTTCTGTCAAACCTAGTCATG	424
P172-chr13-32911073-R	CGTTTACACAAGTCAAGTCTG	
P173-chr16-23635403-F	CTTTCAAGACTCAAGCCTAG	529
P173-chr16-23635403-R	GCTTTACACAGAGGTGCCCA	
P174-chr19-1221947-F	TGTCAGGGTTGTCCTGCTGCAC	618
P174-chr19-1221947-R	ACGGACGCATGTCCTCACTC	
P175-chr7-117254665-F	GTGATATGTGCCCTAGGAG	388
P175-chr7-117254665-R	CTTCATAGTGGCTATCTATG	
P176-chr2-47698134-F	TTGTGCCATTGCGCTATTC	499
P176-chr2-47698134-R	TCCAGTTATGTGTCCTACTG	
P177-chr17-41244821-F	AGTGGTGGTATACGATATG	459
P177-chr17-41244821-R	GTATCCATTGGGACATGAAG	
P178-chr7-117232086-F	CTGTGTCTGTAAACTGATGGC	397
P178-chr7-117232086-R	CATTTGTAAGGGAGTCTTTTGC	
P179-chr17-59878688-F	CACTTCAGTCACTATGTTCC	546
P179-chr17-59878688-R	GTGGCTTTAATGATGTTCC	
P180-chr7-117180229-F	GGTCACACAGGTCATATGATG	599
P180-chr7-117180229-R	CCACTCTCATCCATCATACTG	
P181-chr17-41244280-F	TCATCTAACAGGTCATCAGG	539
P181-chr17-41244280-R	CCATATCGTATACCACCAC	

© 2022 Yin L et al. *JAMA Network Open*.

P182-chr2-47657081-F	GATGCAGAATTGAGGCAGAC	272
P182-chr2-47657081-R	CTAAGAGTGAGTCACCACCAC	
P183-chr13-32954272-F	GTATCAACAACTACCGGTAC	377
P183-chr13-32954272-R	GGCTTACTTTCAGATCACTAG	
P1-chr2-47643505-F	CTCCAGTCTGGGCAATAGAG	457
P1-chr2-47643505-R	ATCATGTGGGTAACTGCAGG	
P2-chr3-37067240-F	TTGCTACCAGGACTTGCTGGC	300
P2-chr3-37067240-R	CAAGCTCTGATTTTTGGCAGC	
P3-chr13-32912345-F	AGTGAGGAAACTTCTGCAGAG	343
P3-chr13-32912345-R	GCTGATCAGTAAATAGCAAGTCC	
P4-chr16-23614921-F	CTAAGAGGCCCAATATATCC	272
P4-chr16-23614921-R	GTATGCTATCAGGTTCCTGG	
P5-chr13-32910922-F	ATCATGAAAATGCCAGCACTC	356
P5-chr13-32910922-R	TTCAGAGTCTGGATTGACAG	
P6-chr5-147207583-F	CTTTTCTCGGGGTGAGATTC	387
P6-chr5-147207583-R	GAAATAGCAGAGGCATGAC	
P7-chr7-117188684-F	AGATCATGTCCTCTAGAAACCG	424
P7-chr7-117188684-R	GACATGGACACCAAATTAAG	
P8-chr7-142459789-F	CATGAGCAGAGAGCTTGAGGAAC	550
P8-chr7-142459789-R	GTTCTCCATTTGTCCTGTC	
P9-chr3-37053348-F	TCCATGAAGTTTCTGCTGG	467
P9-chr3-37053348-R	CTAACAGCATTTCCAAAGATGG	
P10-chr15-40494810-F	AAGTGAGGATAAATTAGGG	409
P10-chr15-40494810-R	CACAATTTTGCCAACACTTG	
P11-chr2-47641557-F	TAGGTTGCAGTTTCATCACTG	446
P11-chr2-47641557-R	CTGTAATCCCAACTACTTGG	
P12-chr2-47641559-F	TGCAGTTTCATCACTGTCTG	289
P12-chr2-47641559-R	GCTAGTTATCTAATCCAAG	
P13-chr2-48033498-F	GGTACTGCAACATTTGATGG	430
P13-chr2-48033498-R	GTGACTGATTCATCTTCTC	
P14-chr2-47639648-F	CAGTACATCATATCAGTGTC	432
P14-chr2-47639648-R	CATTGATACACAGTTTAGG	
P15-chr2-47698134-F	CTACATTTGTCCCTAAGGAG	438
P15-chr2-47698134-R	GTTATGTGTCCTACTGTAAC	
P16-chr7-117232470-F	CAATCCAATCAACTCTATACG	390
P16-chr7-117232470-R	CCTACCTTTAAGTCTTCTTCG	
P17-chr11-108216476-F	GTAGGTAATGTATCCTGTTC	323
P17-chr11-108216476-R	GTGCTCAATCTACTATATG	
P18-chr17-41215382-F	AGTGGTGCATTGATGGAAGG	367
P18-chr17-41215382-R	AGTCGGCAGGAATCCATGTG	
P19-chr2-47641545-F	CCAGTGGTATAGAAATCTTCG	290

© 2022 Yin L et al. *JAMA Network Open*.

P19-chr2-47641545-R	GCTCCTTTATAAGCTTCTTCAG	
P20-chr7-117232266-F	CTACAGCCAGACTTTAGCTC	422
P20-chr7-117232266-R	GAGTGTGTCATCAGGTTCAG	
P21-chr17-41246508-F	GTCATCAGAACCTAACAG	384
P21-chr17-41246508-R	CATGTGGAGCCATGTGGCA	
P22-chr17-41243677-F	CAATTCCTTGTCACTCAGAC	434
P22-chr17-41243677-R	CACATTTGGCTCAGGGTTAC	
P23-chr11-108121593-F	AGGCTACAGATTGCAACCC	413
P23-chr11-108121593-R	GTTTATCTGTAAGTCAGAC	
P24-chr3-37067242-F	ATACAGACTTTGCTACCAGG	447
P24-chr3-37067242-R	AGCCAAAGTTAGAAGGCAG	
P25-chr17-41215387-F	AGTGCAGGCCTGCATAATTC	532
P25-chr17-41215387-R	AGATAAGCTGGTGATGCTGG	
P26-chr17-41203122-F	GCCTCTAGAACATTTCAGC	350
P26-chr17-41203122-R	GACATTGGACTGCTTGTCC	
P27-chr11-108160486-F	CTTGCCATATGTGAGCAAGC	394
P27-chr11-108160486-R	GCATGGTGGTATGCTCCTG	
P28-chr17-7577121-F	ATCTGAGGCATAACTGCACC	400
P28-chr17-7577121-R	CTCCTTACTGCTCCCACTCAG	
P29-chr3-37059048-F	GGGATGTGATGTGCATATCAC	513
P29-chr3-37059048-R	CGCCTACAAGCTATCAGCAC	
P30-chr15-40512842-F	GTTCTAGGTATTACCTTACACC	501
P30-chr15-40512842-R	GGCCATGAGTAAGTATATC	
P31-chr19-1220628-F	CCTCAAAATCTCCGACCTG	415
P31-chr19-1220628-R	CCATCTGCCGTATGAGTTAC	
P32-chr2-48030691-F	CCTATAAAACACTTAGGCTG	474
P32-chr2-48030691-R	CAGGGAGTAATTTCCCTTTG	
P33-chr7-117267766-F	GACAAATAGCAAGTGTTGC	593
P33-chr7-117267766-R	GATATTCTGCAAGTACAATC	
P34-chr2-48027625-F	TGGTTCTACTGAAGGAACC	582
P34-chr2-48027625-R	CTTATGTCAGCAAGAGCTTGG	
P35-chr13-32968945-F	TGCTTCCACATGACCTCAG	597
P35-chr13-32968945-R	CTTTACCTCACATACTACCTC	
P36-chr17-41234420-F	ACCATGTATCTTCCATGGGCTC	550
P36-chr17-41234420-R	CTTGTAGTTCCATACTAGGTG	
P37-chr7-117246728-F	GAGTACCCACCTATTCCTGAC	589
P37-chr7-117246728-R	TTGGGCCAGGTAAGCAGTTCTG	
P38-chr7-117120191-F	GTGGAGAAAGCCGCTAGAGC	581
P38-chr7-117120191-R	GTGCATAGTAGCGTACTTGAG	
P39-chr13-32914790-F	GAAGATAGTACCAAGCAAGTC	523
P39-chr13-32914790-R	GAATGTTCTCAACAAGTGAC	

© 2022 Yin L et al. *JAMA Network Open*.

P40-chr13-32907420-F	GGCAATATCTGGAACTTCTCC	453
P40-chr13-32907420-R	CCTGAATCAGCATTTGCAAATG	
P41-chr2-47635538-F	AACGGAAGTCTACCTGCAC	384
P41-chr2-47635538-R	ACCAATCATTCTCCTTGGATGC	
P42-chr17-41245724-F	CTCTTCACTGCTAGAACAAC	422
P42-chr17-41245724-R	CATCAGGCCTTCATCCTGAG	
P43-chr17-7574003-F	GTGGTTATAGGATTCAACCG	530
P43-chr17-7574003-R	CAGAGGCGGAGATTGCAATCAG	
P44-chr7-117180229-F	TGCCCAAGGTCACACAGGTC	420
P44-chr7-117180229-R	GCAGCATTATGGTACATTACC	
P45-chr3-37083822-F	CTCTAACCTGTCTGTTAGAG	591
P45-chr3-37083822-R	TCCCAAAGTGGTGGGATTAC	
P46-chr17-41245873-F	CTGACTACTAGTTCAAGCGC	417
P46-chr17-41245873-R	GCCACAGATAATACAAGAGC	
P47-chr7-117149143-F	ATCTGGCTGAGTGTTTGGTG	503
P47-chr7-117149143-R	GCTGAGCCCATTGAGATTG	
P48-chr13-32890627-F	ACTAGCCACGTTTCGAGTGC	414
P48-chr13-32890627-R	GCAACACTGTGACGTACTGG	
P49-chr17-41223093-F	AAGTCTTAGTCATTAGGGAG	368
P49-chr17-41223093-R	TCTCCCATTCCTTTCAGAGG	
P50-chr17-41245759-F	ACTTGTCTGTTCATTTGGC	620
P50-chr17-41245759-R	TAGGAGCATTTGTTACTGAGCC	
P51-chr17-41246618-F	CCATCATGTGAGTCATCAGAAC	465
P51-chr17-41246618-R	GAACACCACTGAGAAGCGTG	
P52-chr17-41245586-F	GCCATAATCAGTACCAGGTACC	572
P52-chr17-41245586-R	GCAGCAGTATAAGCAATATGG	
P53-chr7-117175335-F	GCACATTGCTATGTGCTCC	445
P53-chr7-117175335-R	ATGACACTGAAGATCACTG	
P54-chr11-108151842-F	TAAGTCCCATAGTGCTGAGAACC	373
P54-chr11-108151842-R	TGTAAGAAACAGTGCATACC	
P55-chr3-37067157-F	CGGGCAGAATTGCTTCTATAAC	394
P55-chr3-37067157-R	TGCCACTAGAAATATCTGTC	
P56-chr13-32913456-F	CCTTGAATTAGCATGTGAGACC	521
P56-chr13-32913456-R	AGCTGTTAGACATGCTACTG	
P57-chr11-108151896-F	AAGTCCCATAGTGCTGAGAACC	435
P57-chr11-108151896-R	GGTGGACAAGTCATTCACTC	
P58-chr17-41215392-F	AGCGCTGGGATTATAGGTATG	512
P58-chr17-41215392-R	GGACCTAATCTGCTCCTAGC	
P59-chr9-98002937-F	CAAAACCACTCAACATTAGGTC	500
P59-chr9-98002937-R	TGTGCAAAGTATGATGGCGAG	
P60-chr16-23634452-F	CCTAGTTACCCAACTTTCTC	473

© 2022 Yin L et al. *JAMA Network Open*.

P60-chr16-23634452-R	GTGGTGGTGCGCACCTATAATC	
P61-chr13-32911073-F	CCTACTTCCAAGGATGTTCT	514
P61-chr13-32911073-R	TAATTGACACTTGGGTTGC	
P62-chr16-23625413-F	CCAAGCAATCACTGTACCAAC	568
P62-chr16-23625413-R	GTGCCTGATTTCAATACCAG	
P63-chr19-1218414-F	CACAGCACTGTGAACTCACAG	396
P63-chr19-1218414-R	CAGCCATTGCCACAATGGCTG	
P64-chr13-32929248-F	CTAAGGAACGTCAAGAGATAC	400
P64-chr13-32929248-R	GTGAAAGTTACAGCTACTGC	
P65-chr13-32914522-F	GAATGTAGCACGCATTCAC	464
P65-chr13-32914522-R	ACTTGCTTTCCACTTGCTG	
P66-chr3-37081675-F	CTCTGAGTTCCTGCTTTGTC	491
P66-chr3-37081675-R	ACCACATGTGTCTGACTCC	
P67-chr11-108224553-F	GTTGGGTTAAGAAGTAGTTAGG	499
P67-chr11-108224553-R	GTCTTGCTGTATTGCCTAG	
P68-chr13-32906565-F	CTATGAGAAAGGTTGTGAG	380
P68-chr13-32906565-R	GGATCAGTATCATTTGGTTC	
P69-chr16-89809210-F	CTTGCTCCAAGCCACATATTTG	406
P69-chr16-89809210-R	CTAGTTTCTGATGGTCCTG	
P70-chr17-41215950-F	CTGCTGCACATGGATTCCTG	517
P70-chr17-41215950-R	CAGCCTCTGATTCTGTCAC	
P71-chr11-108213967-F	CTCCTAACTGGACAAACTAAG	533
P71-chr11-108213967-R	GCTGTCAGCTTTAATAAGCC	
P72-chr17-41234508-F	GTAGGTACTCAGATGACAAC	509
P72-chr17-41234508-R	CTTGTAGTTCCATACTAGG	
P73-chr17-41199722-F	TGCCAAGAACTGTGCTACTC	457
P73-chr17-41199722-R	CAGGAGTTTGAGACCAGCCTGG	
P74-chr17-41219625-F	AGTAGAGACGGGGTTTCAC	389
P74-chr17-41219625-R	AGTTCCAGGACACGTGTAG	
P75-chr13-32954022-F	TCACTTCTTCCATTGCATC	416
P75-chr13-32954022-R	CGACAAATCCTATTAGGTCC	
P76-chr1-27106621-F	CTTTGAGATGTCCAAACACC	422
P76-chr1-27106621-R	GTGTGGCCAGAATCAGGTCCAC	
P77-chr17-41243479-F	GGATGTTAAAGCTCATTCAGTC	476
P77-chr17-41243479-R	GGCATCTCAGGAACATCAC	
P78-chr17-59885995-F	CTAGCAATTCCATGCACTTC	350
P78-chr17-59885995-R	CCATGTGAGGTTTGATAACG	
P79-chr3-37081767-F	ACCAGGAGGCTCAATTCAGGC	490
P79-chr3-37081767-R	ACCCCTGCATGTTAACTAG	
P80-chr17-41245583-F	CTTGGAAGGCTAGGATTGAC	428
P80-chr17-41245583-R	CCTATAAGCAGCAGTATAAGC	

© 2022 Yin L et al. *JAMA Network Open*.

P81-chr17-41222943-F	GGTAAATTCACCCATGTGAG	575
P81-chr17-41222943-R	CCATCTTCAACCTCTGCATTG	
P82-chr3-37053500-F	TTCAGTACACAATGCAGGC	435
P82-chr3-37053500-R	CTCCATTAACAAATCTGAAGC	
P83-chr16-89833576-F	AAGGCAGCACTCAGCAGTC	439
P83-chr16-89833576-R	AGGACCAATGCTCAGGCCATC	
P84-chr17-59878688-F	ATCCCACTTCAGTCACTATG	486
P84-chr17-59878688-R	GGTTCACATTTCAGTTATGC	
P85-chr16-89831344-F	CCATCTAAGTGCTGCTGTTC	462
P85-chr16-89831344-R	TTGCTCAGCCACTCACAGTG	
P86-chr7-117175301-F	CCGATTCTATGTGTAGAATG	449
P86-chr7-117175301-R	CTATGCATAGAGCAGTCCTGG	
P87-chr19-1221319-F	TAGCCTCCACTAGTGGAAGGTG	360
P87-chr19-1221319-R	CAACCCTACATTTCTGCAC	
P201-chr13-32911228-F	TCCCATGGAAAAGAATCAAG	500
P201-chr13-32911228-R	CTATATTCAAGGAGATGTCC	
P202-chr13-32906915-F	GATACTGATCCATTAGATTC	547
P202-chr13-32906915-R	CCAGTCCACTTTCAGAGGCTTC	
P203-chr17-33445587-F	TGAGATACCCCATAACCCTG	480
P203-chr17-33445587-R	TGCTACTGCACTCCAATCTG	

© 2022 Yin L et al. *JAMA Network Open*.

**eTable 3. Prevalence of pathogenic germline mutations in PDAC and Chinese population.**									
**Disease**	**Gene**	**Chromosome Position**	**Amino Acid Change**	**Nucleotide Change**	**Function**	**Gene group**	**Nanjing Allele Count (%)**	**ChinaMap Allele Count (%)**	***P* value**
PDAC	*ATM*	chr11:108153468	p.Tyr1203Ter	c.3609delT	Nonsense	Pancreatic cancer susceptibility genes	1/2,018 (0.05)	0/21,176 (0)	0.09
PDAC	*ATM*	chr11:108170440	Splice	c.5006-1G>A	Noncoding	Pancreatic cancer susceptibility genes	1/2,018 (0.05)	0/21,176 (0)	0.09
PDAC	*ATM*	chr11:108178646	p.Cys1899Ter	c.5697C>A	Nonsense	Pancreatic cancer susceptibility genes	1/2,018 (0.05)	2/21,176 (0.009)	0.61
PDAC	*ATM*	chr11:108186639	Splice	c.6095+1G>A	Noncoding	Pancreatic cancer susceptibility genes	1/2,018 (0.05)	0/21,176 (0)	0.09
PDAC	*BRCA1*	chr17:41197809	p.Ile1845fs	c.5533_5540delATTGGGCA	Frameshift	Pancreatic cancer susceptibility genes	1/2,018 (0.05)	4/21,176 (0.019)	0.92
PDAC	*BRCA1*	chr17:41219625	p.Asp1713Asn	c.5137G>A	Missense	Pancreatic cancer susceptibility genes	1/2,018 (0.05)	0/21,176 (0)	0.09
PDAC	*BRCA1*	chr17:41245390	p.Glu720fs	c.2157_2158insA	Frameshift	Pancreatic cancer susceptibility genes	1/2,018 (0.05)	0/21,176 (0)	0.09
PDAC	*BRCA2*	chr13:32900280	p.Lys157fs	c.470_474delAGTCA	Frameshift	Pancreatic cancer susceptibility genes	1/2,018 (0.05)	0/21,176 (0)	0.09
PDAC	*BRCA2*	chr13:32906915	p.Lys437fs	c.1310_1313delAAGA	Frameshift	Pancreatic cancer susceptibility genes	1/2,018 (0.05)	0/21,176 (0)	0.09
PDAC	*BRCA2*	chr13:32907029	p.Gln472Ter	c.1414C>T	Nonsense	Pancreatic cancer susceptibility genes	1/2,018 (0.05)	0/21,176 (0)	0.09
PDAC	*BRCA2*	chr13:32907371	p.Lys586Ter	c.1756A>T	Nonsense	Pancreatic cancer susceptibility genes	1/2,018 (0.05)	0/21,176 (0)	0.09
PDAC	*BRCA2*	chr13:32911228	p.Thr915fs	c.2743_2747delACTTG	Frameshift	Pancreatic cancer susceptibility genes	1/2,018 (0.05)	0/21,176 (0)	0.09
PDAC	*BRCA2*	chr13:32912337	p.Val1283fs	c.3847_3848delGT	Frameshift	Pancreatic cancer susceptibility genes	1/2,018 (0.05)	0/21,176 (0)	0.09
PDAC	*BRCA2*	chr13:32914102	p.Lys1872fs	c.5616_5620delAGTAA	Frameshift	Pancreatic cancer susceptibility genes	1/2,018 (0.05)	0/21,176 (0)	0.09
PDAC	*BRCA2*	chr13:32921033	p.Arg2336His	c.7007G>A	Missense	Pancreatic cancer susceptibility genes	1/2,018 (0.05)	0/21,176 (0)	0.09
PDAC	*BRCA2*	chr13:32944538	Splice	c.8332-1G>T	Noncoding	Pancreatic cancer susceptibility genes	1/2,018 (0.05)	0/21,176 (0)	0.09
PDAC	*BRIP1*	chr17:59858254	p.Arg581Ter	c.1741C>T	Nonsense	Candidate pancreatic cancer susceptibility genes	2/2,018 (0.10)	0/21,176 (0)	0.01
PDAC	*BRIP1*	chr17:59878688	p.Arg356Ter	c.1066C>T	Nonsense	Candidate pancreatic cancer susceptibility genes	1/2,018 (0.05)	0/21,176 (0)	0.09
PDAC	*CFTR*	chr7:117149143	p.Arg74Trp	c.220C>T	Missense	Pancreatitis associated genes	2/2,018 (0.10)	20/21,176 (0.094)	0.75
PDAC	*CFTR*	chr7:117232086	p.Gly622Asp	c.1865G>A	Missense	Pancreatitis associated genes	1/2,018 (0.05)	2/21,176 (0.009)	0.61
PDAC	*CFTR*	chr7:117246728	p.Gly970Asp	c.2909G>A	Missense	Pancreatitis associated genes	3/2,018 (0.15)	15/21,176 (0.071)	0.43

© 2022 Yin L et al. *JAMA Network Open*.

PDAC	*CFTR*	chr7:117251704	p.Arg1070Gln	c.3209G>A	Missense	Pancreatitis associated genes	1/2,018 (0.05)	19/21,176 (0.090)	0.85
PDAC	*CFTR*	chr7:117254665	Splice	c.3368-2A>G	Noncoding	Pancreatitis associated genes	1/2,018 (0.05)	0/21,176 (0)	0.09
PDAC	*FANCC*	chr9:98002929	Splice	c.345+2GT>T	Noncoding	Candidate pancreatic cancer susceptibility genes	1/2,018 (0.05)	0/21,176 (0)	0.09
PDAC	*FANCC*	chr9:98002937	p.Trp113Ter	c.339G>A	Nonsense	Candidate pancreatic cancer susceptibility genes	1/2,018 (0.05)	3/21,176 (0.014)	0.79
PDAC	*PALB2*	chr16:23614914	p.Leu1143fs	c.3426_3427insA	Frameshift	Pancreatic cancer susceptibility genes	1/2,018 (0.05)	0/21,176 (0)	0.09
PDAC	*PALB2*	chr16:23619181	Splice	c.3350+4A>G	Noncoding	Pancreatic cancer susceptibility genes	1/2,018 (0.05)	0/21,176 (0)	0.09
PDAC	*PALB2*	chr16:23635403	p.Gln921fs	c.2760_2761insA	Frameshift	Pancreatic cancer susceptibility genes	1/2,018 (0.05)	1/21,176 (0.005)	0.41
PDAC	*PALB2*	chr16:23641218	p.Arg753Ter	c.2257C>T	Nonsense	Pancreatic cancer susceptibility genes	1/2,018 (0.05)	3/21,176 (0.014)	0.79
PDAC	*PALB2*	chr16:23646660	p.Leu403fs	c.1206delT	Frameshift	Pancreatic cancer susceptibility genes	1/2,018 (0.05)	0/21,176 (0)	0.09
PDAC	*RAD51D*	chr17:33428225	p.Arg320Ter	c.958C>T	Nonsense	Known cancer susceptibility genes	1/2,018 (0.05)	4/21,176 (0.019)	0.92
PDAC	*RAD51D*	chr17:33430317	p.Arg252Ter	c.754C>T	Nonsense	Known cancer susceptibility genes	1/2,018 (0.05)	0/21,176 (0)	0.09
PDAC	*RAD51D*	chr17:33434458	p.Lys111fs	c.331_332insTA	Frameshift	Known cancer susceptibility genes	1/2,018 (0.05)	12/21,176 (0.057)	0.72
PDAC	*SPINK1*	chr5:147207583	Splice	c.194+2T>C	Noncoding	Pancreatitis associated genes	21/2,018 (1.04)	114/21,176 (0.538)	0.01
PDAC	*TP53*	chr17:7577545	p.Met246Val	c.736A>G	Missense	Pancreatic cancer susceptibility genes	1/2,018 (0.05)	0/21,176 (0)	0.09
PDAC	*XRCC2*	chr7:152346380	p.Arg64Ter	c.190C>T	Nonsense	Candidate pancreatic cancer susceptibility genes	1/2,018 (0.05)	0/21,176 (0)	0.09

© 2022 Yin L et al. *JAMA Network Open*.

eTable 4. Variants of uncertain significance and novel variants detected.

Case ID	Gender	Age	Race	Disease	Gene	Amino Acid Change	Chromosome Position	Nucleotide Change	Function	Clinvar Classification	Personal History of Other Disease	Family History of Cancer
P0105	Female	52	Asian	Ampullary cancer	*ATM*	p.Ala2324fs	chr11:108196949	c.6973_6974insTCG	Frameshift	Novel	Negative	Negative
P0107	Male	78	Asian	PDAC	*RAD51B*	p.Leu172Trp	chr14:68352648	c.515T>G	Missense	Novel	Negative	Negative
P0107	Male	78	Asian	PDAC	*MSH2*	p.Pro618Leu	chr2:47702257	c.1853C>T	Missense	VUS	Negative	Negative
P0110	Female	73	Asian	PDAC	*ATM*	p.Leu1608Ser	chr11:108165700	c.4823T>C	Missense	Novel	Negative	Negative
P0110	Female	73	Asian	PDAC	*ATM*	p.Ala1634Gly	chr11:108165778	c.4901C>G	Missense	Novel	Negative	Negative
P0114	Male	63	Asian	PDAC	*CPA1*	p.Gln326Ter	chr7:130025175	c.976C>T	Nonsense	Novel	Negative	Negative
P0202	Male	59	Asian	PDAC	*MSH2*	p.Pro5Gln	chr2:47630344	c.14C>A	Missense	VUS	Negative	Negative
P0205	Male	76	Asian	PDAC	*CDH1*	p.Thr522Ile	chr16:68849662	c.1565C>T	Missense	VUS	Negative	Negative
P0207	Male	62	Asian	PDAC	*BRCA1*	p.Glu1017Gly	chr17:41244498	c.3050A>G	Missense	Novel	Negative	Negative
P0208	Female	65	Asian	PDAC	*MSH6*	p.Tyr1066Cys	chr2:48030583	c.3197A>G	Missense	VUS	Negative	Negative
P0213	Female	46	Asian	PDAC	*CELA2A*	p.Asn244Ser	chr1:15793972	c.731A>G	Missense	Novel	Negative	Negative
P0213	Female	46	Asian	PDAC	*PALB2*	p.Leu9Phe	chr16:23652454	c.25C>T	Missense	VUS	Negative	Negative
P0229	Male	75	Asian	PDAC	*ATM*	p.Ala3054Thr	chr11:108236224	c.9160G>A	Missense	VUS	Negative	Negative
P0235	Male	69	Asian	PDAC	*FANCA*	p.Pro799Ser	chr16:89836354	c.2395C>T	Missense	VUS	Negative	Negative
P0238	Male	65	Asian	PDAC	*ERCC4*	p.Ile266Met	chr16:14024572	c.798C>G	Missense	VUS	Negative	Negative
P0240	Male	56	Asian	PDAC	*MSH2*	p.Thr31Asn	chr2:47630422	c.92C>A	Missense	Novel	Negative	Negative
P0240	Male	56	Asian	PDAC	*FANCL*	p.Ser240Ala	chr2:58390641	c.718T>G	Missense	Novel	Negative	Negative
P0244	Male	79	Asian	PDAC	*BRIP1*	p.Leu881Ser	chr17:59763460	c.2642T>C	Missense	Novel	Negative	Negative
P0245	Male	53	Asian	PDAC	*CFTR*	p.Lys688Ter	chr7:117232278	c.2057_2058insA	Nonsense	Novel	Negative	Negative
P0247	Male	69	Asian	PDAC	*PALB2*	p.Thr710Met	chr16:23641346	c.2129C>T	Missense	VUS	Negative	Negative
P0249	Male	67	Asian	PDAC	*BRCA1*	p.Ile917Thr	chr17:41244798	c.2750T>C	Missense	VUS	Negative	Negative
P0251	Female	65	Asian	PDAC	*CLPS*	p.Arg22fs	chr6:35765000	c.64_65delCG	Frameshift	Novel	Negative	Negative
P0252	Male	73	Asian	PDAC	*RECQL4*	p.Arg618Gln	chr8:145739598	c.1853G>A	Missense	VUS	Negative	Negative
P0261	Female	56	Asian	PDAC	*MSH6*	p.Gly1069Arg	chr2:48030591	c.3205G>C	Missense	VUS	Breast cancer	Negative
P0261	Female	56	Asian	PDAC	*TNS3*	p.Gly1120Asp	chr7:47342646	c.3359G>A	Missense	Novel	Breast cancer	Negative
P0267	Female	60	Asian	PDAC	*FANCA*	p.Ser53Arg	chr16:89882317	c.157A>C	Missense	VUS	Negative	Negative
P0274	Female	76	Asian	PDAC	*BRCA2*	p.Phe2457Leu	chr13:32929361	c.7371T>A	Missense	Novel	Negative	Negative

© 2022 Yin L et al. *JAMA Network Open*.

P0275	Male	61	Asian	PDAC	*BRIP1*	p.Ala745Thr	chr17:59821817	c.2233G>A	Missense	VUS	Negative	Negative
P0278	Male	68	Asian	PDAC	*TERT*	p.Ser656Trp	chr5:1279569	c.1967C>G	Missense	Novel	Negative	Negative
P0288	Male	58	Asian	PDAC	*WEE1*	p.Ala281fs	chr11:9597835	c.841_842insA	Frameshift	Novel	Negative	Negative
P0301	Male	59	Asian	PDAC	*CTRB1*	p.Trp225Gly	chr16:75258645	c.673T>G	Missense	Novel	Negative	Negative
P0308	Female	64	Asian	PDAC	*CFTR*	p.Ile1131Thr	chr7:117254691	c.3392T>C	Missense	Novel	Esophagus cancer	Negative
P0312	Female	63	Asian	PDAC	*CEL*	p.Pro323Leu	chr9:135944122	c.968C>T	Missense	Novel	Negative	Negative
P0318	Male	61	Asian	PDAC	*RAD51D*	p.Gln18Arg	chr17:33446580	c.53A>G	Missense	VUS	Negative	Negative
P0330	Female	52	Asian	PDAC	*BUB1*	p.His163fs	chr2:111427100	c.487_496delCAT AATGTTC	Frameshift	Novel	Negative	Negative
P0330	Female	52	Asian	PDAC	*BUB1*	p.Ser159Ter	chr2:111427121	c.476C>G	Nonsense	Novel	Negative	Negative
P0332	Male	53	Asian	PDAC	*RNF43*	p.Arg296His	chr17:56437575	c.887G>A	Missense	Novel	Negative	Negative
P0347	Male	64	Asian	PDAC	*MSH6*	p.Val1056Leu	chr2:48028288	c.3166G>C	Missense	VUS	Colon polyp	Father with colon cancer
P0354	Female	49	Asian	PDAC	*BAP1*	p.Arg383Cys	chr3:52438572	c.1147C>T	Missense	VUS	Negative	Negative
P0356	Female	78	Asian	PDAC	*BRCA2*	p.Ile3169Thr	chr13:32971039	c.9506T>C	Missense	VUS	Negative	Negative
P0379	Male	61	Asian	PDAC	*ATM*	p.Glu699Gln	chr11:108124737	c.2095G>C	Missense	VUS	Negative	Negative
P0379	Male	61	Asian	PDAC	*ATM*	p.Arg1086Cys	chr11:108143551	c.3256C>T	Missense	VUS	Negative	Negative
P0410	Male	58	Asian	PDAC	*GRP*	p.Arg141fs	chr18:56897667	c.422_425delGG AA	Frameshift	Novel	Negative	Negative
P0415	Male	58	Asian	PDAC	*BRIP1*	p.Asn643Ile	chr17:59857629	c.1928A>T	Missense	Novel	Negative	Negative
P0418	Female	74	Asian	PDAC	*PMS2*	p.Pro574Arg	chr7:6026675	c.1721C>G	Missense	Novel	Lung cancer	Negative
P0418	Female	74	Asian	PDAC	*FANCC*	p.Arg535Cys	chr9:97864063	c.1603C>T	Missense	VUS	Lung cancer	Negative
P0419	Male	48	Asian	PDAC	*PNLIPRP 2*	p.Asn259fs	chr10:118390822	c.778_779insA	Frameshift	Novel	Negative	Negative
P0421	Female	68	Asian	PDAC	*TNS3*	p.Lys147fs	chr7:47463734	c.437_438insT	Frameshift	Novel	Negative	Negative
P0425	Male	54	Asian	PDAC	*TP53*	p.Val157Ile	chr17:7578461	c.469G>A	Missense	VUS	Negative	Negative
P0426	Male	71	Asian	PDAC	*TP53*	p.Ala86Val	chr17:7579430	c.257C>T	Missense	VUS	Negative	Negative
P0431	Female	67	Asian	PDAC	*BRCA1*	p.Gly813Val	chr17:41245110	c.2438G>T	Missense	Novel	Negative	Negative
P0434	Male	61	Asian	PDAC	*BRCA2*	p.Asp2438Asn	chr13:32929302	c.7312G>A	Missense	Novel	Negative	Negative
P0434	Male	61	Asian	PDAC	*MSH6*	p.Asn345Ser	chr2:48026156	c.1034A>G	Missense	VUS	Negative	Negative
P0438	Female	69	Asian	PDAC	*ATM*	p.Ile326Thr	chr11:108117766	c.977T>C	Missense	VUS	Negative	Negative
P0448	Female	66	Asian	PDAC	*PLA2G1B*	p.Asn110fs	chr12:120760113	c.329delA	Frameshift	Novel	Negative	Negative
P0450	Male	47	Asian	PDAC	*FANCG*	p.Glu134Val	chr9:35078247	c.401A>T	Missense	Novel	Negative	Negative
P0457	Male	68	Asian	PDAC	*PNLIP*	p.Glu196fs	chr10:118314702	c.584_585insG	Frameshift	Novel	Negative	Negative

© 2022 Yin L et al. *JAMA Network Open*.

P0457	Male	68	Asian	PDAC	*BUB1B*	p.Glu418Lys	chr15:40488939	c.1252G>A	Missense	Novel	Negative	Negative
P0471	Male	79	Asian	PDAC	*BRCA1*	p.Thr249Ala	chr17:41246803	c.745A>G	Missense	Novel	Negative	Negative
P0473	Male	62	Asian	PDAC	*FANCA*	p.Leu784fs	chr16:89836399	c.2349delG	Frameshift	Novel	Negative	Negative
P0480	Male	52	Asian	PDAC	*MSH2*	p.Lys423Glu	chr2:47657071	c.1267A>G	Missense	VUS	Cholangiocarcinoma	Negative
P0481	Male	49	Asian	PDAC	*PMS2*	p.His435Tyr	chr7:6027093	c.1303C>T	Missense	VUS	Negative	Negative
P0486	Male	61	Asian	PDAC	*RAD51B*	p.Tyr140Cys	chr14:68331823	c.419A>G	Missense	Novel	Negative	Negative
P0488	Female	74	Asian	PDAC	*CEL*	p.Pro323Leu	chr9:135944122	c.968C>T	Missense	Novel	Negative	Negative
P0493	Male	68	Asian	PDAC	*ATM*	p.Ser2941Ala	chr11:108225572	c.8821T>G	Missense	VUS	Negative	Brother with pancreatic cancer
P0494	Male	70	Asian	PDAC	*PNLIPRP2*	p.Thr448Asn	chr10:118401790	c.1345C>A	Missense	Novel	Negative	Negative
P0501	Male	78	Asian	PDAC	*BRCA1*	p.Thr1196Ile	chr17:41243961	c.3587C>T	Missense	VUS	Negative	Negative
P0502	Female	75	Asian	PDAC	*CELA3B*	p.Ser16Ter	chr1:22304865	c.47C>G	Nonsense	Novel	Negative	Negative
P0502	Female	75	Asian	PDAC	*TNS3*	p.Val218Phe	chr7:47451396	c.652G>T	Missense	Novel	Negative	Negative
P0512	Male	75	Asian	PDAC	*ATM*	p.Val2367Ile	chr11:108199757	c.7099G>A	Missense	VUS	Negative	Negative
P0513	Male	67	Asian	PDAC	*FANCC*	p.Leu486Pro	chr9:97869424	c.1457T>C	Missense	Novel	Negative	Negative
P0516	Female	62	Asian	PDAC	*RECQL4*	p.Tyr519fs	chr8:145740384	c.1555_1556insC	Frameshift	Novel	Negative	Negative
P0518	Male	47	Asian	PDAC	*PMS2*	p.Val200Ile	chr7:6038846	c.598G>A	Missense	VUS	Negative	Negative
P0520	Male	44	Asian	PDAC	*BRCA2*	p.Arg2991Cys	chr13:32953904	c.8971C>T	Missense	VUS	Negative	Negative
P0525	Male	62	Asian	PDAC	*BRCA2*	p.Arg2973Gly	chr13:32953616	c.8917C>G	Missense	VUS	Negative	Negative
P0526	Male	54	Asian	PDAC	*MSH2*	p.Phe366Leu	chr2:47656900	c.1096T>C	Missense	Novel	Negative	Negative
P0532	Female	77	Asian	PDAC	*CELA3A*	p.Asp184His	chr1:22333916	c.550G>C	Missense	Novel	Negative	Negative
P0541	Male	72	Asian	PDAC	*ATM*	p.Arg2461His	chr11:108201015	c.7382G>A	Missense	VUS	Negative	Negative
P0541	Male	72	Asian	PDAC	*BRIP1*	p.Asn643Ile	chr17:59857629	c.1928A>T	Missense	Novel	Negative	Negative
P0542	Female	61	Asian	PDAC	*MSH2*	p.Phe131Val	chr2:47637257	c.391T>G	Missense	VUS	Negative	Negative
P0551	Male	59	Asian	PDAC	*MSH6*	p.Asn1020Asp	chr2:48028180	c.3058A>G	Missense	VUS	Negative	Negative
P0553	Male	62	Asian	PDAC	*STK11*	p.Thr189Ile	chr19:1220473	c.566C>T	Missense	VUS	Negative	Negative
P0553	Male	62	Asian	PDAC	*FANCL*	p.Leu91fs	chr2:58453863	c.272delT	Frameshift	Novel	Negative	Negative
P0554	Male	59	Asian	PDAC	*MLH1*	p.Asp450Tyr	chr3:37067437	c.1348G>T	Missense	VUS	Negative	Negative
P0558	Female	44	Asian	PDAC	*FANCA*	p.Pro799Ser	chr16:89836354	c.2395C>T	Missense	VUS	Negative	Negative
P0558	Female	44	Asian	PDAC	*TNS3*	p.Gly4Val	chr7:47479224	c.11G>T	Missense	Novel	Negative	Negative
P0562	Male	66	Asian	PDAC	*ATM*	p.Ala59Val	chr11:108098606	c.176C>T	Missense	VUS	Negative	Negative

© 2022 Yin L et al. *JAMA Network Open*.

P0572	Male	75	Asian	PDAC	*ATM*	p.Ala1954Gly	chr11:108180985	c.5861C>G	Missense	VUS	Negative	Negative
P0579	Female	65	Asian	PDAC	*BRCA1*	p.Met18Val	chr17:41276062	c.52A>G	Missense	VUS	Negative	Negative
P0589	Male	63	Asian	PDAC	*ATM*	p.Leu3048Val	chr11:108236206	c.9142C>G	Missense	VUS	Negative	Negative
P0589	Male	63	Asian	PDAC	*FANCG*	p.Val244Met	chr9:35077015	c.730G>A	Missense	VUS	Negative	Negative
P0591	Male	71	Asian	PDAC	*ERCC4*	p.Arg158fs	chr16:14020500	c.471_472insT	Frameshift	Novel	Negative	Negative
P0596	Male	70	Asian	PDAC	*CEL*	p.Gly137Val	chr9:135940487	c.410G>T	Missense	Novel	Negative	Negative
P0619	Male	72	Asian	Ampullary cancer	*MSH6*	p.Gly1069Arg	chr2:48030591	c.3205G>C	Missense	VUS	Negative	Negative
P0620	Male	58	Asian	PDAC	*PNLIPRP2*	p.Arg90Cys	chr10:118385520	c.270C>T	Missense	Novel	Negative	Negative
P0621	Male	65	Asian	PDAC	*ATM*	p.Ile879Val	chr11:108138066	c.2635A>G	Missense	VUS	Negative	Negative
P0626	Female	54	Asian	Cholangiocarcinoma	*ATM*	p.Phe802Leu	chr11:108129740	c.2404T>C	Missense	Novel	Negative	Negative
P0627	Female	51	Asian	PDAC	*BRCA2*	p.Lys2496Asn	chr13:32930617	c.7488G>C	Missense	VUS	Negative	Negative
P0645	Male	42	Asian	PDAC	*CFTR*	p.Arg1358Lys	chr7:117304851	c.4073G>A	Missense	Novel	Negative	Negative
P0653	Female	72	Asian	PDAC	*FANCA*	p.Arg600His	chr16:89845236	c.1799G>A	Missense	Novel	Lung cancer	Negative
P0667	Female	61	Asian	PDAC	*BRIP1*	p.Ser409Gly	chr17:59876576	c.1225A>G	Missense	Novel	Negative	Negative
P0678	Male	65	Asian	PDAC	*PALB2*	p.Asp446Gly	chr16:23646530	c.1337A>G	Missense	VUS	Negative	Negative
P0682	Male	64	Asian	PDAC	*ATM*	p.Ala1079Ser	chr11:108143530	c.3235G>T	Missense	Novel	Negative	Negative
P0684	Male	61	Asian	PDAC	*ARID1A*	p.Arg1658Trp	chr1:27101690	c.4972C>T	Missense	Novel	Negative	Negative
P0687	Male	70	Asian	PDAC	*CDH1*	p.Ala289Thr	chr16:68845619	c.865G>A	Missense	VUS	Negative	Negative
P0692	Male	66	Asian	PDAC	*CTRB1*	p.Gly18Arg	chr16:75252957	c.52G>C	Missense	Novel	Negative	Negative
P0695	Male	74	Asian	PDAC	*CDH1*	p.Pro88Arg	chr16:68835672	c.263C>G	Missense	VUS	Gastric cancer	Negative
P0701	Female	59	Asian	PDAC	*BUB3*	p.Asp96fs	chr10:124917265	c.287delA	Frameshift	Novel	Negative	Negative
P0702	Male	69	Asian	PDAC	*ARID1A*	p.Ala183Thr	chr1:27023441	c.547G>A	Missense	Novel	Negative	Negative
P0706	Female	63	Asian	PDAC	*CTRC*	p.His110Tyr	chr1:15769040	c.328C>T	Missense	Novel	Negative	Negative
P0708	Female	63	Asian	PDAC	*CPA1*	p.Phe20Leu	chr7:130020421	c.60T>G	Missense	Novel	Negative	Negative
P0721	Female	65	Asian	PDAC	*PNLIPRP2*	p.Asp124Asn	chr10:118386414	c.372G>A	Missense	Novel	Negative	Negative
P0724	Male	53	Asian	PDAC	*XRCC3*	p.Ser110Leu	chr14:104173417	c.329C>T	Missense	Novel	Negative	Negative
P0733	Male	66	Asian	PDAC	*CFTR*	p.Glu621Ter	chr7:117232080	c.1860_1861insT	Nonsense	Novel	Negative	Negative
P0735	Male	64	Asian	PDAC	*RAD51C*	p.Arg12Gly	chr17:56770038	c.34C>G	Missense	Novel	Negative	Negative
P0737	Female	62	Asian	PDAC	*CTRC*	p.Trp191Arg	chr1:15771178	c.571T>C	Missense	Novel	Negative	Negative
P0739	Female	65	Asian	PDAC	*PNLIPRP2*	p.Thr176Lys	chr10:118387335	c.529C>A	Missense	Novel	Negative	Negative

© 2022 Yin L et al. *JAMA Network Open*.

P0739	Female	65	Asian	PDAC	*ATM*	p.Asp1540Tyr	chr11:108164046	c.4618G>T	Missense	VUS	Negative	Negative
P0741	Male	70	Asian	PDAC	*BRCA2*	p.Asn1718Lys	chr13:32913646	c.5154T>G	Missense	Novel	Negative	Negative
P0742	Female	72	Asian	PDAC	*RNF43*	p.Glu284fs	chr17:56437610	c.851_852insT	Frameshift	Novel	Breast cancer	Negative
P0747	Female	76	Asian	PDAC	*CEL*	p.Pro323Leu	chr9:135944122	c.968C>T	Missense	Novel	Negative	Negative
P0749	Male	60	Asian	PDAC	*CPB1*	p.Trp128Cys	chr3:148558672	c.384G>C	Missense	Novel	Negative	Negative
P0751	Male	68	Asian	PDAC	*CFTR*	p.Val1272Gly	chr7:117282589	c.3815T>G	Missense	Novel	Negative	Negative
P0754	Male	56	Asian	PDAC	*PALB2*	p.His394Leu	chr16:23646686	c.1181A>T	Missense	Novel	Negative	Negative
P0764	Female	67	Asian	PDAC	*NOC2L*	p.Leu76Val	chr1:892607	c.226C>G	Missense	Novel	Negative	Negative
P0768	Male	62	Asian	PDAC	*PMS2*	p.Gln244Arg	chr7:6037029	c.731A>G	Missense	Novel	Negative	Negative
P0778	Female	61	Asian	PDAC	*FANCA*	p.Pro799Ser	chr16:89836354	c.2395C>T	Missense	VUS	Negative	Negative
P0778	Female	61	Asian	PDAC	*PMS2*	p.Asp526Glu	chr7:6026818	c.1578C>G	Missense	VUS	Negative	Negative
P0784	Male	40	Asian	PDAC	*CPB1*	p.Glu192Asp	chr3:148559711	c.576G>C	Missense	Novel	Negative	Negative
P0788	Male	66	Asian	PDAC	*BRCA2*	p.Leu1240fs	chr13:32912206	c.3717_3718insA	Frameshift	Novel	Negative	Negative
P0789	Female	61	Asian	PDAC	*MLH1*	p.Asn248Ser	chr3:37055988	c.743A>G	Missense	VUS	Negative	Negative
P0808	Male	53	Asian	Chronic pancreatitis	*CPA1*	p.Phe52Leu	chr7:130021477	c.154T>C	Missense	Novel	Negative	Negative
P0818	Female	36	Asian	Chronic pancreatitis	*ATM*	p.Asn1431Ser	chr11:108160384	c.4292A>G	Missense	VUS	Negative	Negative
P0820	Male	54	Asian	Chronic pancreatitis	*BRCA2*	p.Ile1664Phe	chr13:32913482	c.4990A>T	Missense	Novel	Negative	Negative
P0820	Male	54	Asian	Chronic pancreatitis	*NOC2L*	p.Gln599Ter	chr1:881790	c.1795C>T	Nonsense	Novel	Negative	Negative
P0830	Male	46	Asian	Acute pancreatitis	*ARID1A*	p.Ala1522Thr	chr1:27101282	c.4564G>A	Missense	Novel	Negative	Negative
P0830	Male	46	Asian	Acute pancreatitis	*BRCA2*	p.Asp1864Asn	chr13:32914082	c.5590G>A	Missense	VUS	Negative	Negative
P0830	Male	46	Asian	Acute pancreatitis	*CFTR*	p.Gly1237Asp	chr7:117267817	c.3710G>A	Missense	VUS	Negative	Negative
P0832	Male	79	Asian	Acute pancreatitis	*ATM*	p.Pro37His	chr11:108098540	c.110C>A	Missense	Novel	Negative	Negative
P0832	Male	79	Asian	Acute pancreatitis	*PALB2*	p.Ile966Val	chr16:23634390	c.2896A>G	Missense	VUS	Negative	Negative
P0835	Male	42	Asian	Acute pancreatitis	*BRIP1*	p.Leu340Phe	chr17:59878736	c.1018C>T	Missense	VUS	Negative	Negative
P0838	Male	38	Asian	Acute pancreatitis	*CELA2A*	p.Gly242Ala	chr1:15793966	c.725G>C	Missense	Novel	Negative	Negative
P0839	Female	60	Asian	Acute pancreatitis	*IDO2*	p.Thr149Met	chr8:39840262	c.446C>T	Missense	Novel	Negative	Negative
P0843	Female	63	Asian	Acute pancreatitis	*NOC2L*	p.His111Arg	chr1:892501	c.332A>G	Missense	Novel	Negative	Negative

© 2022 Yin L et al. *JAMA Network Open*.

P0845	Male	81	Asian	Acute pancreatitis	*RECQL4*	p.Pro597Ser	chr8:145739662	c.1789C>T	Missense	VUS	Negative	Negative
P0850	Male	55	Asian	Acute pancreatitis	*BRCA2*	p.Ile729Thr	chr13:32910678	c.2186T>C	Missense	VUS	Negative	Negative
P0850	Male	55	Asian	Acute pancreatitis	*BAP1*	p.Arg518Trp	chr3:52437609	c.1552C>T	Missense	VUS	Negative	Negative
P0862	Female	55	Asian	Acute pancreatitis	*CFTR*	p.Ala1009Thr	chr7:117250609	c.3025G>A	Missense	VUS	Negative	Negative
P0865	Male	83	Asian	Acute pancreatitis	*ERBB2*	p.Gly292Ser	chr17:37866707	c.874G>A	Missense	Novel	Negative	Negative
P0866	Female	42	Asian	Acute pancreatitis	*TNS3*	p.Pro289Ser	chr7:47440044	c.865C>T	Missense	Novel	Negative	Negative
P0868	Female	68	Asian	Acute pancreatitis	*ATM*	p.Gly1788Ser	chr11:108173622	c.5362G>A	Missense	VUS	Negative	Negative
P0881	Male	24	Asian	Acute pancreatitis	*GZMK*	p.Gly215Glu	chr5:54329603	c.644G>A	Missense	Novel	Negative	Negative
P0893	Male	73	Asian	PDAC	*RAD51C*	p.Arg370Ter	chr17:56811560	c.1108C>T	Nonsense	VUS	Negative	Negative
P0907	Male	70	Asian	PDAC	*TP53*	p.Pro92Thr	chr17:7579413	c.274C>A	Missense	Novel	Negative	Negative
P0913	Female	25	Asian	SPT	*BAP1*	p.Thr467Pro	chr3:52437762	c.1399A>C	Missense	VUS	Negative	Negative
P0918	Female	50	Asian	SPT	*CFTR*	p.Gly1349Ser	chr7:117304823	c.4045G>A	Missense	VUS	Negative	Negative
P0918	Female	50	Asian	SPT	*MSH6*	p.Ala494Pro	chr2:48026602	c.1480G>C	Missense	Novel	Negative	Negative
P0919	Male	74	Asian	IPMN	*BRIP1*	p.Leu1105Phe	chr17:59761092	c.3315G>T	Missense	Novel	Negative	Negative
P0919	Male	74	Asian	IPMN	*BUB1B*	p.Ser529fs	chr15:40494622	c.1585_1586insT	Frameshift	Novel	Negative	Negative
P0920	Female	18	Asian	MCN	*BRCA2*	p.Ile2628Met	chr13:32936738	c.7884A>G	Missense	VUS	Negative	Negative
P0921	Female	26	Asian	SPT	*BRCA1*	p.Pro115Leu	chr17:41256236	c.344C>T	Missense	VUS	Negative	Negative
P0928	Male	39	Asian	PNET	*NOC2L*	p.Thr616fs	chr1:881625	c.1844_1845insT	Frameshift	Novel	Negative	Negative
P0932	Female	54	Asian	PDAC	*ARID1A*	p.Gln1327fs	chr1:27100181	c.3977_3978insCGCA	Frameshift	Novel	Negative	Negative
P0932	Female	54	Asian	PDAC	*CFTR*	p.Phe508fs	chr7:117199645	c.1520_1521insA	Frameshift	Novel	Negative	Negative
P0935	Female	67	Asian	PDAC	*BRIP1*	p.Met1Val	chr17:59938900	c.1A>G	Missense	VUS	Negative	Negative
P0938	Male	41	Asian	Acute pancreatitis	*ERBB2*	p.Tyr554Asn	chr17:37872781	c.1660T>A	Missense	Novel	Negative	Negative
P0939	Female	33	Asian	MCN	*PMS2*	p.Val200Ile	chr7:6038846	c.598G>A	Missense	VUS	Negative	Negative
P0941	Male	59	Asian	IPMN	*PNLIPRP2*	p.Lys117fs	chr10:118386389	c.350_351delGA	Frameshift	Novel	Negative	Negative
P0949	Male	54	Asian	PNET	*BRCA1*	p.His1455Leu	chr17:41231410	c.4364A>T	Missense	Novel	Negative	Negative
P0951	Female	29	Asian	SPT	*CPB1*	p.Pro313Arg	chr3:148563370	c.938C>G	Missense	Novel	Negative	Negative
P0953	Female	58	Asian	PNET	*ARID1A*	p.Arg1566fs	chr1:27101415	c.4697_4699delGGC	Frameshift	Novel	Negative	Negative

© 2022 Yin L et al. *JAMA Network Open*.

P0955	Female	74	Asian	MCN	*ATM*	p.Asn2875Asp	chr11:108218044	c.8623A>G	Missense	Novel	Negative	Negative
P0958	Female	74	Asian	PDAC	*ATM*	p.Lys50Gln	chr11:108098578	c.148A>C	Missense	VUS	Negative	Negative
P0968	Female	48	Asian	PNET	*BRCA2*	p.Glu3377Asp	chr13:32972781	c.10131A>C	Missense	VUS	Negative	Negative
P0971	Male	29	Asian	SPT	*FANCC*	p.Gln283Pro	chr9:97888859	c.848A>C	Missense	Novel	Negative	Negative
P0975	Female	63	Asian	PNET	*FANCA*	p.Ser119Cys	chr16:89877407	c.356C>G	Missense	VUS	Negative	Negative
P0977	Female	46	Asian	PNET	*BAP1*	p.Arg389His	chr3:52438553	c.1166G>A	Missense	Novel	Negative	Negative
P0994	Female	30	Asian	PNET	*MSH6*	p.Pro1073Arg	chr2:48030604	c.3218C>G	Missense	VUS	Negative	Negative
P1001	Female	56	Asian	SPT	*BRCA2*	p.Asn2208Ile	chr13:32915115	c.6623A>T	Missense	Novel	Breast cancer	Negative
P1020	Male	26	Asian	Acute pancreatitis	*PALB2*	p.Met416Thr	chr16:23646620	c.1247T>C	Missense	Novel	Negative	Negative
P1022	Female	63	Asian	Chronic pancreatitis	*ARID1A*	p.His205Arg	chr1:27023508	c.614A>G	Missense	Novel	Negative	Negative
P1024	Male	42	Asian	SCN	*FANCA*	p.Leu518Pro	chr16:89849428	c.1553T>C	Missense	Novel	Negative	Negative
P1027	Male	50	Asian	Chronic pancreatitis	*MLH1*	p.Lys254Arg	chr3:37056006	c.761A>G	Missense	VUS	Colon polyp	Negative
P1028	Male	59	Asian	IPMN	*ARID1A*	p.Arg2153Cys	chr1:27106846	c.6457C>T	Missense	Novel	Negative	Negative
P1034	Male	37	Asian	Chronic pancreatitis	*ATM*	p.Leu1111Phe	chr11:108150264	c.3331C>T	Missense	Novel	Negative	Negative
P1038	Male	52	Asian	Chronic pancreatitis	*RECQL4*	p.His745Tyr	chr8:145738832	c.2233C>T	Missense	VUS	Negative	Negative
P1040	Male	50	Asian	Chronic pancreatitis	*SPINK1*	p.Lys31fs	chr5:147207677	c.93_101delATG TTACAA	Frameshift	Novel	Negative	Negative
P1041	Male	48	Asian	Chronic pancreatitis	*PNLIPRP 2*	p.Gly235Arg	chr10:118390754	c.705G>A	Missense	Novel	Negative	Negative
P1044	Male	55	Asian	Chronic pancreatitis	*RECQL4*	p.Ala919Thr	chr8:145738230	c.2755G>A	Missense	VUS	Negative	Negative
P1046	Female	15	Asian	Chronic pancreatitis	*ERCC4*	p.Gln5Ter	chr16:14014035	c.13C>T	Nonsense	Novel	Negative	Negative
P1046	Female	15	Asian	Chronic pancreatitis	*MLH1*	p.Asp235Val	chr3:37055949	c.704A>T	Missense	VUS	Negative	Negative
P1050	Male	59	Asian	IPMN	*BRCA2*	p.Ile2693Lys	chr13:32937417	c.8078T>A	Missense	VUS	Negative	Negative
P1060	Female	60	Asian	IPMN	*NOC2L*	p.Arg417Gln	chr1:887461	c.1250G>A	Missense	Novel	Negative	Mother with pancreatic tumor
P1067	Male	48	Asian	IPMN	*CELA3A*	p.Gln176Pro	chr1:22333893	c.527A>C	Missense	Novel	Negative	Negative
P1068	Male	62	Asian	PNET	*FANCL*	p.Asp213Asn	chr2:58392928	c.637G>A	Missense	VUS	Negative	Negative
P1069	Male	71	Asian	PNET	*CELA2A*	p.Gly218Arg	chr1:15793893	c.652G>A	Missense	Novel	Negative	Negative
P1077	Male	45	Asian	IPMN	*CDH1*	p.Thr623Ala	chr16:68856059	c.1867A>G	Missense	VUS	Negative	Negative
P1081	Male	64	Asian	IPMN	*RECQL4*	p.Leu640Val	chr8:145739452	c.1918C>G	Missense	Novel	Renal cacner	Negative

© 2022 Yin L et al. *JAMA Network Open*.

P1089	Male	67	Asian	IPMN	*CEL*	p.Val123Ile	chr9:135940444	c.367G>A	Missense	Novel	Negative	Negative
P1091	Male	36	Asian	SPT	*TNS3*	p.Thr1214Met	chr7:47336715	c.3641C>T	Missense	Novel	Negative	Negative
P1094	Female	35	Asian	MCN	*ARID1A*	p.Arg1658Trp	chr1:27101690	c.4972C>T	Missense	Novel	Negative	Negative
P1096	Female	55	Asian	PNET	*TP53*	p.Ser371Cys	chr17:7572997	c.1112C>G	Missense	Novel	Negative	Negative
P1101	Female	26	Asian	SPT	*PNLIPRP2*	p.Ala145Val	chr10:118386478	c.436C>T	Missense	Novel	Negative	Negative
P1102	Female	64	Asian	IPMN	*TNS3*	p.Gly187Asp	chr7:47454718	c.560G>A	Missense	Novel	Negative	Negative
P1109	Female	77	Asian	IPMN	*ERBB2*	p.Ile148fs	chr17:37865573	c.442_443insC	Frameshift	Novel	Negative	Negative
P1113	Male	53	Asian	PDAC	*RECQL4*	p.Arg715Gln	chr8:145739011	c.2144G>A	Missense	VUS	Negative	Negative
P1115	Male	63	Asian	PNET	*RNF43*	p.Arg668Trp	chr17:56435135	c.2002C>T	Missense	Novel	Negative	Negative
P1117	Female	47	Asian	SPT	*PALB2*	p.His553Gln	chr16:23646208	c.1659C>A	Missense	VUS	Negative	Negative
P1120	Male	44	Asian	PNET	*PALB2*	p.Thr51Ile	chr16:23649230	c.152C>T	Missense	VUS	Negative	Negative
P1125	Female	13	Asian	SPT	*CPB1*	p.Pro313Arg	chr3:148563370	c.938C>G	Missense	Novel	Negative	Negative
P1126	Female	25	Asian	SPT	*BRCA2*	p.Lys2496Asn	chr13:32930617	c.7488G>C	Missense	VUS	Negative	Negative
P1146	Female	54	Asian	IPMN	*TNS3*	p.Ser1122Cys	chr7:47342640	c.3365C>G	Missense	Novel	Negative	Negative
P1159	Female	68	Asian	PNET	*ERCC4*	p.Leu27Phe	chr16:14014101	c.79C>T	Missense	VUS	Negative	Negative
P1168	Male	11	Asian	SPT	*CELA3B*	p.Val198Met	chr1:22310774	c.592G>A	Missense	Novel	Negative	Negative
P1173	Male	81	Asian	Chronic pancreatitis	*BRCA2*	p.Gln2345Glu	chr13:32929023	c.7033C>G	Missense	VUS	Negative	Negative
P1173	Male	81	Asian	Chronic pancreatitis	*FANCC*	p.Arg433fs	chr9:97873775	c.1297delC	Frameshift	Novel	Negative	Negative
P1180	Male	72	Asian	IPMN	*PALB2*	p.Pro117Leu	chr16:23647517	c.350C>T	Missense	VUS	Negative	Negative
P1183	Male	41	Asian	PNET	*BRCA2*	p.Val2010Gly	chr13:32914521	c.6029T>G	Missense	VUS	Negative	Negative
P1186	Male	72	Asian	Acute pancreatitis	*FANCA*	p.Thr1161Lys	chr16:89813023	c.3482C>A	Missense	Novel	Negative	Negative
P1187	Female	56	Asian	PNET	*BRCA2*	p.Ser1074Cys	chr13:32911712	c.3220A>T	Missense	VUS	Negative	Negative
P1193	Female	63	Asian	PNET	*ATM*	p.His1895Arg	chr11:108178633	c.5684A>G	Missense	Novel	Negative	Negative
P1201	Male	68	Asian	IPMN	*BRCA2*	p.Asp651Gly	chr13:32910444	c.1952A>G	Missense	VUS	Negative	Negative
P1202	Female	73	Asian	IPMN	*FANCA*	p.Pro759Leu	chr16:89836614	c.2276C>T	Missense	Novel	Negative	Negative
P1203	Male	52	Asian	Acute pancreatitis	*RNF43*	p.Trp416Ter	chr17:56435889	c.1248G>A	Nonsense	Novel	Negative	Negative
P1208	Female	48	Asian	Acute pancreatitis	*ATM*	p.Val1085Ile	chr11:108143548	c.3253G>A	Missense	Novel	Negative	Negative
P1214	Female	59	Asian	Acute pancreatitis	*ARID1A*	p.Pro680Ser	chr1:27087464	c.2038C>T	Missense	Novel	Negative	Negative
P1216	Male	58	Asian	Acute pancreatitis	*BAP1*	p.Leu294fs	chr3:52439832	c.879_880insGC	Frameshift	Novel	Negative	Negative

© 2022 Yin L et al. *JAMA Network Open*.

P1216	Male	58	Asian	Acute pancreatitis	*ERCC4*	p.Val133Leu	chr16:14020426	c.397G>C	Missense	Novel	Negative	Negative
P1226	Male	52	Asian	Acute pancreatitis	*CPB1*	p.Leu93Gln	chr3:148558478	c.278T>A	Missense	Novel	Negative	Negative
P1232	Male	37	Asian	Acute pancreatitis	*MSH6*	p.Pro1087His	chr2:48030646	c.3260C>A	Missense	VUS	Negative	Negative
P1256	Male	14	Asian	Acute pancreatitis	*CTRC*	p.His110Tyr	chr1:15769040	c.328C>T	Missense	Novel	Negative	Negative
P1258	Female	82	Asian	Acute pancreatitis	*FANCG*	p.Arg563Gln	chr9:35074440	c.1688G>A	Missense	VUS	Negative	Negative
P1262	Female	64	Asian	Acute pancreatitis	*CPA1*	p.Arg27Gln	chr7:130020953	c.80G>A	Missense	Novel	Negative	Negative
P1262	Female	64	Asian	Acute pancreatitis	*TNS3*	p.Gly187Asp	chr7:47454718	c.560G>A	Missense	Novel	Negative	Negative
P1265	Male	20	Asian	Acute pancreatitis	*MSH2*	p.Asp180Val	chr2:47637405	c.539A>T	Missense	Novel	Negative	Negative
P1273	Male	33	Asian	Acute pancreatitis	*BRCA1*	p.Val1675Ala	chr17:41222970	c.5024T>C	Missense	Novel	Negative	Negative
P1279	Male	44	Asian	Acute pancreatitis	*XRCC3*	p.Arg174Ter	chr14:104169551	c.520C>T	Nonsense	Novel	Negative	Negative
P1280	Male	73	Asian	Acute pancreatitis	*TNS3*	p.Pro177His	chr7:47454748	c.530C>A	Missense	Novel	Negative	Negative
P1280	Male	73	Asian	Acute pancreatitis	*XRCC3*	p.Lys72fs	chr14:104173529	c.214_216delAAG	Frameshift	Novel	Negative	Negative
P1288	Female	72	Asian	Acute pancreatitis	*BUB1B*	p.Arg381fs	chr15:40488828	c.1141_1142insAG	Frameshift	Novel	Negative	Negative
P1291	Female	40	Asian	Acute pancreatitis	*BAP1*	p.Ser489Arg	chr3:52437696	c.1465A>C	Missense	Novel	Negative	Negative
P1291	Female	40	Asian	Acute pancreatitis	*TERT*	p.Arg1097fs	chr5:1254489	c.3288_3289insG	Frameshift	Novel	Negative	Negative
P1293	Female	61	Asian	Acute pancreatitis	*ATM*	p.Lys2749Ile	chr11:108206666	c.8246A>T	Missense	VUS	Negative	Negative
P1293	Female	61	Asian	Acute pancreatitis	*FANCA*	p.Cys1159Ser	chr16:89813029	c.3476G>C	Missense	VUS	Negative	Negative
P1295	Female	75	Asian	Acute pancreatitis	*BRCA2*	p.Ser353Leu	chr13:32906673	c.1058C>T	Missense	Novel	Negative	Negative
P1295	Female	75	Asian	Acute pancreatitis	*CELA2A*	p.Gln174Ter	chr1:15792520	c.520C>T	Nonsense	Novel	Negative	Negative
P1306	Male	39	Asian	Acute pancreatitis	*TERT*	p.Thr564Ser	chr5:1282623	c.1690A>T	Missense	Novel	Negative	Negative
P1312	Female	52	Asian	Acute pancreatitis	*TP53*	p.Pro72Ala	chr17:7579473	c.214C>G	Missense	VUS	Negative	Negative
P1313	Male	27	Asian	Acute pancreatitis	*TERT*	p.Thr1098fs	chr5:1254485	c.3292_3293insG	Frameshift	Novel	Negative	Negative
P1316	Male	31	Asian	Acute pancreatitis	*ARID1A*	p.Gln761fs	chr1:27088668	c.2281delC	Frameshift	Novel	Negative	Negative

© 2022 Yin L et al. *JAMA Network Open*.

P1316	Male	31	Asian	Acute pancreatitis	*NOC2L*	p.Arg628Gly	chr1:881588	c.1882C>G	Missense	Novel	Negative	Negative
P1319	Female	58	Asian	Acute pancreatitis	*HNF1B*	p.His515Tyr	chr17:36059192	c.1543C>T	Missense	Novel	Negative	Negative
P1323	Male	45	Asian	Acute pancreatitis	*ARID1A*	p.Gly882fs	chr1:27089687	c.2643_2644insC	Frameshift	Novel	Negative	Negative
P1323	Male	45	Asian	Acute pancreatitis	*BAP1*	p.Tyr241His	chr3:52440331	c.721T>C	Missense	Novel	Negative	Negative
P1325	Female	52	Asian	Acute pancreatitis	*PALB2*	p.Thr710Met	chr16:23641346	c.2129C>T	Missense	VUS	Negative	Negative
P1327	Male	32	Asian	Acute pancreatitis	*MSH2*	p.Thr552Ser	chr2:47693941	c.1655C>G	Missense	Novel	Negative	Negative
P1329	Male	76	Asian	Acute pancreatitis	*RECQL4*	p.Arg375His	chr8:145741379	c.1124G>A	Missense	VUS	Negative	Negative
P1331	Male	34	Asian	Acute pancreatitis	*GRP*	p.Met50fs	chr18:56892726	c.143_144insT	Frameshift	Novel	Negative	Negative
P1332	Female	37	Asian	Acute pancreatitis	*BUB1*	p.Gln144fs	chr2:111428138	c.429_430insT	Frameshift	Novel	Negative	Negative
P1333	Male	38	Asian	Acute pancreatitis	*BRCA2*	p.Tyr42Cys	chr13:32893271	c.125A>G	Missense	VUS	Negative	Negative
P1336	Male	19	Asian	Acute pancreatitis	*BRCA2*	p.Glu2082Lys	chr13:32914736	c.6244G>A	Missense	Novel	Negative	Negative
P1338	Male	45	Asian	Acute pancreatitis	*CFTR*	p.Gly1069Arg	chr7:117251700	c.3205G>A	Missense	VUS	Negative	Negative
P1344	Female	38	Asian	Acute pancreatitis	*BRCA1*	p.Thr796Ile	chr17:41245161	c.2387C>T	Missense	VUS	Negative	Negative
P1348	Male	70	Asian	Acute pancreatitis	*CFTR*	p.Lys978fs	chr7:117246750	c.2933_2934delAA	Frameshift	Novel	Negative	Negative
P1352	Male	45	Asian	Acute pancreatitis	*MSH2*	p.Ser142Leu	chr2:47637291	c.425C>T	Missense	Novel	Negative	Negative
P1355	Female	53	Asian	Acute pancreatitis	*PMS2*	p.His435Tyr	chr7:6027093	c.1303C>T	Missense	VUS	Negative	Negative
P1365	Male	52	Asian	PDAC	*MLH1*	p.Met342Val	chr3:37061940	c.1024A>G	Missense	VUS	Negative	Negative
P1369	Male	41	Asian	Acute pancreatitis	*ERBB2*	p.Thr323fs	chr17:37868244	c.965_966insC	Frameshift	Novel	Negative	Negative
P1369	Male	41	Asian	Acute pancreatitis	*PNLIPRP2*	p.Ile78Thr	chr10:118385485	c.235T>C	Missense	Novel	Negative	Negative
P1376	Female	50	Asian	Acute pancreatitis	*TNS3*	p.Pro375Leu	chr7:47409119	c.1124C>T	Missense	Novel	Negative	Negative
P1388	Female	68	Asian	Acute pancreatitis	*ATM*	p.Arg785Cys	chr11:108128310	c.2353C>T	Missense	VUS	Negative	Negative
P1394	Male	64	Asian	Acute pancreatitis	*BRIP1*	p.Gln102Arg	chr17:59934493	c.305A>G	Missense	VUS	Negative	Negative
P1396	Female	63	Asian	Acute pancreatitis	*CELA3A*	p.His187fs	chr1:22333926	c.560_561insG	Frameshift	Novel	Negative	Negative

© 2022 Yin L et al. *JAMA Network Open*.

P1396	Female	63	Asian	Acute pancreatitis	*CPB1*	p.Ala263fs	chr3:148563217	c.785_786insT	Frameshift	Novel	Negative	Negative
P1396	Female	63	Asian	Acute pancreatitis	*CTRC*	p.Val180Met	chr1:15771145	c.538G>A	Missense	Novel	Negative	Negative
P1403	Female	62	Asian	PDAC	*CPA1*	p.Gly277Ser	chr7:130025028	c.829G>A	Missense	Novel	Negative	Negative
P1411	Male	59	Asian	PDAC	*CELA2A*	p.Asn213Lys	chr1:15792639	c.639C>G	Missense	Novel	Negative	Negative
P1413	Female	47	Asian	PDAC	*CPB1*	p.Trp128Cys	chr3:148558672	c.384G>C	Missense	Novel	Negative	Negative
P1415	Male	78	Asian	PDAC	*RAD51C*	p.Tyr216His	chr17:56780631	c.646T>C	Missense	Novel	Negative	Negative
P1416	Male	61	Asian	PDAC	*PMS2*	p.Ile144Asn	chr7:6042190	c.431T>A	Missense	Novel	Negative	Negative
P1418	Male	61	Asian	PDAC	*FANCA*	p.Pro799Ser	chr16:89836354	c.2395C>T	Missense	VUS	Negative	Negative
P1420	Female	67	Asian	PDAC	*FANCA*	p.Pro799Ser	chr16:89836354	c.2395C>T	Missense	VUS	Negative	Negative
P1426	Female	63	Asian	PDAC	*FANCA*	p.Pro799Ser	chr16:89836354	c.2395C>T	Missense	VUS	Negative	Negative
P1428	Female	60	Asian	PDAC	*FANCA*	p.Pro799Ser	chr16:89836354	c.2395C>T	Missense	VUS	Negative	Negative
P1435	Female	41	Asian	PDAC	*BRIP1*	p.Asn643Ile	chr17:59857629	c.1928A>T	Missense	Novel	Negative	Negative
P1439	Male	54	Asian	PDAC	*CDH1*	p.Val391Ile	chr16:68847249	c.1171G>A	Missense	VUS	Negative	Negative
P1440	Male	68	Asian	PDAC	*MSH6*	p.Ala1127Thr	chr2:48030765	c.3379G>A	Missense	VUS	Negative	Negative
P1442	Female	62	Asian	PDAC	*BRCA1*	p.Glu1815Lys	chr17:41201164	c.5443G>A	Missense	Novel	Negative	Negative
P1443	Female	61	Asian	PDAC	*CTRC*	p.Arg119His	chr1:15769068	c.356G>A	Missense	VUS	Negative	Negative
P1450	Male	67	Asian	PDAC	*ATM*	p.Val2166Ala	chr11:108192072	c.6497T>C	Missense	VUS	Negative	Negative
P1456	Male	63	Asian	PDAC	*ARID1A*	p.Val973Ile	chr1:27092986	c.2917G>A	Missense	Novel	Negative	Negative
P1461	Female	68	Asian	PDAC	*MLH1*	p.Asp428Gly	chr3:37067372	c.1283A>G	Missense	Novel	Negative	Negative
P1463	Male	71	Asian	PDAC	*MSH2*	p.Thr8Ser	chr2:47630352	c.22A>T	Missense	VUS	Negative	Negative
P1464	Male	74	Asian	PDAC	*BRCA2*	p.Gly904Glu	chr13:32911203	c.2711G>A	Missense	VUS	Negative	Negative
P1465	Female	75	Asian	PDAC	*ERBB2*	p.Glu40Lys	chr17:37863287	c.118G>A	Missense	Novel	Negative	Negative
P1473	Male	55	Asian	PDAC	*PNLIPRP2*	p.Gly276Ser	chr10:118394360	c.828G>A	Missense	Novel	Negative	Negative
P1473	Male	55	Asian	PDAC	*BRCA2*	p.Gln1502Arg	chr13:32912997	c.4505A>G	Missense	VUS	Negative	Negative
P1475	Female	55	Asian	Ampullary cancer	*BRCA2*	p.Ser1479Asn	chr13:32912928	c.4436G>A	Missense	Novel	Negative	Negative
P1477	Female	58	Asian	PDAC	*SPINK1*	p.Lys31fs	chr5:147207677	c.93_101delATGTTACAA	Frameshift	Novel	Negative	Negative
P1492	Female	56	Asian	PDAC	*RECQL4*	p.Gly351Ser	chr8:145741452	c.1051G>A	Missense	Novel	Negative	Negative
P1501	Male	65	Asian	PDAC	*STK11*	p.Arg147His	chr19:1219388	c.440G>A	Missense	VUS	Negative	Father with gastric cancer
P1504	Female	61	Asian	PDAC	*STK11*	p.Arg211Trp	chr19:1220613	c.631C>T	Missense	VUS	Gastric cancer	Negative

© 2022 Yin L et al. *JAMA Network Open*.

P1508	Male	69	Asian	PDAC	*PALB2*	p.Cys520fs	chr16:23646309	c.1557_1558insA	Frameshift	Novel	Negative	Negative
P1509	Male	63	Asian	PDAC	*PNLIPRP2*	p.Thr448Asn	chr10:118401790	c.1345C>A	Missense	Novel	Negative	Negative
P1515	Male	67	Asian	PDAC	*BUB1B*	p.Val551fs	chr15:40494811	c.1650_1651insC	Frameshift	Novel	Negative	Negative
P1516	Male	67	Asian	PDAC	*CDH1*	p.Gly434Val	chr16:68847379	c.1301G>T	Missense	Novel	Negative	Negative
P1516	Male	67	Asian	PDAC	*CPB1*	p.Phe232fs	chr3:148562469	c.693_694insC	Frameshift	Novel	Negative	Negative
P1517	Male	55	Asian	PDAC	*BRCA1*	p.Glu1053Asp	chr17:41244389	c.3159A>C	Missense	VUS	Negative	Negative
P1517	Male	55	Asian	PDAC	*MSH6*	p.Asn1327Ser	chr2:48033769	c.3980A>G	Missense	VUS	Negative	Negative
P1519	Male	54	Asian	PDAC	*BAP1*	p.Ile131fs	chr3:52441461	c.390_391insC	Frameshift	Novel	Negative	Negative
P1521	Male	57	Asian	PDAC	*ERCC4*	p.Ile634Val	chr16:14031711	c.1900A>G	Missense	Novel	Negative	Negative
P1523	Female	47	Asian	PDAC	*FANCA*	p.Gly122fs	chr16:89877397	c.365delG	Frameshift	Novel	Negative	Negative
P1523	Female	47	Asian	PDAC	*TNS3*	p.Phe1134Leu	chr7:47342605	c.3400T>C	Missense	Novel	Negative	Negative
P1523	Female	47	Asian	PDAC	*RECQL4*	p.Tyr519fs	chr8:145740384	c.1555_1556insC	Frameshift	Novel	Negative	Negative
P1529	Female	61	Asian	PDAC	*ARID1A*	p.Pro1326fs	chr1:27100181	c.4001_4002insGCAGCA	Frameshift	Novel	Negative	Negative
P1530	Female	63	Asian	PDAC	*ARID1A*	p.Gly2040Arg	chr1:27106507	c.6118G>A	Missense	Novel	Negative	Negative
P1537	Male	62	Asian	PDAC	*PNLIPRP2*	p.Ile78Thr	chr10:118385485	c.235T>C	Missense	Novel	Negative	Negative
P1537	Male	62	Asian	PDAC	*BUB1B*	p.Asp547fs	chr15:40494799	c.1638_1639insC	Frameshift	Novel	Negative	Negative
P1540	Female	66	Asian	PDAC	*PNLIPRP2*	p.Phe345fs	chr10:118396388	c.1036delT	Frameshift	Novel	Negative	Negative
P1543	Male	76	Asian	PDAC	*BAP1*	p.Leu334fs	chr3:52439240	c.1001_1002insCCT	Frameshift	Novel	Negative	Negative
P1554	Male	43	Asian	PDAC	*BRCA1*	p.Thr1119Ser	chr17:41244192	c.3356C>G	Missense	VUS	Negative	Negative
P1564	Male	58	Asian	PDAC	*CTRC*	p.Arg83Pro	chr1:15768960	c.248G>C	Missense	Novel	Negative	Negative
P1565	Male	47	Asian	PDAC	*ATM*	p.Gln95Lys	chr11:108100002	c.283C>A	Missense	VUS	Negative	Negative
P1572	Male	58	Asian	PDAC	*MLH1*	p.Gly232Arg	chr3:37055939	c.694G>C	Missense	Novel	Negative	Negative
P1573	Male	65	Asian	PDAC	*ATM*	p.Asp1467Glu	chr11:108160493	c.4401C>G	Missense	Novel	Negative	Negative
P1574	Male	68	Asian	PDAC	*RNF43*	p.Leu647fs	chr17:56435197	c.1939_1940insA	Frameshift	Novel	Negative	Negative
P1577	Male	70	Asian	PDAC	*BRCA2*	p.Glu3316fs	chr13:32972594	c.9946_9947delGA	Frameshift	Novel	Negative	Negative
P1583	Female	66	Asian	PDAC	*ATM*	p.Thr72Ala	chr11:108099933	c.214A>G	Missense	Novel	Negative	Negative
P1583	Female	66	Asian	PDAC	*TERT*	p.Ala437fs	chr5:1293693	c.1307_1308insC	Frameshift	Novel	Negative	Negative
P1595	Female	62	Asian	PDAC	*NOC2L*	p.Ala179Gly	chr1:891546	c.536C>G	Missense	Novel	Negative	Negative
P1604	Female	49	Asian	PNET	*BRCA1*	p.Phe1498Tyr	chr17:41228559	c.4493T>A	Missense	Novel	Negative	Mother with gastric cancer

© 2022 Yin L et al. *JAMA Network Open*.

P1604	Female	49	Asian	PNET	*RNF43*	p.Arg554Trp	chr17:56435477	c.1660C>T	Missense	Novel	Negative	Mother with gastic cancer
P1606	Female	76	Asian	PDAC	*CTRC*	p.Thr58Met	chr1:15767029	c.173C>T	Missense	Novel	Negative	Negative
P1608	Female	77	Asian	PDAC	*MLH1*	p.Val213Leu	chr3:37053550	c.637G>T	Missense	VUS	Negative	Negative
P1611	Male	66	Asian	PDAC	*ARID1A*	p.Gln1358Pro	chr1:27100361	c.4073A>C	Missense	Novel	Negative	Negative
P1611	Male	66	Asian	PDAC	*BRCA2*	p.Leu1059Met	chr13:32911667	c.3175C>A	Missense	Novel	Negative	Negative
P1611	Male	66	Asian	PDAC	*BRCA2*	p.Thr2564Ile	chr13:32931952	c.7691C>T	Missense	VUS	Negative	Negative
P1612	Male	74	Asian	PDAC	*CDKN2A*	p.Pro41Gln	chr9:21974705	c.122C>A	Missense	VUS	Negative	Negative
P1620	Male	47	Asian	PDAC	*ATM*	p.Asp7fs	chr11:108098367	c.19_21delGAT	Frameshift	Novel	Negative	Negative
P1643	Male	59	Asian	PDAC	*CLPS*	p.Thr70Met	chr6:35763053	c.209C>T	Missense	Novel	Negative	Negative
P1644	Male	65	Asian	PDAC	*ATM*	p.Ile2865Val	chr11:108218014	c.8593A>G	Missense	VUS	Negative	Negative
P1644	Male	65	Asian	PDAC	*MSH6*	p.Tyr397Cys	chr2:48026312	c.1190A>G	Missense	VUS	Negative	Negative
P1646	Male	61	Asian	PDAC	*CPA1*	p.Arg240Gln	chr7:130024399	c.719G>A	Missense	Novel	Negative	Negative
P1650	Male	43	Asian	PDAC	*CFTR*	p.Gly1237Asp	chr7:117267817	c.3710G>A	Missense	VUS	Negative	Negative
P1652	Female	72	Asian	PDAC	*FANCA*	p.Cys1159Ser	chr16:89813029	c.3476G>C	Missense	VUS	Negative	Father with gastric cancer, mother with colon cancer
P1655	Male	34	Asian	PDAC	*PNLIPRP2*	p.Ala145Val	chr10:118386478	c.436C>T	Missense	Novel	Negative	Negative
P1655	Male	34	Asian	PDAC	*WEE1*	p.Met267fs	chr11:9597791	c.797_798insT	Frameshift	Novel	Negative	Negative
P1656	Male	54	Asian	PDAC	*PNLIPRP2*	p.Ala305Val	chr10:118394448	c.916C>T	Missense	Novel	Negative	Negative
P1657	Male	56	Asian	PDAC	*STK11*	p.Arg211Trp	chr19:1220613	c.631C>T	Missense	VUS	Negative	Negative
P1659	Male	51	Asian	PDAC	*ARID1A*	p.Ala163fs	chr1:27023380	c.494_502delCGGCCGCCG	Frameshift	Novel	Negative	Negative
P1661	Male	59	Asian	PDAC	*CTRB2*	p.Gln220Ter	chr16:75238193	c.658C>T	Nonsense	Novel	Negative	Father with gastric cancer
P1662	Female	48	Asian	PDAC	*PLA2G1B*	p.Val24Met	chr12:120763788	c.70G>A	Missense	Novel	Negative	Negative
P1663	Female	59	Asian	PDAC	*CTRB1*	p.Gly18Arg	chr16:75252957	c.52G>C	Missense	Novel	Negative	Negative
P1664	Female	64	Asian	PDAC	*PNLIPRP2*	p.Gly276Ser	chr10:118394360	c.828G>A	Missense	Novel	Negative	Negative
P1665	Male	64	Asian	PDAC	*ATM*	p.Lys385Glu	chr11:108119747	c.1153A>G	Missense	Novel	Negative	Negative
P1666	Female	48	Asian	PDAC	*RECQL4*	p.Ser445fs	chr8:145740767	c.1332_1333insC	Frameshift	Novel	Negative	Negative

© 2022 Yin L et al. *JAMA Network Open*.

P1671	Male	78	Asian	PDAC	*PALB2*	p.Asp1141Asn	chr16:23614920	c.3421G>A	Missense	VUS	Negative	Brother with lung cancer
P1679	Male	56	Asian	PDAC	*ATM*	p.Gly2287Arg	chr11:108196836	c.6859G>A	Missense	VUS	Negative	Negative
P1679	Male	56	Asian	PDAC	*ERBB2*	p.Val308Met	chr17:37868201	c.922G>A	Missense	Novel	Negative	Negative
P1680	Male	56	Asian	PDAC	*FANCA*	p.Pro799Ser	chr16:89836354	c.2395C>T	Missense	VUS	Negative	Negative
P1681	Male	66	Asian	PDAC	*STK11*	p.Ala273Val	chr19:1221295	c.818C>T	Missense	Novel	Negative	Negative
P1683	Female	47	Asian	PDAC	*STK11*	p.Arg384Gln	chr19:1226495	c.1151G>A	Missense	VUS	Negative	Negative
P1683	Female	47	Asian	PDAC	*BUB1*	p.Tyr853Cys	chr2:111399286	c.2558A>G	Missense	Novel	Negative	Negative
P1693	Female	50	Asian	PDAC	*GRP*	p.Gln70fs	chr18:56892793	c.209_210delAG	Frameshift	Novel	Negative	Negative
P1694	Male	76	Asian	PDAC	*CPB1*	p.Tyr197His	chr3:148562277	c.589T>C	Missense	Novel	Negative	Negative
P1694	Male	76	Asian	PDAC	*CLPS*	p.Thr85Asn	chr6:35763008	c.254C>A	Missense	Novel	Negative	Negative
P1695	Male	43	Asian	PDAC	*MSH6*	p.Pro1073Arg	chr2:48030604	c.3218C>G	Missense	VUS	Negative	Negative
P1696	Male	49	Asian	PNET	*TERT*	p.Arg1097fs	chr5:1254489	c.3288_3289insG	Frameshift	Novel	Negative	Negative
P1704	Female	59	Asian	PDAC	*TNS3*	p.Arg1365Lys	chr7:47323298	c.4094G>A	Missense	Novel	Negative	Negative
P1706	Male	59	Asian	PDAC	*MLH1*	p.Leu266Arg	chr3:37059003	c.797T>G	Missense	VUS	Negative	Negative
P1707	Male	76	Asian	PDAC	*MSH6*	p.Lys804Thr	chr2:48027533	c.2411A>C	Missense	VUS	Negative	Negative
P1708	Male	43	Asian	PDAC	*ARID1A*	p.Tyr430Cys	chr1:27056293	c.1289A>G	Missense	Novel	Negative	Negative
P1711	Male	67	Asian	PDAC	*CELA3A*	p.His187fs	chr1:22333926	c.560_561insG	Frameshift	Novel	Negative	Negative
P1711	Male	67	Asian	PDAC	*FANCA*	p.Arg661fs	chr16:89839713	c.1979_1980insC	Frameshift	Novel	Negative	Negative
P1712	Female	52	Asian	PDAC	*CELA3B*	p.Tyr81Ter	chr1:22307546	c.243C>A	Nonsense	Novel	Negative	Negative
P1717	Male	71	Asian	PDAC	*AMY2A*	p.Pro49Leu	chr1:104160208	c.146C>T	Missense	Novel	Negative	Negative
P1717	Male	71	Asian	PDAC	*ATM*	p.Thr2853Met	chr11:108216609	c.8558C>T	Missense	VUS	Negative	Negative
P1719	Female	58	Asian	PDAC	*ERCC4*	p.Arg483fs	chr16:14029234	c.1448_1450delGAA	Frameshift	Novel	Negative	Negative
P1720	Female	71	Asian	PDAC	*ERCC4*	p.Glu17Ala	chr16:14014072	c.50A>C	Missense	Novel	Negative	Negative
P1720	Female	71	Asian	PDAC	*BRIP1*	p.Lys752Gln	chr17:59821796	c.2254A>C	Missense	VUS	Negative	Negative
P1722	Male	49	Asian	PDAC	*ATM*	p.Ile2185Thr	chr11:108192129	c.6554T>C	Missense	VUS	Negative	Negative
P1722	Male	49	Asian	PDAC	*RECQL4*	p.Arg125fs	chr8:145742131	c.371_372insGC	Frameshift	Novel	Negative	Negative
P1726	Female	60	Asian	PDAC	*WEE1*	p.Ile550Ser	chr11:9608265	c.1649T>G	Missense	Novel	Negative	Negative
P1727	Female	45	Asian	PDAC	*FANCC*	p.Thr503Met	chr9:97869373	c.1508C>T	Missense	VUS	Negative	Negative
P1729	Male	46	Asian	PDAC	*CELA3A*	p.Trp193Ter	chr1:22333945	c.579G>A	Nonsense	Novel	Negative	Negative
P1731	Male	60	Asian	PDAC	*RAD51C*	p.Thr78Ile	chr17:56772379	c.233C>T	Missense	VUS	Negative	Negative
P1732	Male	61	Asian	PDAC	*BUB1*	p.Gln144fs	chr2:111428138	c.429_430insT	Frameshift	Novel	Negative	Negative

© 2022 Yin L et al. *JAMA Network Open*.

P1732	Male	61	Asian	PDAC	*TERT*	p.Thr1098fs	chr5:1254485	c.3292_3293insG	Frameshift	Novel	Negative	Negative
P1732	Male	61	Asian	PDAC	*RECQL4*	p.Leu828Gln	chr8:145738502	c.2483T>A	Missense	Novel	Negative	Negative
P1739	Male	17	Asian	Acute pancreatitis	*ATM*	p.Leu606Phe	chr11:108123557	c.1816C>T	Missense	Novel	Negative	Negative
P1740	Female	55	Asian	Acute pancreatitis	*ERCC4*	p.Arg468fs	chr16:14029187	c.1402_1403insA	Frameshift	Novel	Negative	Negative
P1740	Female	55	Asian	Acute pancreatitis	*MSH6*	p.Arg721Gly	chr2:48027283	c.2161A>G	Missense	VUS	Negative	Negative
P1745	Female	80	Asian	Acute pancreatitis	*PALB2*	p.Arg663Cys	chr16:23641488	c.1987C>T	Missense	VUS	Negative	Negative
P1747	Male	44	Asian	Acute pancreatitis	*CFTR*	p.Ile507Asn	chr7:117199645	c.1520T>A	Missense	Novel	Negative	Negative
P1749	Male	39	Asian	Acute pancreatitis	*NOC2L*	p.Thr616fs	chr1:881625	c.1844_1845insT	Frameshift	Novel	Negative	Negative
P1765	Female	81	Asian	Acute pancreatitis	*PALB2*	p.Ala245Ser	chr16:23647134	c.733G>T	Missense	Novel	Negative	Negative
P1766	Male	65	Asian	Acute pancreatitis	*CPB1*	p.Arg152Cys	chr3:148558742	c.454C>T	Missense	Novel	Negative	Negative
P1769	Female	58	Asian	Acute pancreatitis	*ARID1A*	p.Leu2053fs	chr1:27106545	c.6156_6157insA	Frameshift	Novel	Negative	Negative
P1769	Female	58	Asian	Acute pancreatitis	*ARID1A*	p.Asp1975fs	chr1:27106310	c.5923delG	Frameshift	Novel	Negative	Negative
P1775	Male	21	Asian	Acute pancreatitis	*MSH6*	p.Thr1175fs	chr2:48032133	c.3523_3525delACT	Frameshift	Novel	Negative	Negative
P1776	Female	60	Asian	Acute pancreatitis	*ARID1A*	p.Arg727Gln	chr1:27087893	c.2180G>A	Missense	Novel	Negative	Negative
P1776	Female	60	Asian	Acute pancreatitis	*BUB1*	p.Glu192fs	chr2:111425418	c.575_576insT	Frameshift	Novel	Negative	Negative
P1778	Male	70	Asian	Chronic pancreatitis	*CFTR*	p.Ile371Thr	chr7:117180396	c.1112T>C	Missense	Novel	Negative	Negative
P1787	Male	35	Asian	Acute pancreatitis	*ERBB2*	p.Trp482Arg	chr17:37872123	c.1444T>C	Missense	Novel	Negative	Negative
P1793	Male	49	Asian	Acute pancreatitis	*BRCA2*	p.Asn404Ser	chr13:32906826	c.1211A>G	Missense	VUS	Negative	Negative
P1793	Male	49	Asian	Acute pancreatitis	*CPA1*	p.Arg27Gln	chr7:130020953	c.80G>A	Missense	Novel	Negative	Negative
P1801	Male	60	Asian	Acute pancreatitis	*CELA2A*	p.Val196Met	chr1:15792586	c.586G>A	Missense	Novel	Negative	Negative
P1805	Male	90	Asian	Acute pancreatitis	*RNF43*	p.Arg668Trp	chr17:56435135	c.2002C>T	Missense	Novel	Negative	Negative
P1808	Male	52	Asian	Acute pancreatitis	*ATM*	p.Asp840Gly	chr11:108137950	c.2519A>G	Missense	Novel	Negative	Negative
P1808	Male	52	Asian	Acute pancreatitis	*PLA2G1B*	p.Gly55fs	chr12:120763693	c.164delG	Frameshift	Novel	Negative	Negative

© 2022 Yin L et al. *JAMA Network Open*.

P1821	Male	54	Asian	Acute pancreatitis	*ARID1A*	p.His1581fs	chr1:27101457	c.4739_4740insC	Frameshift	Novel	Negative	Negative
P1821	Male	54	Asian	Acute pancreatitis	*CDKN2A*	p.Gly45Arg	chr9:21974694	c.133G>C	Missense	VUS	Negative	Negative
P1829	Male	48	Asian	PNET	*ATM*	p.Pro1112Ser	chr11:108150267	c.3334C>T	Missense	VUS	Negative	Negative
P1831	Male	68	Asian	PDAC	*FANCC*	p.Val379Gly	chr9:97876929	c.1136T>G	Missense	Novel	Negative	Negative
P1838	Male	57	Asian	PDAC	*BRCA2*	p.Ser1442Leu	chr13:32912817	c.4325C>T	Missense	Novel	Negative	Negative
P1838	Male	57	Asian	PDAC	*FANCA*	p.Cys1159Ser	chr16:89813029	c.3476G>C	Missense	VUS	Negative	Negative
P1839	Male	58	Asian	Acute pancreatitis	*RECQL4*	p.Val678Met	chr8:145739338	c.2032G>A	Missense	VUS	Negative	Negative
P1840	Male	52	Asian	Duodenal carcinoma	*BRIP1*	p.Lys995Glu	chr17:59761424	c.2983A>G	Missense	VUS	Negative	Negative
P1840	Male	52	Asian	Duodenal carcinoma	*BRIP1*	p.Arg419Gln	chr17:59876545	c.1256G>A	Missense	VUS	Negative	Negative
P1841	Female	42	Asian	Acute pancreatitis	*ATM*	p.Asp2889Val	chr11:108218087	c.8666A>T	Missense	Novel	Negative	Negative
P1844	Male	49	Asian	SCN	*NOC2L*	p.Leu412Phe	chr1:887477	c.1234C>T	Missense	Novel	Negative	Negative
P1849	Male	82	Asian	Acute pancreatitis	*RAD51C*	p.Arg370Ter	chr17:56811560	c.1108C>T	Nonsense	VUS	Negative	Negative
P1852	Male	11	Asian	Acute pancreatitis	*BUB1B*	p.Ser788fs	chr15:40502386	c.2363_2364delCT	Frameshift	Novel	Negative	Negative
P1864	Female	67	Asian	IPMN	*CTRB1*	p.Gly18Arg	chr16:75252957	c.52G>C	Missense	Novel	Negative	Negative
P1865	Male	75	Asian	Chronic pancreatitis	*RECQL4*	p.Ala550Glu	chr8:145739881	c.1649C>A	Missense	Novel	Negative	Negative
P1867	Male	44	Asian	Chronic pancreatitis	*CPA1*	p.Arg169Cys	chr7:130023253	c.505C>T	Missense	Novel	Negative	Negative
P1871	Male	59	Asian	Ampullary cancer	*CPA1*	p.Glu328Ala	chr7:130025182	c.983A>C	Missense	Novel	Negative	Negative
P1880	Female	34	Asian	SPT	*CEL*	p.Ala489Gly	chr9:135946018	c.1466C>G	Missense	Novel	Negative	Negative
P1886	Male	61	Asian	Chronic pancreatitis	*PMS2*	p.Lys581Glu	chr7:6026655	c.1741A>G	Missense	Novel	Negative	Negative
P1890	Female	26	Asian	GIST	*BRCA1*	p.Gly1319Ser	chr17:41243593	c.3955G>A	Missense	VUS	Negative	Negative
P1892	Male	76	Asian	Chronic pancreatitis	*CLPS*	p.Arg22Gln	chr6:35765000	c.65G>A	Missense	Novel	Negative	Negative
P1907	Female	42	Asian	Acute pancreatitis	*ATM*	p.Ser2165Pro	chr11:108192068	c.6493T>C	Missense	Novel	Negative	Negative
P1910	Male	62	Asian	IPMN	*RAD51B*	p.Thr256Met	chr14:68758611	c.767C>T	Missense	Novel	Negative	Negative
P1918	Female	37	Asian	MCN	*MSH6*	p.Ala53Asp	chr2:48010530	c.158C>A	Missense	VUS	Negative	Negative
P1918	Female	37	Asian	MCN	*MSH6*	p.Gly56fs	chr2:48010538	c.177_178insAGGCCC	Frameshift	VUS	Negative	Negative
P1918	Female	37	Asian	MCN	*ATM*	p.Glu2818Ala	chr11:108216504	c.8453A>C	Missense	Novel	Negative	Negative

© 2022 Yin L et al. *JAMA Network Open*.

P1930	Male	58	Asian	Chronic pancreatitis	*CTRB2*	p.Leu217Pro	chr16:75238201	c.650T>C	Missense	Novel	Negative	Negative
P1945	Female	69	Asian	PDAC	*ATM*	p.His943Arg	chr11:108139326	c.2828A>G	Missense	Novel	Colon cancer	Negative
P1946	Male	54	Asian	IPMN	*FANCA*	p.Pro799Ser	chr16:89836354	c.2395C>T	Missense	VUS	Negative	Negative
P1951	Male	57	Asian	SCN	*TNS3*	p.Ile1183Val	chr7:47336809	c.3547A>G	Missense	Novel	Negative	Negative
P1956	Male	47	Asian	Chronic pancreatitis	*BRCA2*	p.Lys1765Gln	chr13:32913785	c.5293A>C	Missense	Novel	Negative	Negative
P1967	Male	47	Asian	Cholangiocarcinoma	*GRP*	p.Arg141fs	chr18:56897667	c.422_425delGGAA	Frameshift	Novel	Negative	Negative
P1980	Male	63	Asian	Cholangiocarcinoma	*STK11*	p.Phe264Leu	chr19:1221269	c.792T>A	Missense	Novel	Negative	Negative
P1991	Female	44	Asian	Duodenal carcinoma	*BRCA2*	p.Glu2420fs	chr13:32929248	c.7259_7260insGAA	Frameshift	Novel	Negative	Negative
P1996	Female	47	Asian	Ampullary cancer	*MLH1*	p.His518Arg	chr3:37070418	c.1553A>G	Missense	VUS	Negative	Negative
P2001	Male	36	Asian	Chronic pancreatitis	*BRIP1*	p.Asn775Ser	chr17:59820429	c.2324A>G	Missense	VUS	Negative	Negative
P2002	Female	59	Asian	SCN	*ARID1A*	p.Lys1007fs	chr1:27094312	c.3020_3021insC	Frameshift	Novel	Negative	Negative
P2002	Female	59	Asian	SCN	*BRCA1*	p.Ser1009Pro	chr17:41244523	c.3025T>C	Missense	Novel	Negative	Negative
P2002	Female	59	Asian	SCN	*CFTR*	p.Leu1414fs	chr7:117305612	c.4241delT	Frameshift	Novel	Negative	Negative
P2003	Female	52	Asian	SCN	*ARID1A*	p.Leu1831Pro	chr1:27105881	c.5492T>C	Missense	Novel	Colon polyp	Negative
P2004	Male	68	Asian	GIST	*ERCC4*	p.Ile634Val	chr16:14031711	c.1900A>G	Missense	Novel	Lung cancer	Negative
P2007	Male	72	Asian	MCN	*ARID1A*	p.Leu2170Pro	chr1:27106898	c.6509T>C	Missense	Novel	Negative	Negative
P2009	Male	74	Asian	Acute pancreatitis	*BRCA1*	p.Ala1248Val	chr17:41243805	c.3743C>T	Missense	Novel	Negative	Negative
P2010	Male	85	Asian	Acute pancreatitis	*ERBB2*	p.Val725Met	chr17:37879878	c.2173G>A	Missense	Novel	Negative	Negative
P2011	Male	56	Asian	Acute pancreatitis	*BRCA2*	p.Lys457Thr	chr13:32906985	c.1370A>C	Missense	Novel	Negative	Negative
P2011	Male	56	Asian	Acute pancreatitis	*BRCA2*	p.Pro458Leu	chr13:32906988	c.1373C>T	Missense	Novel	Negative	Negative
P2011	Male	56	Asian	Acute pancreatitis	*BRCA2*	p.Leu459Ile	chr13:32906990	c.1375T>A	Missense	Novel	Negative	Negative
P2011	Male	56	Asian	Acute pancreatitis	*CPA2*	p.Ser364Gly	chr7:129929417	c.1090A>G	Missense	Novel	Negative	Negative
P2011	Male	56	Asian	Acute pancreatitis	*ERBB2*	p.Leu455fs	chr17:37872037	c.1362delG	Frameshift	Novel	Negative	Negative
P2012	Male	58	Asian	Ampullary cancer	*ARID1A*	p.Ser662Gly	chr1:27087410	c.1984A>G	Missense	Novel	Negative	Negative
P2012	Male	58	Asian	Ampullary cancer	*ATM*	p.Lys1253Asn	chr11:108154966	c.3759G>T	Missense	Novel	Negative	Negative

© 2022 Yin L et al. *JAMA Network Open*.

P2012	Male	58	Asian	Ampullary cancer	*BRCA1*	p.Thr900Asn	chr17:41244849	c.2699C>A	Missense	Novel	Negative	Negative
P2012	Male	58	Asian	Ampullary cancer	*PNLIPRP2*	p.Thr448Asn	chr10:118401790	c.1345C>A	Missense	Novel	Negative	Negative
P2015	Male	66	Asian	Acute pancreatitis	*FANCC*	p.Leu375Pro	chr9:97876941	c.1124T>C	Missense	Novel	Negative	Negative
P2016	Female	36	Asian	IPMN	*BRCA2*	p.Glu13Gly	chr13:32890635	c.38A>G	Missense	Novel	Negative	Negative
P2016	Female	36	Asian	IPMN	*HNF1B*	p.Glu102Gly	chr17:36104571	c.305A>G	Missense	Novel	Negative	Negative
P2016	Female	36	Asian	IPMN	*MLH1*	p.Lys618fs	chr3:37089130	c.1852_1853delAA	Frameshift	Novel	Negative	Negative
P2020	Male	74	Asian	PNET	*MSH2*	p.Gly195Arg	chr2:47637449	c.583G>A	Missense	Novel	Negative	Negative
P2021	Female	26	Asian	Acute pancreatitis	*ARID1A*	p.Leu1812Pro	chr1:27105824	c.5435T>C	Missense	Novel	Negative	Negative
P2024	Female	63	Asian	Acute pancreatitis	*BRCA1*	p.Glu1282Lys	chr17:41243704	c.3844G>A	Missense	Novel	Negative	Negative
P2024	Female	63	Asian	Acute pancreatitis	*CFTR*	p.Arg734Gly	chr7:117232421	c.2200A>G	Missense	Novel	Negative	Negative
P2026	Female	66	Asian	Acute pancreatitis	*MSH6*	p.Ser346Cys	chr2:48026159	c.1037C>G	Missense	VUS	Negative	Negative
P2029	Female	50	Asian	Acute pancreatitis	*TERT*	p.Gly715Asp	chr5:1278898	c.2144G>A	Missense	Novel	Negative	Negative
P2030	Female	44	Asian	GIST	*BRCA1*	p.Ser590fs	chr17:41245776	c.1769_1771delGTA	Frameshift	Novel	Negative	Negative
P2030	Female	44	Asian	GIST	*BRCA2*	p.Asp2680Asn	chr13:32937377	c.8038G>A	Missense	Novel	Negative	Negative
P2032	Male	64	Asian	Duodenal carcinoma	*MSH6*	p.Glu493Gly	chr2:48026600	c.1478A>G	Missense	Novel	Colon polyp	Mother with gastric cancer, brother with colon cancer
P2036	Female	53	Asian	Acute pancreatitis	*BRIP1*	p.Glu938Gly	chr17:59763289	c.2813A>G	Missense	Novel	Negative	Negative
P2037	Male	45	Asian	Acute pancreatitis	*BAP1*	p.Ser628Ala	chr3:52437162	c.1882T>G	Missense	Novel	Negative	Negative
P2040	Female	53	Asian	PNET	*ATM*	p.Tyr264Cys	chr11:108115643	c.791A>G	Missense	VUS	Negative	Negative
P2040	Female	53	Asian	PNET	*NOC2L*	p.Thr327fs	chr1:888578	c.978_979insGC	Frameshift	Novel	Negative	Negative
P2042	Female	24	Asian	SPT	*BRIP1*	p.Asn643Ile	chr17:59857629	c.1928A>T	Missense	Novel	Negative	Negative
P2042	Female	24	Asian	SPT	*HNF1B*	p.Gln521His	chr17:36059172	c.1563G>C	Missense	Novel	Negative	Negative
P2045	Male	49	Asian	Acute pancreatitis	*ATM*	p.Trp164Arg	chr11:108106555	c.490T>A	Missense	Novel	Negative	Negative
P2045	Male	49	Asian	Acute pancreatitis	*HNF1B*	p.Pro412fs	chr17:36065027	c.1235delC	Frameshift	Novel	Negative	Negative

© 2022 Yin L et al. *JAMA Network Open*.

P2046	Female	48	Asian	SCN	*BRCA2*	p.Ser3303Asn	chr13:32972558	c.9908G>A	Missense	VUS	Negative	Negative
P2054	Male	49	Asian	Ampullary cancer	*BRCA2*	p.Val2610Leu	chr13:32936682	c.7828G>C	Missense	Novel	Negative	Negative
P2055	Male	62	Asian	Cholangiocarcinoma	*BRCA2*	p.Ser636Pro	chr13:32907521	c.1906T>C	Missense	Novel	Negative	Negative
P2057	Female	76	Asian	Cholangiocarcinoma	*BRIP1*	p.Lys966Glu	chr17:59763206	c.2896A>G	Missense	Novel	Negative	Negative
P2057	Female	76	Asian	Cholangiocarcinoma	*CFTR*	p.Ser737fs	chr7:117232429	c.2208_2209insC	Frameshift	Novel	Negative	Negative
P2058	Male	38	Asian	Acute pancreatitis	*ARID1A*	p.Gln1327fs	chr1:27100175	c.3979_3980insC	Frameshift	Novel	Negative	Negative
P2058	Male	38	Asian	Acute pancreatitis	*BAP1*	p.Arg512fs	chr3:52437628	c.1532_1533insTC	Frameshift	Novel	Negative	Negative
P2058	Male	38	Asian	Acute pancreatitis	*TNS3*	p.Arg455Cys	chr7:47408880	c.1363C>T	Missense	Novel	Negative	Negative
P2061	Male	66	Asian	Duodenal carcinoma	*CDH1*	p.Asn637Ser	chr16:68856102	c.1910A>G	Missense	Novel	Negative	Negative
P2068	Female	13	Asian	SPT	*PNLIP*	p.Ala433fs	chr10:118321108	c.1296_1297delAG	Frameshift	Novel	Negative	Negative
P2069	Male	57	Asian	PDAC	*CLPS*	p.Arg22Gln	chr6:35765000	c.65G>A	Missense	Novel	Negative	Negative
P2069	Male	57	Asian	PDAC	*CPA2*	p.Val137fs	chr7:129912938	c.407_408insA	Frameshift	Novel	Negative	Negative
P2071	Male	57	Asian	Acute pancreatitis	*CPA2*	p.Glu380Gly	chr7:129929466	c.1139A>G	Missense	Novel	Negative	Negative
P2075	Female	65	Asian	SCN	*ARID1A*	p.Asp1193Gly	chr1:27099341	c.3578A>G	Missense	Novel	Negative	Negative
P2075	Female	65	Asian	SCN	*ARID1A*	p.Gln1327fs	chr1:27100183	c.3980_3984delAGCAG	Frameshift	Novel	Negative	Negative
P2075	Female	65	Asian	SCN	*CFTR*	p.Leu1011fs	chr7:117250612	c.3032delT	Frameshift	Novel	Negative	Negative
P2076	Female	70	Asian	SCN	*CDH1*	p.Lys381Glu	chr16:68847219	c.1141A>G	Missense	Novel	Negative	Negative
P2077	Male	54	Asian	Chronic pancreatitis	*IDO2*	p.Lys229fs	chr8:39847334	c.684_685delCA	Frameshift	Novel	Negative	Father with gastric cancer
P2077	Male	54	Asian	Chronic pancreatitis	*MLH1*	p.Ser95Ala	chr3:37042521	c.283T>G	Missense	VUS	Negative	Father with gastric cancer
P2079	Male	21	Asian	SPT	*RAD51C*	p.Gly98Ser	chr17:56772438	c.292G>A	Missense	Novel	Negative	Negative
P2084	Female	59	Asian	PDAC	*ATM*	p.Leu254Val	chr11:108115612	c.760T>G	Missense	Novel	Negative	Negative
P2084	Female	59	Asian	PDAC	*CEL*	p.Val197Ala	chr9:135941959	c.590T>C	Missense	Novel	Negative	Negative
P2086	Female	40	Asian	PDAC	*BRCA2*	p.Thr665Ile	chr13:32910486	c.1994C>T	Missense	Novel	Negative	Negative
P2086	Female	40	Asian	PDAC	*BUB1B*	p.Glu538fs	chr15:40494648	c.1610_1611insT	Frameshift	Novel	Negative	Negative
P2088	Female	32	Asian	PNET	*ARID1A*	p.Glu1006Gly	chr1:27094309	c.3017A>G	Missense	Novel	Negative	Negative
P2089	Male	64	Asian	PNET	*TP53*	p.Lys132Arg	chr17:7578535	c.395A>G	Missense	VUS	Negative	Negative

© 2022 Yin L et al. *JAMA Network Open*.

P2093	Female	64	Asian	Chronic pancreatitis	*RECQL4*	p.Ala919Thr	chr8:145738230	c.2755G>A	Missense	VUS	Negative	Negative
P2102	Female	47	Asian	MCN	*BRIP1*	p.Ile552Val	chr17:59858341	c.1654A>G	Missense	VUS	Negative	Negative
P2104	Female	53	Asian	SCN	*MLH1*	p.Arg497Trp	chr3:37070354	c.1489C>T	Missense	VUS	Negative	Negative
P2139	Male	62	Asian	Acute pancreatitis	*ARID1A*	p.Gln1411fs	chr1:27100948	c.4231_4236delCAGCCT	Frameshift	Novel	Negative	Negative
P2142	Male	75	Asian	Cholangiocarcinoma	*FANCA*	p.Pro799Ser	chr16:89836354	c.2395C>T	Missense	VUS	Negative	Negative
P2151	Male	61	Asian	PNET	*CTRC*	p.Thr58Met	chr1:15767029	c.173C>T	Missense	Novel	Negative	Negative
P2151	Male	61	Asian	PNET	*CDKN2A*	p.Val115Glu	chr9:21971014	c.344T>A	Missense	VUS	Negative	Negative
P2159	Male	76	Asian	IPMN	*BRCA2*	p.Asp2438Asn	chr13:32929302	c.7312G>A	Missense	Novel	Negative	Negative
P2164	Male	35	Asian	PNET	*FANCL*	p.Asp213Asn	chr2:58392928	c.637G>A	Missense	VUS	Negative	Negative
P2164	Male	35	Asian	PNET	*TNS3*	p.Ser383fs	chr7:47409086	c.1148_1156delGTGACCACA	Frameshift	Novel	Negative	Negative
P2165	Male	57	Asian	Duodenal carcinoma	*PMS2*	p.Arg295Trp	chr7:6035185	c.883C>T	Missense	VUS	Negative	Negative
P2168	Female	72	Asian	Ampullary cancer	*CELA3A*	p.Pro156His	chr1:22333475	c.467C>A	Missense	Novel	Negative	Negative
P2176	Female	47	Asian	PNET	*BRCA2*	p.Asn1805Asp	chr13:32913905	c.5413A>G	Missense	VUS	Negative	Negative
P2181	Male	79	Asian	GIST	*MLH1*	p.Leu259Ser	chr3:37056021	c.776T>C	Missense	VUS	Negative	Negative
P2182	Male	60	Asian	PDAC	*MSH6*	p.Leu782Pro	chr2:48027467	c.2345T>C	Missense	Novel	Negative	Negative
P2184	Male	53	Asian	PDAC	*ERBB2*	p.Met347fs	chr17:37868592	c.1039_1040insC	Frameshift	Novel	Negative	Negative
P2185	Female	70	Asian	PDAC	*FANCG*	p.Tyr290Cys	chr9:35076776	c.869A>G	Missense	Novel	Acute pancreatitis	Negative
P2191	Female	62	Asian	PDAC	*TNS3*	p.Leu520Phe	chr7:47408685	c.1558C>T	Missense	Novel	Negative	Negative
P2192	Female	63	Asian	PDAC	*ARID1A*	p.Gln1327fs	chr1:27100181	c.3999_4001delGCA	Frameshift	Novel	Negative	Negative
P2196	Male	66	Asian	PDAC	*RNF43*	p.Arg650Ter	chr17:56435189	c.1948C>T	Nonsense	Novel	Negative	Negative
P2208	Male	69	Asian	PDAC	*BRCA2*	p.Glu1213Asp	chr13:32912131	c.3639A>C	Missense	Novel	Negative	Negative
P2210	Female	47	Asian	PDAC	*TNS3*	p.Pro633Ser	chr7:47408346	c.1897C>T	Missense	Novel	Negative	Negative
P2211	Male	71	Asian	PDAC	*TNS3*	p.Glu231Lys	chr7:47451357	c.691G>A	Missense	Novel	Negative	Negative
P2218	Male	80	Asian	PDAC	*TNS3*	p.Arg158Trp	chr7:47463700	c.472C>T	Missense	Novel	Negative	Negative
P2222	Male	56	Asian	PDAC	*CTRC*	p.Arg119His	chr1:15769068	c.356G>A	Missense	VUS	Negative	Negative
P2222	Male	56	Asian	PDAC	*TERT*	p.Arg599Trp	chr5:1280428	c.1795C>T	Missense	Novel	Negative	Negative
P2224	Male	51	Asian	PDAC	*SPINK1*	p.Pro45Leu	chr5:147207645	c.134C>T	Missense	Novel	Negative	Negative
P2227	Male	60	Asian	PDAC	*MSH6*	p.Thr1282Ser	chr2:48033634	c.3845C>G	Missense	Novel	Negative	Negative
P2230	Female	64	Asian	PDAC	*CPA1*	p.Arg169Leu	chr7:130023254	c.506G>T	Missense	Novel	Negative	Negative

© 2022 Yin L et al. *JAMA Network Open*.

P2238	Male	80	Asian	PDAC	*FANCA*	p.Glu559Lys	chr16:89846317	c.1675G>A	Missense	VUS	Negative	Negative
P2238	Male	80	Asian	PDAC	*CFTR*	p.Asp984Asn	chr7:117246769	c.2950G>A	Missense	Novel	Negative	Negative
P2238	Male	80	Asian	PDAC	*PRSS1*	p.Trp57Ter	chr7:142458536	c.171G>A	Nonsense	Novel	Negative	Negative
P2243	Male	73	Asian	PDAC	*CTRC*	p.Val69Ile	chr1:15767061	c.205G>A	Missense	Novel	Negative	Negative
P2243	Male	73	Asian	PDAC	*PALB2*	p.Arg686Ser	chr16:23641417	c.2058G>T	Missense	VUS	Negative	Negative
P2244	Male	77	Asian	PDAC	*FANCA*	p.Val109Met	chr16:89877438	c.325G>A	Missense	Novel	Negative	Negative
P2248	Male	63	Asian	PDAC	*MSH2*	p.Val17Ile	chr2:47630379	c.49G>A	Missense	VUS	Negative	Negative
P2252	Female	61	Asian	PDAC	*CELA2A*	p.Val68Phe	chr1:15788128	c.202G>T	Missense	Novel	Negative	Negative
P2252	Female	61	Asian	PDAC	*MSH6*	p.Thr212Arg	chr2:48025757	c.635C>G	Missense	VUS	Negative	Negative
P2260	Male	59	Asian	PDAC	*BRIP1*	p.Asn643Ile	chr17:59857629	c.1928A>T	Missense	Novel	Negative	Negative
P2265	Male	66	Asian	PDAC	*ATM*	p.Phe2140Leu	chr11:108190753	c.6420C>A	Missense	VUS	Negative	Negative
P2276	Male	46	Asian	PDAC	*ERBB2*	p.Trp482Arg	chr17:37872123	c.1444T>C	Missense	Novel	Negative	Negative
P2280	Male	73	Asian	PDAC	*CFTR*	p.Gly1069Arg	chr7:117251700	c.3205G>A	Missense	VUS	Negative	Negative
P2294	Male	57	Asian	PDAC	*PALB2*	p.Ser1075Arg	chr16:23619310	c.3225T>A	Missense	VUS	Negative	Negative
P2308	Female	62	Asian	PDAC	*PMS2*	p.Cys192Ser	chr7:6038869	c.575G>C	Missense	Novel	Negative	Negative
P2312	Male	65	Asian	PDAC	*ATM*	p.Ile2878Met	chr11:108218055	c.8634A>G	Missense	VUS	Negative	Negative
P2322	Male	80	Asian	PDAC	*IDO2*	p.Ala96Thr	chr8:39836637	c.286G>A	Missense	Novel	Negative	Negative
P2324	Male	60	Asian	PDAC	*TNS3*	p.Leu520Phe	chr7:47408685	c.1558C>T	Missense	Novel	Negative	Negative
P2335	Male	64	Asian	PDAC	*CDH1*	p.Arg50His	chr16:68772300	c.149G>A	Missense	Novel	Negative	Negative
P2337	Female	66	Asian	PDAC	*BRCA2*	p.Ile3025Thr	chr13:32954007	c.9074T>C	Missense	VUS	Negative	Negative
P2353	Male	63	Asian	PDAC	*ARID1A*	p.Ala1522Thr	chr1:27101282	c.4564G>A	Missense	Novel	Negative	Negative
P2354	Male	72	Asian	PDAC	*BRCA2*	p.Ala2825Glu	chr13:32944681	c.8474C>A	Missense	VUS	Negative	Negative
P2371	Male	69	Asian	PDAC	*BRCA1*	p.Gln1096Arg	chr17:41244261	c.3287A>G	Missense	VUS	Negative	Negative
P2377	Male	65	Asian	PDAC	*IDO2*	p.Pro240Thr	chr8:39862857	c.718C>A	Missense	Novel	Negative	Negative
P2394	Male	66	Asian	PDAC	*ATM*	p.Asp2987Tyr	chr11:108235917	c.8959G>T	Missense	VUS	Negative	Negative
P2395	Male	85	Asian	PDAC	*MSH6*	p.Asn1273Lys	chr2:48033608	c.3819T>G	Missense	VUS	Negative	Negative
P2406	Female	66	Asian	PDAC	*TNS3*	p.Gly607Trp	chr7:47408424	c.1819G>T	Missense	Novel	Negative	Negative
P2409	Female	53	Asian	PDAC	*BUB1B*	p.Lys540fs	chr15:40494657	c.1619_1620insT	Frameshift	Novel	Negative	Negative
P2410	Male	67	Asian	PDAC	*HNF1B*	p.Ser80Cys	chr17:36104637	c.239C>G	Missense	Novel	Negative	Negative
P2428	Female	42	Asian	PNET	*RNF43*	p.Arg668Trp	chr17:56435135	c.2002C>T	Missense	Novel	Negative	Negative
P2441	Female	64	Asian	PNET	*XRCC3*	p.Arg150Cys	chr14:104169623	c.448C>T	Missense	Novel	Negative	Negative
P2448	Male	47	Asian	PNET	*STK11*	p.Pro387Arg	chr19:1226504	c.1160C>G	Missense	Novel	Negative	Negative

© 2022 Yin L et al. *JAMA Network Open*.

P2449	Male	70	Asian	PNET	*PALB2*	p.Arg686Ser	chr16:23641417	c.2058G>T	Missense	VUS	Negative	Negative
P2450	Male	44	Asian	PNET	*BRCA1*	p.Asp1578Gly	chr17:41226353	c.4733A>G	Missense	VUS	Negative	Negative
P2450	Male	44	Asian	PNET	*PALB2*	p.Asp360Asn	chr16:23646789	c.1078G>A	Missense	Novel	Negative	Negative
P2453	Male	49	Asian	Chronic pancreatitis	*FANCC*	p.Thr503Met	chr9:97869373	c.1508C>T	Missense	VUS	Negative	Negative
P2455	Female	36	Asian	Acute pancreatitis	*BRIP1*	p.Ser1109Ile	chr17:59761081	c.3326G>T	Missense	Novel	Negative	Negative
P2456	Male	36	Asian	Chronic pancreatitis	*MSH2*	p.Ile511Phe	chr2:47693817	c.1531A>T	Missense	Novel	Negative	Negative
P2458	Male	66	Asian	PNET	*PALB2*	p.Pro113Ala	chr16:23647530	c.337C>G	Missense	VUS	Negative	Uncle with gastric cancer
P2471	Female	72	Asian	IPMN	*BUB1*	p.Phe962Val	chr2:111398682	c.2884T>G	Missense	Novel	Lung cancer	Negative
P2472	Female	31	Asian	IPMN	*CELA3B*	p.Val198Met	chr1:22310774	c.592G>A	Missense	Novel	Negative	Negative
P2482	Male	41	Asian	Acute pancreatitis	*MLH1*	p.Ser577Leu	chr3:37083821	c.1730C>T	Missense	VUS	Negative	Negative
P2482	Male	41	Asian	Acute pancreatitis	*RECQL4*	p.Arg780Trp	chr8:145738726	c.2338C>T	Missense	VUS	Negative	Negative
P2483	Female	73	Asian	Acute pancreatitis	*HNF4G*	p.Ala213Thr	chr8:76468238	c.637G>A	Missense	Novel	Negative	Negative
P2501	Male	66	Asian	Acute pancreatitis	*ATM*	p.Leu1956His	chr11:108180991	c.5867T>A	Missense	VUS	Negative	Negative
P2508	Female	60	Asian	Acute pancreatitis	*FANCG*	p.Ser387fs	chr9:35075737	c.1157_1158insG	Frameshift	Novel	Negative	Negative
P2525	Female	29	Asian	SCN	*ARID1A*	p.Gly2040Arg	chr1:27106507	c.6118G>A	Missense	Novel	Negative	Negative
P2526	Female	58	Asian	SCN	*RECQL4*	p.Leu828Gln	chr8:145738502	c.2483T>A	Missense	Novel	Negative	Negative
P2544	Male	59	Asian	SPT	*ATM*	p.Leu846Val	chr11:108137967	c.2536C>G	Missense	Novel	Negative	Negative
P2545	Female	75	Asian	Cholangiocarcinoma	*BUB1B*	p.Lys539fs	chr15:40494654	c.1616_1617insT	Frameshift	Novel	Negative	Negative
P2547	Male	62	Asian	IPMN	*CFTR*	p.Gly1069Arg	chr7:117251700	c.3205G>A	Missense	VUS	Negative	Negative
P2558	Male	27	Asian	IPMN	*TNS3*	p.Leu520Phe	chr7:47408685	c.1558C>T	Missense	Novel	Negative	Negative
P2564	Female	39	Asian	PNET	*CEL*	p.Ala434fs	chr9:135945850	c.1298_1299insC	Frameshift	Novel	Negative	Negative
P2568	Female	60	Asian	PDAC	*CEL*	p.Arg119Trp	chr9:135940432	c.355C>T	Missense	Novel	Negative	Negative
P2571	Female	62	Asian	PNET	*ARID1A*	p.Pro1169Ala	chr1:27099089	c.3505C>G	Missense	Novel	Negative	Negative
P2571	Female	62	Asian	PNET	*FANCA*	p.Leu521Phe	chr16:89849420	c.1561C>T	Missense	Novel	Negative	Negative
P2573	Female	42	Asian	MCN	*BRCA2*	p.Ter3419fs	chr13:32972904	c.10255_10256insT	Frameshift	Novel	Negative	Negative
P2595	Male	77	Asian	Chronic pancreatitis	*PALB2*	p.Val805Ala	chr16:23641061	c.2414T>C	Missense	Novel	Negative	Negative

© 2022 Yin L et al. *JAMA Network Open*.

eTable 5. Clinicopathologic Characteristics of patients with germline mutation and wild-type cases.

Characteristics	With Deleterious Germline Variant (n=63)	Wild-type (n=946)	*p Value*
Age, years			
Mean (SD)	63.4 (9.9)	62.8 (10.3)	0.88
Gender, n (%)			
Male	47	580	0.04
Female	16	366	
Family History of PDAC			0.44
Yes	1	8	
Location of tumor			
Head/Neck	44	653	1.00
Body/Tail	19	253	
Stage			
Resectable/Borderline resectable	22	297	0.58
Locally advanvced /Metastatic	41	649	

© 2022 Yin L et al. *JAMA Network Open*.

eTable 6. Comparison of Nanjing cohort and Johns Hopkins Cohort on the pathogenic germline mutations of PDAC patients.					
**Gene**	**Nanjing Cohort**		**Johns Hopkins Cohort[b]**		***P* value[a]**
	**Cases with Germline mutation**	**Total cases**	**Cases with Germline mutation**	**Total cases**	
ATM	5	1,009	10	854	0.12
BRCA1	3	1,009	2	854	1.00
BRCA2	9	1,009	12	854	0.38
PALB2	6	1,009	2	854	0.30
BUB3	0	1,009	1	854	0.46
BRIP1	3	1,009	0	854	0.26
CDH1	0	1,009	1	854	0.46
CDKN2A	0	1,009	1	854	0.46
FANCC	2	1,009	0	854	0.50
RAD51B	0	1,009	1	854	0.46
RAD51D	3	1,009	1	854	0.63
TP53	1	1,009	1	854	1.00
XRCC2	1	1,009	0	854	1.00
[a], Remain significant at $P<0.004$ if Bonferroni correction is used.					
[b], Clinical significance of variants was re-evaluated and updated to July 2020.					

© 2022 Yin L et al. *JAMA Network Open*.

eTable 7. Prevalence of pathogenic germline mutations in non-PDAC and Chinese population.									
**Disease**	**Gene**	**Chromosome Position**	**Amino Acid Change**	**Nucleotide Change**	**Function**	**Gene group**	**Nanjing Allele Count (%)**	**ChinaMap Allele Count (%)**	***P*-value**
IPMN	*ATM*	chr11:108151896	Splice	c.3576+1G>A	Noncoding	Pancreatic cancer susceptibility genes	1/140 (0.71)	0/21,176 (0)	< 0.01
IPMN	*RECQL4*	chr8:145738229	Splice	c.2755+1G>A	Noncoding	Candidate pancreatic cancer susceptibility genes	1/140 (0.71)	0/21,176 (0)	< 0.01
Acute pancreatitis	*CFTR*	chr7:117149143	p.Arg74Trp	c.220C>T	Missense	Pancreatitis associated genes	2/710 (0.28)	20/21,176 (0.09)	0.340
Cholangiocarcinoma	*PALB2*	chr16:23625413	Splice	c.3114-1G>A	Noncoding	Pancreatic cancer susceptibility genes	1/70 (1.43)	1/21,176 (0.005)	< 0.01
Chronic pancreatitis	*BRIP1*	chr17:59937230	p.Leu43fs	c.128_131delTGTT	Frameshift	Candidate pancreatic cancer susceptibility genes	1/196 (0.51)	1/21,176 (0.005)	< 0.01
Chronic pancreatitis	*PRSS1*	chr7:142459789	p.Arg122His	c.365G>A	Missense	Pancreatic cancer susceptibility genes	1/196 (0.51)	0/21,176 (0)	< 0.01
Chronic pancreatitis	*SPINK1*	chr5:147207583	Splice	c.194+2T>C	Noncoding	Pancreatitis associated genes	19/196 (9.69)	114/21,176 (0.54)	< 0.01
PNET	*BRCA1*	chr17:41243677	p.Lys1290fs	c.3869_3870delAA	Frameshift	Pancreatic cancer susceptibility genes	1/238 (0.42)	0/21,176 (0)	0.01
PNET/Acute pancreatitis	*CFTR*	chr7:117246728	p.Gly970Asp	c.2909G>A	Missense	Pancreatitis associated genes	2/948 (0.21)	15/21,176 (0.07)	0.36
SCN	*BRCA2*	chr13:32913456	p.Tyr1655Ter	c.4965delC	Nonsense	Pancreatic cancer susceptibility genes	1/64 (1.56)	0/21,176 (0)	< 0.01
SCN	*CFTR*	chr7:117175301	Splice	c.580-1G>T	Noncoding	Pancreatitis associated genes	1/64 (1.56)	0/21,176 (0)	< 0.01
SPT	*ATM*	chr11:108121593	p.Lys468fs	c.1402_1403delAA	Frameshift	Pancreatic cancer susceptibility genes	1/112 (0.89)	1/21,176 (0.005)	< 0.01
SPT	*PALB2*	chr16:23634452	Splice	c.2835-1G>C	Noncoding	Pancreatic cancer susceptibility genes	1/112 (0.89)	0/21,176 (0)	< 0.01
IPMN, intraductal papillary mucinous neoplasm; SCN, serous cystic noplasm; SPT, solid pseudopapillary tumor; PNET, pancreatic neuroendocrine tumor.									

© 2022 Yin L et al. *JAMA Network Open*.
